# The microbiome and brain metastasis in non‐small cell lung cancer: A graded appraisal and a framework for prevention‐oriented research

**DOI:** 10.1002/ctm2.70862

**Published:** 2026-11-01

**Authors:** Long Liang, Fangjuan Hu, Xiaofeng Wang, Chunhan Tang, Chun Wang, Gefei Jiang, Chungang Zhao, Yin Zhang, Churong Li

**Affiliations:** ^1^ Department of Radiation Oncology Sichuan Clinical Research Center for Cancer Sichuan Cancer Hospital & Institute Sichuan Cancer Center University of Electronic Science and Technology of China Chengdu China; ^2^ Oncology Department II Qingdao Central Hospital University of Health and Rehabilitation Sciences Qingdao China; ^3^ Department of Oncology The First Affiliated Hospital of Chengdu Medical College Chengdu Sichuan China; ^4^ Department of Radiology Dazhou Central Hospital Sichuan Clinical Research Center for Medical Imaging (Dazhou) Dazhou China; ^5^ Oncology Department Dazhou Central Hospital Sichuan Clinical Research Center for Medical Imaging (Dazhou) Dazhou China

**Keywords:** brain metastasis, evidence appraisal, immunotherapy, MAIT cells, microbiome, non‐small cell lung cancer, premetastatic niche, tumour microenvironment

## Abstract

**Background:**

Brain metastasis remains a major cause of mortality and treatment failure in non‐small cell lung cancer (NSCLC). However, current models of cerebral dissemination rarely integrate microbiome‐dependent systemic and intratumoral biology.This Review examines the potential microbiome‐brain metastasis axis linking gut, lung, and intratumoral microbial communities to intracranial dissemination and therapeutic response.

**Methods:**

A reproducible literature search was conducted to evaluate proposed mechanisms underlying microbiome‐associated brain metastasis. Evidence for each mechanistic claim was graded using a four‐level evidence scheme (E1‐E4), while microbial detection confidence was assessed using a four‐tier hierarchy (D1‐D4).

**Results:**

No component of the proposed axis reached E1 evidence, and all published microbial observations in NSCLC brain metastasis rests on nucleic‐acid detection alone. Five mechanisms are examined: circulating microbial metabolites acting on endothelial permeability and glial reactivity; microbiome‐imprinted immune‐cell trafficking;intracellular bacteria within primary lung tumours; cross‐kingdom MR1/MAIT and Dectin‐CARD9 signalling; and candidate host genetic determinants. The available human data are small, single‐centre and cross‐sectional, reporting association with brain‐metastasis status rather than prediction or causation; that bacteria accompany disseminating tumour cells into the brain remains a hypothesis awaiting paired sampling, spatial localization and experimental perturbation.We therefore reserve the term prevention for proposed trials, integrate microbial features with established molecular risk models, and specify the genetic‐perturbation program and research‐only decision pathway that would convert these claims into E1 evidence.

**Conclusions:**

The microbiome–brain metastasis axis provides a hypothesis‐generating framework for investigating microbial contributions to cerebral dissemination. Multi‐centre validation, paired primary‐brain metastasis profiling and gnotobiotic models are required before microbiome‐directed strategies can be said to improve intracranial outcomes;until then this framework is hypothesis‐generating, not practice‐changing.

**Key points:**

- The proposed microbiome–brain metastasis axis links gut, lung and intratumoral microbial communities to intracranial dissemination in NSCLC.
- Current evidence remains insufficient to establish causality, with no proposed mechanism reaching the highest evidence level (E1).
- Five candidate mechanisms involve microbial metabolites, immune‐cell trafficking, intratumoral bacteria, MR1/MAIT and Dectin‐CARD9 signalling, and host genetic determinants.
- Multicentre validation, paired primary and brain metastasis profiling, spatial microbial localisation and experimental perturbation are needed to establish causality and assess clinical relevance.
- Microbiome‐directed prevention and therapeutic strategies remain investigational and should not yet inform routine clinical practice.

## Graphical abstract

This Review proposes a microbiome–premetastatic brain niche axis connecting gut, lung, and intratumoral microbes with metabolites, blood–brain barrier remodeling, neuroimmune priming, and brain metastasis in NSCLC. It integrates current evidence while distinguishing established mechanisms from associations and hypotheses requiring prospective and mechanistic validation.

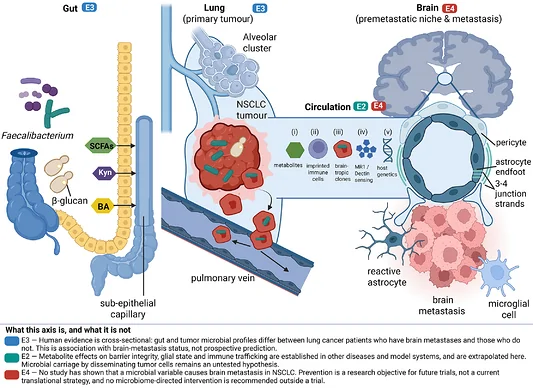

## 1 INTRODUCTION

### 1.1 The clinical urgency of brain metastasis in non‐small cell lung cancer

Lung cancer remains the leading cause of cancer‐related mortality worldwide, accounting for approximately 1.8 million deaths annually. Non‐small cell lung cancer (NSCLC) comprises 80%–85% of lung cancer diagnoses, and brain metastasis represents one of its most devastating complications. Thirty to fifty percent of NSCLC patients develop brain metastases during the course of their disease,^1^, ^2^, ^3^ with the highest rates observed in patients with adenocarcinoma harbouring EGFR mutations, ALK fusions, or ROS1 rearrangements.^4^, ^5^ Once established, brain metastasis confers a median overall survival of 7–12 months,^6^ with outcomes that have improved only modestly despite the introduction of stereotactic radiosurgery, third‐generation tyrosine kinase inhibitors (osimertinib for EGFR‐mutant disease, lorlatinib for ALK‐positive disease), and immune checkpoint inhibitors. The biology of cerebral colonization presents a unique challenge: tumour cells must survive shear forces in circulation, breach the blood–brain barrier (BBB), evade brain‐resident immune surveillance by microglia and astrocytes, and establish productive proliferation in a metabolically distinct microenvironment. The cerebral compartment is also pharmacologically privileged: many systemic anti‐cancer agents penetrate the BBB poorly, and CNS‐active formulations remain limited. These constraints have driven intensive investigation into the premetastatic niche, the concept introduced by Lyden, Kaplan, and colleagues, in which primary tumour‐derived factors (cytokines, exosomes, mobilized bone marrow cells) condition distant organs for tumour cell colonization before metastatic seeding occurs. In the brain, premetastatic niche formation involves astrocyte activation, microglial priming, BBB tight junction remodeling, and recruitment of myeloid‐derived suppressor cells. Yet despite this rich cellular and molecular framework, one major contributor to systemic biology has been almost entirely omitted: the human microbiome.

### 1.2 The microbiome era in oncology

In the past decade, the human microbiome has emerged as a central regulator of cancer initiation, progression, and treatment response. The 2022 update to Hanahan's hallmarks of cancer added polymorphic microbiomes as an enabling characteristic, formalizing what a series of landmark studies had established. Gut microbiome composition is associated with response to immune checkpoint inhibitors (ICIs) across NSCLC, melanoma, renal cell carcinoma and gastrointestinal malignancies, and this association has been reproduced in independent European and North American cohorts.^7^, ^8^, ^9^ Specific gut bacterial species, notably *Akkermansia muciniphila*, *Faecalibacterium prausnitzii*, and *Bifidobacterium* spp., are enriched in checkpoint inhibitor responders,^7^, ^8^, ^9^ while gut dysbiosis induced by broad‐spectrum antibiotics or proton pump inhibitors abrogates therapeutic efficacy. Mechanistic studies in mice demonstrate that gut microbes modulate immunotherapy response through CD8+ T cell priming, dendritic cell maturation, regulatory T cell differentiation, and innate lymphoid cell activation, and that defined consortia or single microbial metabolites are sufficient to restore ICI efficacy in germ‐free or antibiotic‐treated animals.^10^, ^11^, ^12^ A second line of work developed with the recognition that microbes can be detected not only in the gut but also within tumours themselves. Pan‐cancer analyses have reported that intratumoral microbial communities^13^ are detectable across at least seven cancer types, including breast, lung, ovarian, pancreatic, bone, and brain tumours, and melanoma; we note in Section 5.8 that this literature has also produced a prominent retraction arising from contamination and analytic error,^14^, ^15^ which is why detection claims are graded separately throughout this Review. Subsequent work has reported that intracellular bacteria within tumour cells modulate cellular phenotypes^16^, ^17^ including immune evasion and metastatic propensity. In lung cancer specifically, oral commensal taxa are enriched in lung tumours, plausibly through microaspiration and impaired clearance, and their enrichment is associated with local inflammation,^18^ and subsequent studies have refined the lung tumour microbiome signature and reported associations with primary disease behaviour and distant metastasis.

### 1.3 Three converging lines of evidence

Three lines of evidence now motivate a unified examination of microbial contributions to NSCLC brain metastasis. First, the gut microbiome‐immunotherapy response axis is well replicated for NSCLC: pretreatment gut microbiome composition is associated with immune checkpoint inhibitor response and progression‐free survival in lung adenocarcinoma cohorts, and microbiome‐modulating interventions, including probiotic supplementation, faecal microbiota transplantation and dietary fibre modulation, are entering clinical trials as adjuvants to checkpoint blockade. The brain metastasis subgroup of NSCLC patients receiving checkpoint inhibitors shows differential intracranial response rates relative to extracranial lesions; whether gut microbiome features explain any of this variability is an open question that has not been tested in a cohort with intracranial response as the endpoint. Second, the intratumoral microbiome of primary lung tumours is a candidate metastatically active compartment. Intratumoral bacterial load and composition differ between NSCLC histologies, and specific taxa correlate with immune infiltration and recurrence risk. Recent single‐cell analyses have provided a striking observation: primary NSCLC tumours are dominated by fungi‐associated cells, whereas brain metastatic lesions are dominated by bacteria‐associated cells,^19^ a kingdom‐level compositional shift that has been interpreted as selective survival, transport, or recruitment pressures during metastatic dissemination. We emphasize at the outset that the specimens compared in that analysis were assembled from independently generated public datasets rather than sampled within the same patients, so the shift is at present a hypothesis rather than an established biological phenomenon (Sections 5.8 and 6.5). Third, gut microbiomes have been compared between lung cancer patients who do and do not have brain metastases. Two recent single‐centre metagenomic studies from the same group^20^, ^21^ have reported that gut microbiome signatures differ between lung cancer patients with versus without brain metastasis: in NSCLC, *Paenibacillus*, *Fournierella* and *Adlercreutzia* were enriched in the brain metastasis group with altered short‐chain fatty acid and tryptophan‐derivative metabolite profiles^20^; in SCLC, *Marvinbryantia* depletion and benzoic acid emerged as the top machine learning‐derived discriminators of brain‐metastasis status, with *Streptococcus* enrichment also observed.^21^ A separate cohort reported that gut microbiome composition is associated with radiotherapy response in NSCLC patients with *established* brain metastasis^22^; this is a treatment–response endpoint in patients who already have intracranial disease, and it is not evidence about brain‐metastasis occurrence, prevention, or intracranial immunotherapy response. These findings rest on modest single‐centre discovery cohorts that await independent replication, and each addresses a different and non‐interchangeable endpoint: cross‐sectional discrimination of brain‐metastasis status^20^, ^21^ and radiotherapy response in established disease.^22^ None addresses whether microbial alterations precede, predict, or cause brain metastasis. They nevertheless motivate the mechanistic integration and, more importantly, the evidence appraisal that we attempt here.

### 1.4 The proposed microbiome–brain metastasis axis

These developments converge on a tractable conceptual framework that we term the microbiome–brain metastasis axis. Our central hypothesis, and we stress that it is a hypothesis, is that gut and intratumoral microbiomes may contribute to priming of the cerebral premetastatic niche through five candidate mechanisms: (i) circulating microbial metabolites, including short‐chain fatty acids, tryptophan‐derived indoles and kynurenines, secondary bile acids, and bacterial lipopolysaccharide‐derived signals, that remodel BBB tight junction integrity and microglial activation state; (ii) microbiome‐modulated trafficking of MR1‐restricted mucosal‐associated invariant T (MAIT) cells, natural killer cells, monocytes, and myeloid‐derived suppressor cells to the cerebral compartment, where they shape the premetastatic immune landscape; (iii) intratumoral bacteria within primary lung tumours that might promote selection of brain‐tropic metastatic clones through direct effects on epithelial‐mesenchymal transition, immune evasion, and metabolic reprogramming; (iv) cross‐kingdom MR1/MAIT and Dectin–CARD9 signalling at the gut–lung–brain interface, linking microbial vitamin B2 metabolism and fungal β‐glucan recognition to anti‐tumour immunity; and (v) host genetic determinants that simultaneously shape microbiome composition (CARD9, CLEC7A, FUT2) and brain metastasis susceptibility (EGFR, ALK, TP53, HLA variants). Throughout the Review these five mechanisms are grouped into five numbered *nodes*, and the numbering used in every figure and table is that of Figure 1b: **Node 1**, the gut microbiome; **Node 2**, the lung and intratumoral microbiome; **Node 3**, microbial metabolites and the blood–brain barrier; **Node 4**, cross‐kingdom MR1/MAIT and Dectin–CARD9 signalling; **Node 5**, host genetic modification of the axis. Mechanism (ii), immune‐cell trafficking, is the shared output of Nodes 1 and 4 rather than a node of its own. The one exception is the therapeutic‐landscape panel of Section 8, whose row labels belong to the original artwork and denote compartments rather than these numbered nodes; the mapping is given in that figure's legend. The one exception is the therapeutic landscape of Section 8, which groups interventions by therapeutic target rather than by node; that grouping is reconciled to this scheme in its own figure legend.

**FIGURE 1:**
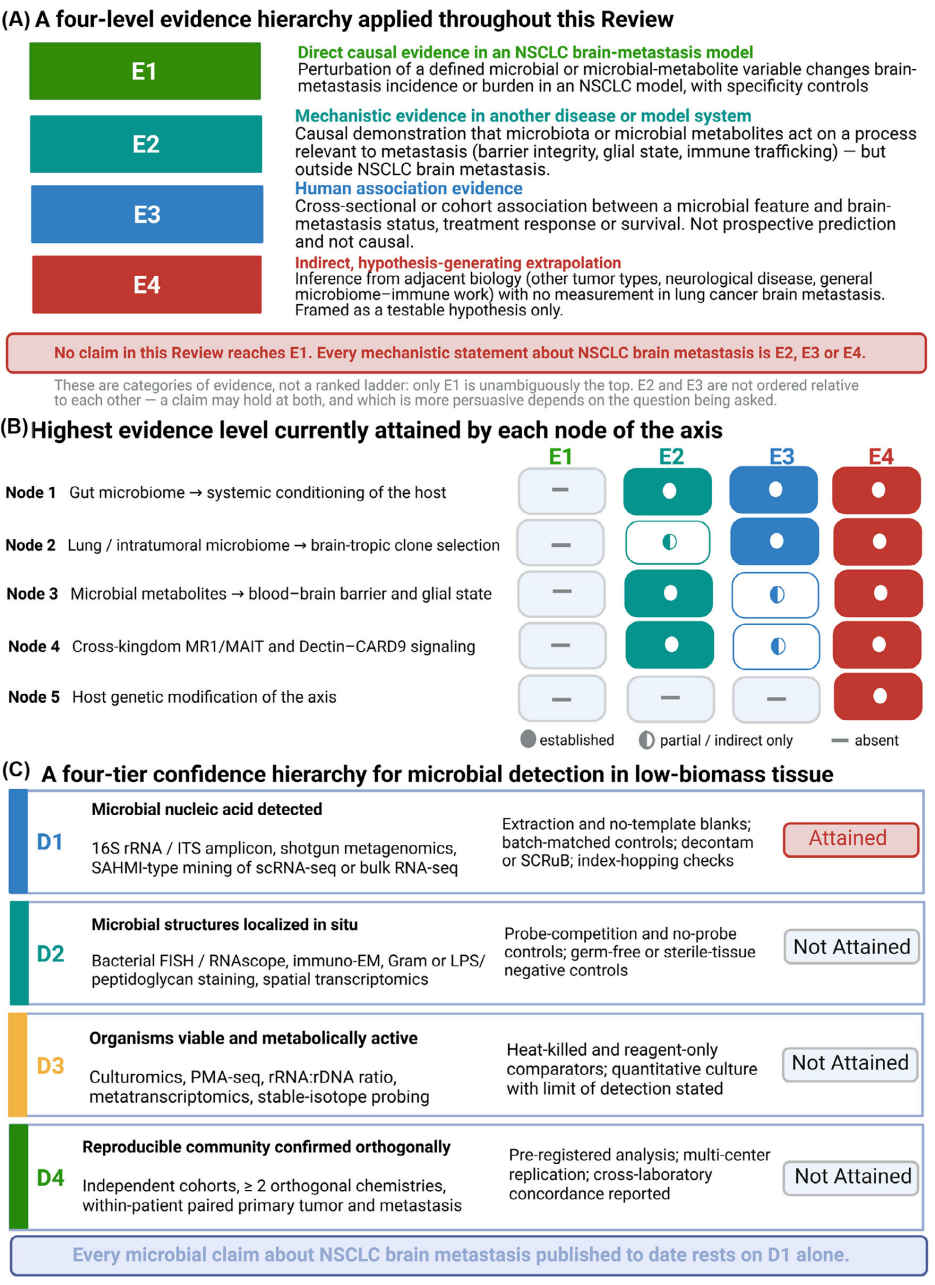
Evidence architecture of the microbiome–brain metastasis axis. (A) The four‐level evidence hierarchy applied throughout this Review, from direct causal evidence in an NSCLC brain‐metastasis model (E1) to indirect hypothesis‐generating extrapolation (E4), with the operational definition of each level. (B) The highest evidence level currently attained by each of the five nodes of the axis. Filled cells denote established evidence at that level, half‐filled cells partial or indirect evidence only, and dashes absence of evidence. The E1 column is empty for every node: no microbial or metabolite variable has been shown to change brain‐metastasis incidence or burden in an NSCLC model. (C) The four‐tier detection‐confidence hierarchy applied to every claim about the physical presence of microbes, with the minimum controls required at each tier and the tier currently attained in NSCLC brain metastasis. All published claims rest on D1, nucleic‐acid detection. Figure prepared by the authors; no previously published material is reproduced.

Because the five nodes rest on evidence of very different quality, a central purpose of this Review is to state that quality explicitly rather than to assert the axis. Section 2 sets out the literature search and the two grading schemes we apply throughout: a four‐level evidence hierarchy (E1–E4) and a four‐tier microbial detection‐confidence hierarchy (D1–D4). Figure 1 summarizes the result of applying them. The headline finding of that exercise is negative and should be stated plainly here: no node of the axis currently attains E1, and every microbial observation reported in NSCLC brain metastasis rests on D1 (nucleic‐acid detection) alone. This framework yields specific, falsifiable predictions: that gut and intratumoral microbial signatures collected prior to metastatic dissemination would predict brain metastasis occurrence with clinically actionable accuracy; that microbiome‐modulating interventions would reduce brain metastasis incidence and improve intracranial immunotherapy response rates; that the lung‐to‐brain microbial compositional shift (fungi‐dominant to bacteria‐dominant) reflects directed selection rather than stochastic drift or differential technical background; that specific microbial metabolites mechanistically prime astrocyte and microglial activation and BBB permeability changes in advance of metastatic seeding; and that host genetic stratification will identify patient subgroups in which microbiome‐targeted prevention is disproportionately effective. Each mechanism can be tested by gnotobiotic mouse experiments, prospective cohort metagenomics, single‐cell spatial transcriptomics paired with kingdom‐resolved microbial detection, or interventional trials of microbiome‐modulating therapy combined with checkpoint blockade or radiotherapy, and Section 7 specifies the genetic‐perturbation designs required to establish specificity.

### 1.5 Roadmap

This review is organized around four contributions. Section 2 reports the literature search and the evidence‐ and detection‐grading schemes that govern every subsequent claim. Sections 2.6, 3.2, 4.6, 5.8.1 present the biology and the human data: host genetic architecture (Section 3), the axis mechanisms themselves (Section 4), cross‐compartment gut–lung–brain communication and the methodological limits of low‐biomass microbial detection (Section 5), and the clinical evidence, including how microbial features compare with established molecular risk models for NSCLC brain metastasis (Section 6). Section 7 specifies the gene‐knockout and genetic‐perturbation experiments required to establish target specificity. Section 8 sets out translational implications and an explicit, research‐only clinical decision pathway for the high‐risk NSCLC subgroup, and Section 9 gives the research roadmap. We note that an earlier review by Jiang et al.^23^ catalogued microbiome alterations in NSCLC and brain metastasis with a focus on candidate markers and drug targets; the present work differs in scope by (i) introducing a five‐node mechanistic axis framework rather than a descriptive catalogue, (ii) integrating intratumoral microbiome biology and the recently described lung‐to‐brain fungi‐to‐bacteria compositional shift, (iii) incorporating host genetic architecture as a constraining layer, (iv) treating cross‐kingdom MR1/MAIT and Dectin–CARD9 signalling in detail, (v) articulating ten falsifiable predictions with decisive readouts to enable structured experimental testing, and (vi) grading every claim against a pre‐specified evidence hierarchy rather than presenting mechanism and speculation in a single register. Throughout, we emphasize where the field is constrained by methodology, where causal claims remain underdetermined, and where targeted experimental work could yield disproportionate progress.

## 2 METHODS: LITERATURE SEARCH, EVIDENCE APPRAISAL AND TERMINOLOGY

### 2.1 Design and reporting

This article is a narrative, mechanism‐oriented review conducted with a systematic and reproducible search. It is not a systematic review or meta‐analysis, and we make no claim that it satisfies PRISMA 2020^24^ in full; the heterogeneity of the underlying literature, which spans human observational cohorts, computational reanalyses of public sequencing data, gnotobiotic animal work and in vitro mechanistic studies, makes quantitative synthesis inappropriate. We instead followed PRISMA 2020^24^ for the reporting of information sources and search strategy, and the SANRA criteria^25^ for narrative‐review quality. The purpose of the procedures described below is to allow a reader to reproduce our retrieval, to see exactly which studies support which statement, and to see the grade we assign to every claim.

### 2.2 Information sources and search strategy

We searched PubMed/MEDLINE, Embase, Web of Science Core Collection, Scopus and the Cochrane Central Register of Controlled Trials from database inception to 31 July 2026, with no language restriction at the search stage. ClinicalTrials.gov and the WHO International Clinical Trials Registry Platform were searched for registered interventional studies of microbiome‐modulating therapy in lung cancer. Conference proceedings (ASCO, ESMO, AACR, WCLC) from 2020 onward were hand‐searched for unpublished cohorts. Reference lists of all included articles and of prior reviews, including Jiang et al.^23^, were screened forward and backward. The search combined three concept blocks with the Boolean operator AND: (i) lung cancer terms; (ii) brain metastasis, central nervous system, blood–brain barrier and neuroimmune mechanism terms, placed in one block rather than two so that mechanistic work carrying no metastasis term would still be retrieved; and (iii) microbiome, mycobiome, metabolite and specific taxon terms. The search strategy for each database is reported in Table 1; the PubMed/MEDLINE strings are printed in full and as executed, and the adaptations for the other databases are described in the table and reproduced verbatim in the supplementary search log. The PubMed/MEDLINE strings and yields are final and reproducible as printed. The search was re‐run on 31 July 2026, immediately before submission, to capture recent publications.

**TABLE 1:** Databases, search strings and date ranges for the literature search.

Source	Search string / approach	Date range	Contribution to the review
PubMed / MEDLINE — Search A (direct) (29 records)	(“lung neoplasms”[MeSH] OR “non‐small cell lung”[tiab] OR NSCLC[tiab] OR “lung cancer”[tiab] OR “lung adenocarcinoma”[tiab] OR “lung carcinoma”[tiab]) AND (“brain neoplasms”[MeSH] OR “brain metastas*”[tiab] OR “cerebral metastas*”[tiab] OR “CNS metastas*”[tiab] OR leptomeningeal[tiab] OR “blood‐brain barrier”[MeSH] OR “blood‐brain barrier”[tiab] OR neuroinflammation[tiab] OR microglia[tiab] OR astrocyte*[tiab] OR “premetastatic niche”[tiab]) AND (microbiome[tiab] OR microbiota[tiab] OR mycobiome[tiab] OR dysbiosis[tiab] OR “short‐chain fatty acid*”[tiab] OR butyrate[tiab] OR “bile acid*”[tiab] OR kynurenine[tiab] OR “tryptophan metabolit*”[tiab] OR lipopolysaccharide[tiab] OR “fecal microbiota transplantation”[tiab] OR probiotic*[tiab] OR “MAIT cell*”[tiab] OR MR1[tiab] OR CARD9[tiab] OR Dectin[tiab] OR “intratumoral bacteria”[tiab] OR “intratumour* microbi*”[tiab]) AND 1800:2026/07/31[dp]	Inception to 31 July 2026	The entire directly relevant literature. Its size is itself a finding and constrains every claim in this Review.
PubMed / MEDLINE — Search B (lung cancer and microbiome) (1984 records)	Block 1 AND Block 3, as above, with the same date limit	Inception to 31 Jul 2026	Lung‐cancer microbiome biology and immunotherapy‐response cohorts (evidence level E3).
PubMed / MEDLINE — Search C (CNS, barrier and microbiome, restricted to neoplastic context) (2062 records)	(“brain metastas*”[tiab] OR “premetastatic niche”[tiab] OR “blood‐brain barrier”[tiab] OR microglia[tiab] OR astrocyte*[tiab]) AND Block 3 AND (cancer[tiab] OR tumor[tiab] OR tumour[tiab] OR neoplasm*[tiab] OR metasta*[tiab] OR carcinoma[tiab]) AND 1800:2026/07/31[dp]	Inception to 31 July 2026	Mechanistic work performed outside NSCLC brain metastasis (evidence level E2).
PubMed / MEDLINE — union of Searches A, B and C (4,020 records)	(A OR B OR C), deduplicated within PubMed	Inception to 31 July 2026	Records entering title and abstract screening from PubMed.
Embase	Blocks 1–3 adapted to Emtree syntax with explosion	Inception to 31 July 2026	Additional European and pharmacological sources.
Web of Science Core Collection	Blocks 1–3 as topic (TS) search	Inception to 31 July 2026	Citation chaining and conference‐indexed records.
Scopus	Blocks 1–3 as TITLE‐ABS‐KEY search	Inception to 31 July 2026	Cross‐check for records missed by the above.
Cochrane CENTRAL	Blocks 1 and 3, intervention terms	Inception to 31 July 2026	Randomized and controlled interventional evidence.
ClinicalTrials.gov and WHO ICTRP	Condition: lung cancer; intervention: probiotic, FMT, microbiome, diet, butyrate	Inception to 31 July 2026	Registered but unpublished interventional studies; the registrations retrieved are summarized in Section 6.3 and no completed trial with a brain‐metastasis endpoint was identified.
Conference proceedings (ASCO, ESMO, AACR, WCLC)	Hand search of abstract books	2020 to 2026	Unpublished cohorts; none met eligibility for a factual claim.
Reference‐list and citation chaining	Backward and forward screening of all included records and of Jiang et al.^23^	Not applicable	Recovery of older mechanistic primary sources.
**Total after deduplication and screening**			**Records cited in this Review: 160, of which 3 report primary human data in lung cancer brain metastasis** ^20^, ^21^, ^22^ **and 1 is a computational reanalysis of separately generated public datasets**.^19^

*Note*: The PubMed strings are printed as executed and can be pasted into PubMed unchanged; Searches B and C refer to the blocks defined in Search A. The Embase, Web of Science and Scopus adaptations are reproduced verbatim in the supplementary search log. MeSH terms were exploded where available and combined with free‐text title/abstract terms.

### 2.3 Eligibility criteria

Records were eligible if they reported original data relating microbial communities, microbial‐derived metabolites, or host genetic determinants of microbial ecology to one or more of the following in lung cancer or in a directly relevant model system: brain‐metastasis occurrence or burden; blood–brain barrier structure or permeability; glial or CNS‐resident immune cell state; immune‐cell trafficking to the central nervous system; metastatic dissemination or organotropism; or intracranial response to systemic or CNS‐directed therapy. Mechanistic studies conducted outside NSCLC brain metastasis were eligible but were, by construction, assigned to evidence level E2 or E4 (Section 2.5). Records were excluded if they were non‐English without an available translation, were case reports of fewer than five patients, reported only 16S amplicon data without any contamination control in a low‐biomass compartment, were duplicate reports of a single cohort (in which case the most complete report was retained), or were preprints that had not undergone peer review at the time of the final search. Editorials, commentaries and prior reviews were not used as graded evidence for any mechanistic, clinical or therapeutic claim; where a review is cited it supports background or definitional statements only, and the primary study is cited alongside it wherever the claim is graded.

### 2.4 Study selection, data extraction and independence

Titles and abstracts were screened independently and in duplicate by two authors (L.L. and F.H.), and full texts of all potentially eligible records were assessed independently by the same two authors. Disagreements were resolved by discussion, and unresolved disagreements were adjudicated by a third author (X.W.). Data extraction was performed independently and in duplicate using a piloted form capturing study design, cohort size, comparator group definition, sequencing or assay platform, contamination control procedures, endpoint definition, effect estimate, adjustment for antibiotic, corticosteroid and proton pump inhibitor exposure, and funding source. Evidence‐level and detection‐tier assignment (Sections 2.5 and 2.6) was likewise performed independently and in duplicate, with disagreements adjudicated by the senior author (C.L.).

### 2.5 Evidence‐level classification

A recurring weakness of the microbiome–cancer literature is that mechanistic plausibility is presented in the same register as demonstrated mechanism. To prevent this, we assign every substantive claim in this Review to one of four levels, defined a priori and applied uniformly in the text, figures and tables (Table 2, Figure 1a).

**TABLE 2:** Definitions of the four evidence levels (E1–E4) and the four microbial detection‐confidence tiers (D1–D4) applied throughout this Review, with the status of each in NSCLC brain metastasis.

Grade	Definition	Minimum requirement	Status in NSCLC brain metastasis
**E1**	Direct causal evidence in an NSCLC brain‐metastasis model	Experimental perturbation of a defined microbial or microbial‐metabolite variable changes brain‐metastasis incidence or burden, with the specificity controls of Section 7 (conditional deletion, gnotobiotic reconstitution, ligand add‐back rescue)	**Not attained by any node of the axis**. No published study meets this definition.
**E2**	Mechanistic evidence obtained in another disease or model system	Causal demonstration that microbiota or microbial metabolites act on a metastasis‐relevant process (barrier integrity, glial state, immune trafficking), outside NSCLC brain metastasis	Attained for metabolite effects on the blood–brain barrier and glial state^26^, ^27^, ^28^, ^29^ and for microbiota effects on lung tumour growth and immunotherapy response.^10^, ^11^, ^12^, ^30^, ^31^
**E3**	Human association evidence	Cross‐sectional or cohort association between a microbial feature and brain‐metastasis status, treatment response or survival in a human cancer cohort; the tumour type and endpoint are named whenever they are not lung cancer and brain metastasis. Covers discrimination between existing disease states, not prospective prediction. Human work that establishes a premise rather than an association with an outcome is recorded as contextual and given a detection tier instead	Attained by three single‐centre human cohorts^20^, ^21^, ^22^ and one computational reanalysis of separately generated public datasets.^19^ Refs.^20^ and ^21^ are rated high risk of bias in the PROBAST analysis domain,^22^ moderate‐to‐high risk by QUIPS, and Ref. 19 is not formally appraisable by either tool (Table 3).
**E4**	Indirect, hypothesis‐generating extrapolation	Inference from adjacent biology with no measurement in lung cancer brain metastasis	The level at which most of the axis currently sits, including microbial carriage by disseminating tumour cells and host‐genotype modification of brain‐metastasis risk.
**D1**	Microbial nucleic acid detected	16S rRNA or ITS amplicon, shotgun metagenomics, or computational mining of bulk or single‐cell RNA‐seq, with extraction blanks, no‐template controls, batch‐matched comparators and decontam^32^ or SCRuB^33^	**The tier of every published lung cancer brain‐metastasis microbial claim** ^19^, ^20^, ^21^, ^22^ (NSCLC^19^, ^20^, ^22^; SCLC^21^). Contamination controls are incompletely reported in all four studies.
**D2**	Microbial structures localized in situ	Bacterial FISH, RNAscope, immuno‐electron microscopy, LPS or peptidoglycan immunostaining, or kingdom‐resolved spatial transcriptomics, with probe‐competition and no‐probe controls	Not attained in any NSCLC brain‐metastasis specimen. Attained in the NSCLC **primary** tumour by lipopolysaccharide and lipoteichoic acid immunostaining and imaging in the pan‐cancer survey,^34^ and at single‐cell resolution in oral and colorectal carcinoma.^35^
**D3**	Organisms viable and metabolically active	Culturomics, propidium monoazide sequencing, rRNA‐to‐rDNA ratio, metatranscriptomics or stable‐isotope probing, with heat‐killed comparators	Not attained in NSCLC at any site.
**D4**	Reproducible community confirmed by orthogonal methods	Independent cohorts, at least two orthogonal detection chemistries, within‐patient paired primary tumour and metastasis specimens, reported cross‐laboratory concordance	Not attained. This is the tier required before intratumoral microbiota can be treated as a therapeutic target.

*Note*: Evidence levels apply to mechanistic and clinical claims; detection tiers apply to claims about the physical presence of microbes and are assigned independently of evidence level. A claim may therefore be E3/D1, as all of the lung cancer brain‐metastasis microbial findings are. GRADE terminology is deliberately not used (Section 2.5).

**E1 (direct causal evidence in NSCLC brain metastasis)**. Experimental perturbation of a defined microbial or microbial‐metabolite variable changes brain‐metastasis incidence or burden in an NSCLC model, with the specificity controls set out in Section 7. **E2 (mechanistic evidence in another system)**. A causal demonstration that microbiota or microbial metabolites act on a process relevant to metastasis, such as barrier integrity, glial activation state or immune‐cell trafficking, obtained outside NSCLC brain metastasis. **E3 (human association evidence)**. A cross‐sectional or cohort association between a microbial feature and brain‐metastasis status, treatment response or survival in a human cancer cohort; this level covers discrimination between existing disease states, not prospective prediction. Where the cohort is not lung cancer, or the endpoint is not brain metastasis, the tumour type and endpoint are named with the grade, because an E3 for immunotherapy response in melanoma bears on the axis only by analogy. Human work that establishes a premise rather than an association with an outcome—that microbes are detectable in tumour tissue, for instance—is recorded as **contextual** and given a detection tier instead of an evidence level, since the E‐scheme grades claims about outcomes. **E4 (indirect, hypothesis‐generating extrapolation)**. Inference from adjacent biology with no measurement in lung cancer brain metastasis.

Two properties of the scheme should be stated so that it is not over‐read. The levels index the **setting and directness** of the evidence, not its internal validity: a large, well‐controlled human cohort and a small, poorly controlled one are both E3, and the risk‐of‐bias appraisal in Table 3 carries that information separately. E2 and E3 are also not ordered relative to each other—a claim may hold at both, and a mechanistic demonstration in another system is not automatically weaker or stronger than an association in the right disease. Only E1 is unambiguously the top of the ladder, because it alone requires the perturbation and the endpoint to be in the same system. The result of applying this scheme is shown in Figure 1b and is, in our view, the single most important output of this Review: *no node of the axis currently attains E1*. Statements in the text that are not accompanied by an explicit level should be read as E4. Two scope rules follow from the definitions. First, the levels grade claims about microbial or microbial‐metabolite variables; a purely host‐side claim, such as the role of claudin‐5 in barrier permeability, is outside the scheme and is described in plain terms instead. Second, where a level is stated for an endpoint other than brain metastasis—most often immune‐checkpoint‐inhibitor response—the tumour type and endpoint are named alongside it, because E1–E4 are anchored to the brain‐metastasis question and a level carried over from another endpoint means something different. We have deliberately not used the term GRADE anywhere in this Review. GRADE^36^ is a structured procedure for rating certainty in effect estimates across a body of comparative evidence, and the therapeutic literature reviewed here contains almost no randomized comparative data on brain‐metastasis endpoints against which such a procedure could be run. Therapeutic strategies are instead annotated with the E‐level scheme together with an explicit translational‐readiness category (TR0–TR4, defined in Table 4).

**TABLE 3:** Clinical and translational evidence linking microbiome to brain metastasis in lung cancer, with evidence level, detection tier, risk‐of‐bias appraisal and the inference each study does not support.

Study	Cohort and design	Sample / methodology	Key finding	Evidence level / detection tier	Risk‐of‐bias appraisal	What this study does NOT show	Refs
Liu et al. 2025	NSCLC patients (BM vs non‐BM); single‐centre	Faecal metagenomics + untargeted metabolomics	Distinct gut microbiome + metabolome signatures; machine‐learning classification reported **without model specification or a performance estimate**; higher α‐diversity in BM group	E3 / D1	QUIPS: high risk (study participation, confounding). PROBAST: high risk (analysis domain); no model, predictor count or performance estimate is reported, so the analysis cannot be checked	Does **not** show that microbial change precedes BM, predicts BM prospectively, or is brain‐specific rather than stage‐ or treatment‐related	^20^
Han et al. 2025	SCLC patients (BM vs non‐BM); single‐centre	Faecal metagenomics + metabolomics; machine learning	*Marvinbryantia*, benzoic acid as top discriminators of BM status; *Streptococcus* enrichment; integrated model AUC ≈ 0.80	E3 / D1	PROBAST: high risk (analysis domain; internal validation only, no calibration)	AUC is internally cross‐validated in ∼30 patients and is expected to be optimistic. Does **not** support use as a prospective predictor	^21^
Liang et al. 2025	NSCLC BM patients receiving radiotherapy; prospective	Pretreatment faecal 16S; RT response correlation	Gut microbiome composition is associated with intracranial RT response	E3 / D1	QUIPS: moderate‐high risk (single centre, confounding)	Endpoint is radiotherapy response in **established** intracranial disease. Does **not** bear on BM occurrence, prevention, or intracranial ICI response	^22^
Ma et al. 2025	NSCLC primary tumours and brain metastases drawn from **separate** public datasets; **not** within‐patient matched	Single‐cell intratumour microbiome detection (sc‐RNAseq + bacterial DNA)	Reported fungi‐dominant primary tumours → bacteria‐dominant brain mets; kingdom‐level shift	E3 / D1	Not formally appraisable (computational reanalysis); specimens not within‐patient paired	Does **not** demonstrate viable bacteria, in situ localization, carriage by tumour cells, or that the kingdom shift is biological rather than technical	^19^
Routy et al. 2018	NSCLC + RCC + UC receiving PD‐1 inhibitors	Faecal 16S; ICI response and survival outcomes	*Akkermansia muciniphila* in responders; antibiotic exposure reduces response	E3 / D1 (stool 16S)	QUIPS: low‐moderate risk	Endpoint is ICI response and survival. No CNS endpoint	^7^
Nejman et al. 2020	Pan‐cancer (> 1000 tumours, 7 cancer types incl. NSCLC)	Tumour tissue bacterial 16S profiling with LPS/LTA immunostaining and imaging, with contamination controls; no fungal (ITS) profiling	Intratumoral microbial communities detectable across all examined cancers	Contextual, not graded on E1–E4: establishes detectability rather than an association with an outcome. Detection tier D1–D2	Extensive negative controls reported	Establishes detectability across tumour types. Does **not** address brain metastasis or causality	^34^
Galeano‐Niño et al. 2022	Oral squamous cell carcinoma (OSCC) and colorectal cancer (CRC)	Single‐cell intratumour + spatial bacterial detection	Bacterial communities populate immunosuppressive, poorly vascularized microniches; associated with low‐Ki67 malignant cells; promote spatial heterogeneity in TME	E2 / D2	Well‐controlled; FISH and spatial confirmation	Obtained in oral and colorectal carcinoma. Extrapolation to NSCLC brain metastasis is inference	^35^
Tsay et al. 2018	Lung cancer patients; lower‐airway brushings and BAL, cross‐sectional	Lower‐airway 16S rRNA with paired airway‐epithelial transcriptomics	Lower‐airway enrichment of oral commensals (*Streptococcus*, *Veillonella*) is associated with upregulation of PI3K pathway signalling in the airway epithelium	E3 / D1	QUIPS: moderate risk	No brain‐metastasis endpoint. Cross‐sectional airway sampling; does **not** demonstrate translocation of oral taxa into tumour tissue	^18^
Derosa et al. 2018	NSCLC + RCC on PD‐1 inhibitors	Antibiotic exposure ascertainment; clinical outcomes	Antibiotic exposure within 30–60 days of ICI reduces response and survival	E3 / not applicable	ROBINS‐I: serious risk (confounding by indication)	Antibiotic exposure is confounded by infection and performance status. No BM endpoint	^37^
Gopalakrishnan et al. 2018; Matson et al. 2018	Melanoma cohorts on PD‐1 inhibitors	Faecal 16S; ICI response correlation	*Faecalibacterium*, *Bifidobacterium* associated with ICI response	E3 (melanoma; ICI‐response endpoint); stool 16S, detection tier D1	QUIPS: low‐moderate risk	Melanoma cohorts; no CNS or lung endpoint	^8^, ^9^

*Note*: Evidence levels and detection tiers as defined in Sections 2.5 and 2.6. Two conventions govern this column and are stated here because they are not self‐evident from the table. First, a level is assigned to what the study contributes to the brain‐metastasis question, not to the study's internal quality: the melanoma cohorts are therefore left ungraded and labeled contextual, because E3 is defined for human lung cancer, whereas pan‐cancer and other‐tumour‐type work that establishes a mechanism or a detection method is graded on that contribution. Second, detection tier is assigned wherever the study detects microbes by any method, including stool sequencing, and is marked not applicable only where no microbial detection was performed, as in the antibiotic‐exposure cohort. Risk of bias appraised with QUIPS^38^ for prognostic‐factor studies (participation, measurement and confounding domains only, since the cohorts are cross‐sectional), PROBAST^39^ for prediction‐model studies and ROBINS‐I^40^ for non‐randomized intervention studies; the computational reanalysis is not appraisable with any of these and is assessed against the provenance criteria of Table 5 instead. The final column is included because misreading of study scope, rather than misreading of study results, is the dominant source of overstatement in this field.

Abbreviations: BM, brain metastasis; ICI, immune checkpoint inhibitor; RT, radiotherapy; RCC, renal cell carcinoma; TME, tumour microenvironment; UC, urothelial carcinoma.

**TABLE 4:** Therapeutic strategies targeting the microbiome–brain metastasis axis in NSCLC, organized by axis node and clinical readiness.

Intervention	Axis node targeted	Mechanism of action	Strongest evidence	Clinical readiness	Evidence level (BM endpoint / best‐supported endpoint) and translational readiness	Refs
Probiotic adjuvants (*Akkermansia*, *Bifidobacterium*, *Faecalibacterium*)	Node 1 (gut microbiome)	Restore gut diversity; enhance T cell priming; butyrate restoration	Multiple preclinical models; early‐phase NSCLC trials	Phase II in NSCLC + ICI	E4 for BM; E2–E3 for ICI response. TR1 (BM) / TR3 (ICI)	^7^
Faecal microbiota transplantation (FMT)	Node 1 (gut microbiome)	Complete community transfer from healthy or ICI‐responder donors	Single‐arm phase I/II responder‐donor FMT in anti‐PD‐1‐refractory melanoma^41^, ^42^; NSCLC pilots	Phase I‐II in NSCLC; licensed live biotherapeutic products exist for recurrent *C. difficile*, conventional FMT under enforcement discretion	E4 for BM; E3 for ICI‐refractory melanoma. TR0 (BM) / TR2 (cancer)	^41^, ^42^
Antibiotic stewardship	Node 1 (gut microbiome preservation)	Minimize antibiotic‐induced dysbiosis around ICI	Strong observational data linking antibiotics to worse ICI response	Implementable now in oncology practice	E4 for BM; E3 for ICI outcomes in NSCLC. TR1 (BM) / TR4 (stewardship as practice)	^37^
High‐fibre / Mediterranean diet	Nodes 1, 3 (microbiome + metabolites)	Promote butyrate‐producer expansion; SCFA generation	Cohort studies; pilot trials in melanoma	Implementable; adherence challenges in oncology	E4 for BM; E3 for diet and ICI response. TR1 / TR2	^43^
SCFA precursor supplementation (butyrylated starch, inulin)	Node 3 (metabolites + BBB)	Restore butyrate; protect BBB integrity	Mouse colonic Treg induction^44^, ^45^; human gut‐health trials of fermentable fiber. No oncology trial with a barrier or brain‐metastasis endpoint	Phase 0–I in non‐oncology indications	E4 for BM; E2 for barrier effects in rodents. TR1	^44^, ^45^
MR1/MAIT ligand modulation (exogenous 5‐OP‐RU)	Node 4 (MR1/MAIT axis)	Activate MAIT cells; the intended cytotoxic program requires a concurrent IL‐12/IL‐15/IL‐18 signal (Section 8.5)	Preclinical experimental autoimmune encephalomyelitis^46^; the only published tumour study reports that MAIT cells **promote** tumour growth and experimental lung metastasis through tumour MR1, with *Mr1* ^−/−^ animals protected,^47^ i.e. the opposite sign to the hypothesis this row rests on	Early discovery; first‐in‐human pending	E4 for BM; E2 for MAIT biology. TR1. See Section 8.5 for immunopathology liabilities. **The direction of effect is unsettled** ^47^ **; MR1 blockade is as plausible a therapeutic hypothesis as MR1 agonism**	^46^, ^47^
Intracellular antibiotic targeting (intratumoural bacteria)	Node 2 (intratumoral microbiome)	Disrupt intracellular bacteria in tumour cells	Conceptual; preclinical proof‐of‐principle	Discovery stage	E4. Rests on D1 detection only; not justifiable at present. TR0	^35^
BBB stabilization (claudin modulators, S1P agonists)	Node 3 (BBB integrity)	Strengthen tight junctions; prevent metastatic extravasation	Preclinical; some approved for other indications	Discovery (cancer indication)	E4 for BM; Claudin‐5 is an established host determinant of barrier permeability,^48^, ^49^ which sits outside the E1–E4 scheme (Section 2.5). TR0–TR1	^48^, ^49^
Integrated axis‐stratified intervention	Multiple nodes	Combined modulation based on patient profile	Conceptual framework; basis for stratified trials	Trial design phase	E4. Design proposal only (Figure 8). TR0	No primary evidence; design proposal only (Figure 8)
ICI + microbiome adjuvant combination	Nodes 1, 4 (gut + immune)	Restore microbiome‐immune coordination; enhance ICI efficacy	Multiple NSCLC trials ongoing	Phase II ongoing	E4 for intracranial endpoints; E3 for systemic ICI response. TR1 / TR3	^7^

Coming: Evidence levels are as defined in Section 2.5 and are stated separately for the brain‐metastasis endpoint and for the endpoint on which the intervention is best supported, because these differ for every entry. Translational‐readiness categories: **TR0**, target hypothesis only; **TR1**, preclinical efficacy in a non‐NSCLC or non‐brain‐metastasis system; **TR2**, pilot clinical data in cancer for a different endpoint; **TR3**, controlled clinical evidence for a different endpoint; **TR4**, established practice for a different endpoint. No intervention reaches TR2 or above for a brain‐metastasis endpoint. The “Clinical readiness” column reports the most advanced trial stage reached for any indication: Discovery (target validation only); Preclinical (animal‐model efficacy); Phase 0–I, Phase I–II and Phase II (as registered); and Implementable (established clinical practice for another indication). That column therefore describes trial stage in any disease, whereas the TR category describes readiness for the brain‐metastasis endpoint specifically; the two differ for every row. A GRADE column is not used, because no GRADE assessment was performed and the comparative evidence required to perform one does not exist (Section 2.5). Mechanism descriptions reflect the most parsimonious axis‐relevant interpretation, not the full pharmacology of each intervention.

### 2.6 Detection‐confidence hierarchy for microbial signals

Claims about the presence of microbes in tumours, in metastases or in the brain require a second, orthogonal grading, because detection of a microbial sequence is not the same as demonstration of a microbial community. Low‐biomass specimens are vulnerable to reagent and laboratory contamination,^50^, ^51^ to index hopping and to database misclassification, and the consequences of ignoring this are not hypothetical: a prominent pan‐cancer machine‐learning analysis of tumour‐associated microbial reads^15^ was retracted after independent reanalysis showed that its findings were driven by data‐processing artifact and contamination.^14^ We therefore grade every detection claim on the following four tiers (Table 2, Figure 1c).

**D1, microbial nucleic acid detected**. 16S rRNA or ITS amplicon sequencing, shotgun metagenomics, or computational mining of bulk or single‐cell RNA sequencing. Minimum acceptable controls are extraction blanks, no‐template amplification controls, batch‐matched processing of comparator groups, and statistical decontamination with decontam^32^ or SCRuB.^33^ **D2, microbial structures localized in situ**. Bacterial fluorescence in situ hybridization, RNAscope, immuno‐electron microscopy, lipopolysaccharide or peptidoglycan immunostaining, or kingdom‐resolved spatial transcriptomics, with probe‐competition and no‐probe controls. **D3, organisms viable and metabolically active**. Culturomics, propidium monoazide sequencing, rRNA‐to‐rDNA ratio, metatranscriptomics or stable‐isotope probing, with heat‐killed comparators. **D4, reproducible community confirmed orthogonally**. Independent cohorts, at least two orthogonal detection chemistries, within‐patient paired primary tumour and metastasis specimens, and reported cross‐laboratory concordance.

Applied to the present literature, the outcome is unambiguous and is stated here so that it need not be repeated at every occurrence: *every microbial claim in NSCLC brain metastasis published to date rests on D1*. No study has reached D2 in a brain metastasis specimen, no study has demonstrated viable organisms (D3), and no finding has been replicated across independent cohorts with orthogonal chemistry (D4). Statements about intratumoral or intracerebral bacteria in this Review should be read accordingly.

### 2.7 Terminology

Microbial signals, bacterial elements, intratumoral bacteria, microbiota and microbiome are not interchangeable, and this Review uses them as follows. *Microbial nucleic acid* or *microbial reads* denotes sequence detected in a specimen, with no implication as to the physical state of the organism. *Microbial structures* denote morphologically or antigenically identified bacterial or fungal material localized in tissue. *Viable bacteria* denote organisms shown to be alive or metabolically active. *Intratumoral microbiota* denotes a community of organisms whose presence has been established at D3 or above. *Microbiome* is reserved for the community together with its genomes and metabolic output. Throughout, the presence of bacteria is stated at the level actually measured rather than assumed from sequence detection alone.

### 2.8 Risk‐of‐bias appraisal

Human observational studies of microbial features and brain‐metastasis status were appraised with QUIPS,^38^ with particular attention to the study‐participation and study‐confounding domains, because comparator‐group definition and unmeasured exposure to antibiotics, corticosteroids and proton pump inhibitors are the dominant threats to validity in this literature. QUIPS is designed for prognostic factor studies with follow‐up; the cohorts appraised here are cross‐sectional, so its attrition and outcome‐measurement domains are not applicable and were recorded as such rather than scored, and the appraisal should be read as covering participation, measurement and confounding only. Prediction‐model studies were appraised with PROBAST.^39^ The classifier for which performance is reported is that of the SCLC cohort,^21^ an integrated microbiome–metabolite model with a cross‐validated AUC of approximately 0.80 in roughly 30 patients; it was rated high risk of bias in the analysis domain on the grounds of sample size relative to the candidate predictor space, internal‐only validation and absent calibration reporting. The NSCLC cohort^20^ reports machine‐learning discrimination between groups without giving the model, the number of candidate predictors or a performance estimate, so the same analysis‐domain concerns apply and cannot be checked; we record it as high risk of bias for that reason and say so rather than implying that a reported model was appraised. Non‐randomized intervention studies were appraised with ROBINS‐I.^40^ Animal studies underpinning E2 claims were read against the ARRIVE 2.0 essential items,^52^ with randomization, blinded outcome assessment and sample‐size justification noted. ARRIVE is a reporting checklist rather than a risk‐of‐bias instrument, and we use it only to record what was reported, not to assign a bias rating. Two further study types occur in this literature and are appraised outside these tools: computational reanalyses of public sequencing data, which no validated instrument covers and which we appraise instead against the detection‐confidence criteria of Section 2.6 and the provenance criteria of Table 5 (specimen pairing, batch structure, availability of extraction blanks, contaminant‐filtering method); and mechanistic in vitro studies, for which we record whether the effect was demonstrated with a genetic as well as a pharmacological perturbation and whether a dose–response was established. Because no animal study addresses an NSCLC brain‐metastasis endpoint, animal studies were not assigned a row in Table 3, which reports per‐study appraisal for the human and computational studies only.

**TABLE 5:** Public data resources underlying substantive claims in this Review.

Resource and claim supported	Repository / accession	Platform	Specimens and patients	Within‐patient pairing	Quality control reported by original authors	Limitation this imposes
Single‐cell intratumour microbiome of NSCLC primary tumours and brain metastases; source of the reported lung‐to‐brain kingdom shift^19^	Constituent single‐cell datasets are those listed by the original authors^19^; no consolidated accession is issued for the aggregate. Reanalysis pipeline SAHMI^53^: github.com/sjdlabgroup/SAHMI, Zenodo https://doi.org/10.5281/zenodo.7017103	10x Genomics scRNA‐seq, reanalysed for microbial reads	Primary tumour and brain metastasis libraries drawn from separate published studies	**No**. Primary and metastatic specimens are from different patients, laboratories and sequencing batches	SAHMI contaminant filtering and k‐mer denoising; extraction blanks not available retrospectively	The kingdom‐level shift cannot be distinguished from differential technical and contamination background between the two specimen sets. Detection tier D1. Treated throughout as hypothesis.
Gut metagenomics and untargeted metabolomics, NSCLC with versus without brain metastasis^20^	**Not publicly deposited**. The data availability statement of Ref. 20 states that the data are available from the corresponding author on reasonable request	Shotgun metagenomics; LC‐MS/MS metabolomics	*n* = 18 brain metastasis, *n* = 12 no distant metastasis; 30 patients	Not applicable (single time point, single compartment)	Standard quality filtering; antibiotic, corticosteroid and PPI exposure not fully stratified	Single centre, single group, cross‐sectional. Supports association with brain‐metastasis status only; PROBAST high risk of bias in the analysis domain.
Faecal metagenomics and metabolomics, SCLC with versus without brain metastasis^21^	Science Data Bank, DOI 10.57760/sciencedb.28513 (“Faecal metagenomic and metabolomic sequencing results”). The data availability statement of^21^ prints the prefix as 10.57760/scientificdb.28513, which does not resolve; the identifier given here is the one that does	Shotgun metagenomics; metabolomics; machine‐learning classifier	Approximately 30 patients	Not applicable	Internal cross‐validation only; no external validation cohort; calibration not reported	Reported AUC is an internally validated estimate and is expected to be optimistic. Taxa do not overlap with the NSCLC cohort^20^.
Pretreatment gut 16S and radiotherapy response in established NSCLC brain metastasis^22^	**Not publicly available**.^22^ states that access is restricted for patient privacy and must be requested from the corresponding author	16S rRNA amplicon sequencing	Single‐centre cohort of patients with existing brain metastases	Not applicable	Not fully reported	Endpoint is radiotherapy response in established disease. **Must not be cited as evidence about brain‐metastasis occurrence, prevention, or intracranial immunotherapy response**.
Pan‐cancer tumour microbiome reference, including NSCLC^34^	NCBI BioProject **PRJNA624822** (“The human tumour microbiome”, 1526 tumours across seven cancer types), plus the published supplementary tables of Ref. 34	Bacterial 16S rRNA profiling with LPS and LTA immunostaining and imaging; the pan‐cancer fungal survey is a separate study^54^	>1000 tumours across 7 cancer types	Not applicable	Extensive negative controls and contaminant‐aware analysis reported	Establishes detectability (D1–D2) across tumour types; does not address brain metastasis or causality.
Pan‐cancer blood and tissue microbial machine‐learning classifier^15^	Derived from The Cancer Genome Atlas whole‐genome and RNA‐sequencing read sets; no separate deposition. **The article has been retracted**	Reanalysis of TCGA whole‐genome and RNA sequencing	Pan‐cancer	Not applicable	Subsequently shown to be inadequate^14^	**Retracted in 2024**. Cited in this Review only as a cautionary example of contamination and analytic artifact in low‐biomass work, never as supporting evidence.

*Note*: Public data resources underlying substantive claims in this Review, with provenance, quality‐control information and the limitation each imposes. The repository column gives the identifier reported by the original authors where one exists, and states explicitly where the data have not been deposited.

Abbreviation: scRNA‐seq, single‐cell RNA sequencing.

### 2.9 Public data resources and provenance

Several of the findings central to this Review are derived not from newly generated specimens but from reanalysis of public sequencing data, and the properties of those datasets materially constrain what can be concluded. Table 5 therefore lists, for every public resource cited in support of a substantive claim, the repository and accession, the platform, the number of specimens and patients, whether primary tumour and brain metastasis specimens were obtained from the same individuals, the contamination controls reported, and the quality‐control steps applied by the original authors. The single most consequential entry is the dataset underlying the reported lung‐to‐brain kingdom shift^19^: the primary‐tumour and brain‐metastasis single‐cell libraries were generated in separate studies, in different laboratories and sequencing batches, and were aggregated computationally. They are not within‐patient paired specimens, a point stated explicitly in Sections 4.5, 5.8 and 6.5 and in Tables 3 and 6. A second entry deserves comment: of the two lung‐cancer cohorts that carry most of the clinical weight of this Review, only the SCLC dataset^21^ is publicly deposited (Science Data Bank, DOI 10.57760/sciencedb.28513); the NSCLC brain‐metastasis dataset^20^ is available only from its corresponding author on request, and the radiotherapy‐response dataset^22^ is access‐restricted for patient privacy. Independent reanalysis of the two datasets on which the NSCLC‐specific claims rest is therefore not currently possible, which is itself a reason to treat those claims as hypothesis‐generating. No new datasets were generated for this Review; all analyses reported here are of previously published data.

**TABLE 6:** Falsifiable predictions of the microbiome–brain metastasis axis and critical experiments for testing them.

Prediction	Axis node tested	Critical experiment	Decisive readout	Status
Gut microbiome composition predicts brain metastasis occurrence **prospectively** in NSCLC (**untested: all existing data are cross‐sectional**)	Node 1	Prospective cohort with serial sampling and 24‐month BM ascertainment	ΔAUC ≥ 0.05 over a base model containing Lung‐molGPA components and driver genotype, whose 95% lower confidence bound exceeds 0.05, with positive decision‐curve net benefit,^55^ acceptable calibration and external validation (Section 6.6.2)	**Untested**. Existing data^20^, ^21^ are cross‐sectional associations with BM **status** (E3), not prospective prediction; both from a single centre and the same group, PROBAST high risk of bias
Intratumoral bacteria within primary tumours accompany tumour cells to brain (**hypothesis, not observation**)	Node 2	Within‐patient paired primary tumour and brain metastasis from the same patients, profiled with extraction blanks and in situ confirmation; the mouse counterpart is a paired‐site experiment in an NSCLC model with defined mono‐colonization	Within‐patient paired primary tumour and brain metastasis with extraction blanks, plus in situ confirmation by bacterial FISH or RNAscope (detection tier D2); mouse perturbation as the follow‐on test (Section 7.6)	E4. The single supporting analysis^19^ compared **separately generated, non‐paired** public datasets; a technical origin for the kingdom shift is not excluded. Orthogonal replication and within‐patient pairing pending
Microbiome‐modulating intervention reduces BM incidence in high‐risk NSCLC	Node 1	RCT of probiotic/FMT adjuvant to ICI in EGFR‐mutant or ALK+ NSCLC	Reduced 24‐month BM incidence vs. control	Untested
Butyrate‐producer restoration improves BBB integrity	Node 3	Pre/post intervention BBB permeability imaging (DCE‐MRI, contrast extravasation)	Increased TJ markers; reduced permeability metrics	Untested
Antibiotic exposure increases brain metastasis in mouse NSCLC	Node 1	Antibiotic + KRAS‐G12D mouse model; intracardiac/intracarotid seeding	Increased BM frequency with antibiotic exposure	Untested
MAIT cells modify brain metastatic colonization (direction unresolved)	Node 4	*Mr1* ^−/−^ **and** *Traj33* ^−/−^ versus wild‐type mice bearing an NSCLC line; the pair separates the presenting molecule from the MAIT cell (Section 7.2, Table 8)	A difference in either direction: increased burden in *Mr1* ^−/−^ would support MAIT surveillance; decreased burden would replicate the tumour‐promoting role reported^47^	Untested in brain metastasis. In subcutaneous and experimental lung‐metastasis models, *Mr1* ^−/−^ mice were protected^47^ (E2), which is the opposite of the surveillance hypothesis
Plasma kynurenine pathway activation predicts BM in NSCLC	Node 3	Prospective NSCLC serum metabolomics + BM ascertainment	ΔAUC ≥ 0.05 over the same base model, with positive decision‐curve net benefit^55^ and acceptable calibration in an independent cohort	E3 for a cross‐sectional association^20^; **untested** as a prospective predictor. Microbial versus host IDO1 origin not distinguished
Lung‐to‐brain bacterial selection is driven by intracellular persistence	Node 2	Compare intracellular vs extracellular tumour bacteria in dissemination assays	Brain metastases selectively retain intracellular bacterial communities	Untested
Gut microbiome predicts intracranial ICI response (independently of extracranial)	Nodes 1, 4	NSCLC BM cohort with intracranial response measurement + gut microbiome	ΔAUC ≥ 0.05 over extracranial response plus clinical covariates, in a cohort with protocol‐defined intracranial response assessment	**Untested (E4)**. The radiotherapy‐response cohort^22^ addresses treatment response in established intracranial disease and does not bear on intracranial immunotherapy response (Section 2.10)
CARD9/CLEC7A variants modify microbiome‐BM risk relationship	Host genetics × Node 1	Stratified cohort: genotype × microbiome × BM incidence	Significant gene × microbiome interaction	Untested

*Note*: Status is stated using the evidence levels of Section 2.5. Evidence levels in this table grade the **prediction**, not the study that motivated it: the single‐cell analysis^19^ is graded E3/D1 in Table 3 as a human observation, while the carriage hypothesis it is used to motivate is graded E4 here, because no study has tested it. Prediction 9 is labelled untested (Section 2.10 and Section 6.4): the radiotherapy‐response cohort^22^ addresses treatment response in established intracranial disease and does not bear on intracranial immunotherapy response.

Abbreviations: AUC, area under the receiver operating characteristic curve; BM, brain metastasis; DCE‐MRI, dynamic contrast‐enhanced magnetic resonance imaging; ICI, immune checkpoint inhibitor; TJ, tight junction; WT, wild type.

### 2.10 Citation audit

Every citation supporting a mechanistic statement, clinical interpretation or therapeutic recommendation was re‐checked against the original article by two authors independently. The radiotherapy‐response cohort^22^ addresses treatment response in established intracranial disease; it is not used elsewhere in this Review to support a prediction about intracranial immunotherapy response or brain‐metastasis prevention, and that prediction is labelled untested (Table 6). The single‐cell intratumour microbiome analysis^19^ is a computational reanalysis of separately generated public datasets rather than paired within‐patient specimens (Section 2.9). Statements about metabolite function are supported either by direct metabolomic measurements or are labelled as inference (Section 4.4 and Table 7). Table 7 cites the primary transport and systemic‐lipopolysaccharide studies^56^, ^57^ rather than narrative reviews. One pan‐cancer resource relevant to this literature, since retracted,^14^, ^15^ is cited only as a cautionary example (Section 5.8). Claims that rest on indirect inference are identified as such in the text and are not supported by references addressing only an adjacent biological process.

**TABLE 7:** Directness of the evidence supporting each metabolite–mechanism pair in the proposed axis.

Metabolite and proposed mechanism	Primary evidence source	Disease / model system in which it was shown	Directness for NSCLC BM	Was the metabolite measured, or inferred from taxonomy?	Validation status	Evidence level
Butyrate strengthens blood–brain barrier tight junctions	Germ‐free and mono‐colonized mice; sodium butyrate administration^26^	Germ‐free mouse; no tumour	Extrapolated	Measured in the mechanistic study; in lung cancer cohorts^20^, ^21^ inferred from producer‐taxon abundance	Reproduced in independent germ‐free work; never tested in a cancer model	E2
Butyrate and propionate signal through FFAR2 and FFAR3; butyrate, but not propionate, also engages HCAR2	*Ffar2* ^−/−^ mice^58^; *Hcar2* ^−/−^ mice, butyrate‐specific^59^; *Ffar3* ^−/−^ mice^60^	Colitis and colon carcinogenesis^58^, ^59^; host energy metabolism^60^	Extrapolated	Measured	Genetically validated receptors; not tested for a brain endpoint	E2
Butyrate‐producer depletion indicates reduced butyrate availability in BM patients	Machine‐learning feature ranking in SCLC^21^; SCFA‐related metabolite shifts in NSCLC^20^	Human lung cancer, cross‐sectional	Indirect	**Inferred from taxonomy in Ref**. 21; **partially measured by untargeted metabolomics in Ref**. 20] **without targeted SCFA quantification**	Not replicated; taxa non‐overlapping between the two cohorts	E3 for the association; E4 for the functional inference
Microbial indoles engage AhR on astrocytes and microglia to limit CNS inflammation	Astrocyte‐ and microglia‐restricted *Ahr* deletion; dietary tryptophan manipulation^27^, ^28^	Experimental autoimmune encephalomyelitis	Extrapolated	Measured	Strongly validated with conditional genetics; no tumour or metastasis endpoint	E2
Kynurenine crosses the BBB via LAT1 and shapes neuroactive metabolite pools	Direct BBB transport measurement of kynurenine^56^; pathway reviews^61^, ^62^ for context	Rodent and in vitro	Extrapolated	Measured	Established transport biology	E2
Elevated kynurenine‐to‐tryptophan ratio in NSCLC BM reflects microbial tryptophan metabolism	Altered tryptophan‐derivative profile in NSCLC BM patients^20^	Human NSCLC, cross‐sectional	Indirect	Measured (untargeted); **microbial versus host IDO1 origin not distinguished**	Not replicated; host IDO1 induction is an equally parsimonious explanation	E3 for the association; E4 for the microbial attribution
Secondary bile acids modulate Treg/Th17 balance (3‐oxoLCA inhibits RORγt; isoalloLCA promotes Treg via the FOXP3 CNS3 enhancer)	Bile‐acid metabolite administration with direct RORγt binding and FOXP3 enhancer readout^63^; gut RORγ+ Treg homeostasis^64^; enterohepatic reviews^65^ for context	Mouse colon and systemic immunity	Extrapolated	Measured	Well replicated outside oncology	E2
Circulating LPS primes microglia through TLR4 toward a metastasis‐permissive state	Systemic LPS challenge with microglial and neurodegenerative readout in mice^57^; PAMP‐receptor reviews^66^, ^67^ for context	Rodent neuroinflammation	Extrapolated	**Not measured in any lung cancer BM cohort; no study has reported circulating LPS or LBP**	Untested in cancer	E4
Benzoic acid discriminates BM status in SCLC	Machine‐learning feature in a single cohort^21^	Human SCLC, cross‐sectional	Indirect	Measured	Single centre, internal validation only; CNS mechanism unknown	E3
Acetate is the most abundant circulating SCFA and modulates microglial and endothelial function	Germ‐free and SCFA‐supplementation studies in mice^26^; receptor assignment from *Ffar2* ^−/−^ mice^58^	Germ‐free mouse; no tumour	Extrapolated	Measured in the mechanistic studies; **not measured in any lung cancer cohort**	Reproduced outside oncology; no brain‐metastasis endpoint	E2 for the mechanism; E4 for any role in NSCLC BM
Indoxyl sulphate, a host‐sulphated bacterial indole, is a uremic solute with endothelial effects	No primary source is cited for a barrier or brain‐metastasis effect	Not applicable	Extrapolated	**Not measured in any lung cancer brain‐metastasis cohort**	Untested in this context; included in Table 10 for completeness of the metabolite inventory	E4
Peptidoglycan engages NOD2 and contributes to innate priming	Reviews of pattern‐recognition receptor biology^66^, ^67^; **no primary peptidoglycan study is cited here**	Not applicable	Extrapolated	**Not measured in any lung cancer brain‐metastasis cohort**	Untested in cancer; listed because Table 10 includes it	E4

*Note*: Directness is classified as Direct (causal or mechanistic evidence obtained in NSCLC brain metastasis), Indirect (human association evidence in lung cancer, including cross‐sectional brain‐metastasis cohorts, without mechanistic demonstration) or Extrapolated (obtained outside lung cancer). No row in this table is Direct. Measurement states whether the metabolite itself was quantified in the cited lung cancer cohort or whether its abundance was inferred from the relative abundance of producing taxa.

Abbreviations: BBB, blood–brain barrier; EAE, experimental autoimmune encephalomyelitis.

## 3 HOST GENETIC ARCHITECTURE OF TUMOUR–MICROBIOME INTERACTION IN NSCLC

### 3.1 Host genetics shapes both microbiome composition and cancer susceptibility

Gut and lung microbial community composition is shaped by host genetics in addition to environment, diet, and pharmacological history. Twin studies and population‐scale analyses document reproducible heritability for a minority of gut taxa, notably *Bifidobacterium* and *Christensenellaceae*,^68^, ^69^ although the largest population study concluded that environmental factors dominate over host genetics and detected no significant heritability for most taxa^70^; lung microbiome heritability is less characterized but is influenced by host mucin and immune genes. Where the same host variants modulate both microbiome composition and cancer‐relevant immunity, gene‐microbe interactions become biologically plausible candidates for risk stratification. In NSCLC, driver mutations (EGFR, KRAS, ALK, TP53) shape both tumour biology and the local lung microbiome, while polymorphisms in pattern recognition receptors (CARD9, CLEC7A, TLR4) modulate both gut microbiome composition and anti‐tumour immunity, providing a substrate by which host germline variation could constrain the microbiome–brain metastasis axis. We note at the outset that no host variant has been shown to modify brain‐metastasis risk through a microbiome‐dependent mechanism in any cohort; this section is E4 throughout unless otherwise stated.

### 3.2 Driver mutations of NSCLC and their interactions with the microbiome

NSCLC driver mutations co‐define tumour biology and the host immune‐microbiome environment. EGFR‐mutant adenocarcinomas are proposed to carry distinct lung tumour microbiome profiles (altered diversity, *Streptococcus* enrichment), although the published lung tumour microbiome comparisons are stratified by histology and TP53 status rather than by EGFR genotype,^18^, ^71^ so this remains untested (E4), and show the highest brain metastasis rates among NSCLC subtypes (40%–60% over disease course),^4^, ^5^ with high TKI sensitivity^72^, ^73^ but generally poor ICI monotherapy response. KRAS‐mutant disease shows distinct intratumoral microbial signatures and immune infiltrate with implications for ICI response. ALK‐fusion NSCLC, defined by the EML4‐ALK rearrangement^74^ and common in younger never‐smokers, also carries elevated brain metastasis risk (50%–70%).^4^ TP53 alterations (≈50% of NSCLC) modulate tumour‐microbiome inflammatory crosstalk through p53‐dependent cytokine regulation, and whole‐exome analysis of resected lung adenocarcinoma brain metastases shows that the intracranial lesion is genomically divergent from the matched primary, with MYC, YAP1 and MMP13 amplifications significantly enriched in the metastasis.^75^ Whether driver mutation status mechanistically influences microbiome‐mediated brain metastasis risk, or merely correlates with it through shared upstream factors (smoking, age), is unresolved; driver‐stratified prospective cohorts pairing tumour and gut metagenomics with brain metastasis outcomes are required.

### 3.3 Pattern‐recognition receptors and the Dectin–CARD9 axis in tumour immunity

Pattern recognition receptors (PRRs) link microbial detection to host immunity with direct implications for both microbiome composition and anti‐tumour responses. The Dectin–CARD9 axis is paradigmatic: Dectin‐1 (CLEC7A) recognizes fungal β‐glucan and signals through Syk to the adaptor CARD9, activating NF‐κB and driving IL‐17/IL‐22 production.^76^ Related C‐type lectin receptors, Dectin‐2 and Dectin‐3, recognize fungal mannans with overlapping antifungal functions.^77^, ^78^ The same axis has been implicated in anti‐tumour immunity, although the direct tumour evidence lies outside lung cancer: Dectin‐1 ligation on tumour‐associated myeloid cells shapes the immune infiltrate in pancreatic cancer^79^ (E2), while Refs. 80 and 81 describe the antifungal and colitis biology of the pathway rather than a tumour phenotype; CARD9 deficiency alters microbial tryptophan metabolism and reduces AhR ligand production in mice and in patients with inflammatory bowel disease, which is the only one of these links for which we can find primary support; we are not aware of any study relating CARD9 genotype to tumour microbiome composition or to immune‐checkpoint‐inhibitor response, and we state both as untested (E4). The Dectin–CARD9 axis thus provides a candidate genetic substrate for identifying NSCLC subgroups in which microbiome‐targeted therapy combined with ICI may be disproportionately effective, a proposal that is untested (E4) and for which the relevant knockout experiments are specified in Section 7 and Table 8.

**TABLE 8:** Gene‐knockout and genetic‐perturbation program for establishing the specificity of each node of the axis.

Gene / target	Axis node	System and perturbation	Predicted phenotype if the node is real	Specificity controls that make the result interpretable	Decisive readout	Current status
*Mr1*	Node 4	*Mr1* ^−/−^ mouse (no MAIT cells) bearing an NSCLC line; intracardiac or intracarotid seeding	Increased cerebral colonization and burden if MAIT surveillance is protective; **the opposite is equally possible** — *Mr1* ^−/−^ mice showed reduced tumour growth and experimental lung metastasis in the one published tumour study^47^, so the experiment is read two‐sided	Pair with *Traj33* ^−/−^ (MAIT‐deficient, MR1‐intact) to separate the presenting molecule from the cell; littermate controls; co‐housing to test microbiota transmissibility; pre‐specify a two‐sided hypothesis given the contrary published direction^47^	BM lesion number and total burden; brain MAIT frequency and phenotype	Not performed
*Traj33* (Jα33‐deficient)	Node 4	*Traj33* ^−/−^ mouse; same seeding model	Phenocopy of *Mr1* ^−/−^ if the effect is MAIT‐intrinsic	Direct comparison with *Mr1* ^−/−^; adoptive MAIT transfer rescue	BM burden; rescue by MAIT transfer	Not performed
*Card9*	Nodes 4 and 5	*Card9* ^−/−^ mouse; NSCLC seeding model	Altered BM burden with altered myeloid and IL‐17 tone	**Mandatory**: *Card9* ^−/−^ animals have divergent baseline mycobiota and microbiota, so littermate controls, co‐housing and germ‐free rederivation are required before any effect is called cell‐intrinsic	BM burden; intratumoral and brain myeloid profiling; faecal mycobiome	Not performed
*Clec7a* (Dectin‐1)	Node 4	*Clec7a* ^−/−^ mouse; β‐glucan challenge; NSCLC seeding	Altered fungal sensing and altered anti‐tumour myeloid activation	As for *Card9*; plus β‐glucan add‐back	BM burden; CD8 priming; myeloid activation state	Not performed
*Ffar2* / *Ffar3*	Node 3	Double knockout; NSCLC seeding under defined‐fiber diet	Loss of the protective effect of SCFA supplementation on barrier integrity and colonization	Diet‐matched controls; butyrate add‐back must fail to rescue in the knockout and succeed in wild type	BBB tracer extravasation; claudin‐5; BM burden	Not performed
*Hcar2* (Gpr109a)	Node 3	*Hcar2* ^−/−^ mouse; butyrate or niacin supplementation	Abolition of butyrate‐dependent barrier and myeloid effects	Ligand dose–response; receptor re‐expression rescue	BBB permeability; microglial transcriptome	Not performed
*Ahr* (conditional)	Node 3	*Ahr* ^fl/fl^;*Gfap*‐Cre (astrocyte) and *Ahr* ^fl/fl^;*Cx3cr1*‐CreERT2 (microglia); NSCLC seeding	Cell‐type‐specific change in cerebral colonization if glial AhR sensing of microbial indoles is on the path	Tamoxifen‐treated Cre‐negative littermates; dietary tryptophan manipulation; indole add‐back	BM burden; astrocyte and microglial single‐cell transcriptomes	Not performed
*Cldn5* (conditional)	Node 3	*Cldn5* ^fl/fl^;*Cdh5*‐CreERT2 endothelial deletion after tumour establishment	Increased cerebral colonization if barrier integrity is rate‐limiting; **no change would falsify the barrier arm of the axis**	Tamoxifen‐treated Cre‐negative littermates; two tracer sizes to confirm size‐selective opening	Tracer extravasation; BM lesion number; intravital imaging of extravasation	Not performed
*Tlr4* / *Nod2*	Node 3	Single knockouts; systemic LPS or peptidoglycan challenge; NSCLC seeding	Loss of PAMP‐driven microglial priming and of any associated increase in colonization	Heat‐inactivated and ultrapure ligand controls; germ‐free comparison	Microglial state; BM burden	Not performed
Tumour‐cell‐intrinsic candidates (CRISPR)	Node 2	Knockout in the tumour line of genes required for bacterial internalization or intracellular persistence; brain‐seeding assay	Reduced cerebral colonization of bacteria‐bearing clones without change in extracranial seeding	Two independent sgRNAs; non‐targeting guide; **rescue by guide‐resistant cDNA re‐expression**; parallel extracranial organ seeding as specificity control	Paired brain and extracranial burden; bacterial FISH in the metastasis (D2)	Not performed
**Microbial‐side genetics: *ribD* or *ribA* (loss of ligand)**	Node 4	Mono‐colonization with an isogenic Δ*ribD* or Δ*ribA* mutant, which cannot form 5‐A‐RU, versus its wild‐type parent	MAIT‐dependent phenotype present with the wild‐type strain and absent with the mutant	Isogenic parent strain; equivalent colonization density confirmed by qPCR; 5‐OP‐RU add‐back rescue	MAIT frequency and activation; BM burden	Not performed
**Microbial‐side genetics: *ribH* (gain‐of‐ligand control)**	Node 4	Mono‐colonization with an isogenic Δ*ribH* (lumazine synthase) mutant, which accumulates the 5‐A‐RU precursor, versus its wild‐type parent	MAIT activation **enhanced**, not abolished; the reciprocal of the Δ*ribD* result and the control that distinguishes a ligand effect from a colonization effect	Isogenic parent strain; equivalent colonization density; measured 5‐OP‐RU or 5‐A‐RU in the lumen	MAIT frequency and activation; BM burden	Not performed
**Microbial‐side genetics: butyrate pathway**	Node 3	Mono‐colonization with a butyrate‐kinase or butyryl‐CoA‐transferase mutant versus parent	Barrier and colonization phenotype tracks butyrate production rather than the organism	Equivalent colonization density; **direct quantification of caecal and plasma butyrate**; butyrate add‐back rescue	Plasma and brain butyrate; BBB permeability; BM burden	Not performed
Germ‐free and gnotobiotic reconstitution	Nodes 1, 3	Germ‐free animals colonized with faecal communities from patients with versus without BM, or with a defined consortium	Transfer of the human phenotype to the recipient animal	Donor screening; multiple independent donors per group to avoid donor‐specific artifact; germ‐free is a comparator, not a control, because of its abnormal baseline	BM incidence and burden; barrier and glial readouts; metabolite quantification	Not performed

BM, brain metastasis; BBB, blood–brain barrier; FISH, fluorescence in situ hybridization; MAIT, mucosal‐associated invariant T cell. All entries are proposed experiments; none has been performed in an NSCLC brain‐metastasis model, which is why no node of the axis currently attains evidence level E1.

### 3.4 Disease‐relevant gene‐microbiome links in NSCLC and brain metastasis

Beyond canonical PRRs, several other host loci modulate microbiome–immune–cancer interactions relevant to NSCLC brain metastasis. HLA polymorphisms shape tumour neoantigen recognition, with HLA class I heterozygosity correlating with ICI response^82^, ^83^; HLA class II–restricted presentation of microbial peptides is a proposed but untested route by which the same locus might also shape gut microbiome composition (E4); bacteria‐derived peptides have been eluted from HLA molecules on human tumour cells, providing a direct molecular link between intratumoral bacteria and antigen presentation.^84^ FUT2 polymorphisms (secretor status) alter mucin glycosylation and gut microbiome composition with downstream effects on systemic immunity. The first human mycobiome GWAS identified 148 fungi‐associated host variants in a discovery cohort,^85^ including inflammatory and metabolic loci that may co‐determine intratumoral fungal composition. The loci discussed in this section are summarized in Table 9. Most of these candidate gene–microbiome links remain to be replicated in NSCLC brain metastasis cohorts; retrospective profiling of stool samples from existing NSCLC cohorts with germline sequencing and outcome data is a tractable next step.

**TABLE 9:** Host genetic loci and microbiome–immune–cancer interactions relevant to NSCLC and brain metastasis.

Gene / locus	Variant / allele	Pathway / function	Microbiome / immune effect	NSCLC / BM relevance	Refs
CARD9	rs10870077, rs4077515 (variant identities from the IBD fine‐mapping literature; not reported in^81^)	Dectin signalling adaptor; NF‐κB activation	Altered fungal/bacterial recognition; reduced IL‐17	IBD risk; candidate tumour immune modulator	^81^
CLEC7A (DECTIN‐1)	Y238X (early stop)	C‐type lectin; β‐glucan receptor	Defective fungal recognition; impaired IL‐17/IL‐22	Chronic candidiasis; potential tumour immune deficit	^76^
FUT2	rs601338 (non‐secretor)	Mucin glycosylation	Altered gut microbiome composition; reduced *Bifidobacterium*	Modified systemic immunity; ICI response candidate	^69^
EGFR	L858R; exon 19 del	NSCLC driver; receptor tyrosine kinase	Altered lung tumour microbial composition with *Streptococcus* enrichment has been proposed; the cited references report brain‐metastasis incidence, not microbial measurements	Highest BM rate (40%–60%); TKI‐sensitive	^4^, ^5^
KRAS	G12C, G12D	NSCLC driver; small GTPase	Distinct intratumoral microbial signature	Altered ICI response; ∼25% of NSCLC	^30^
ALK	EML4‐ALK fusion	NSCLC driver; receptor tyrosine kinase	Not measured: no study has profiled the lung tumour microbiome by ALK status	Elevated BM risk (50%–70%); younger patients	^4^, ^74^
TP53	Various LoF mutations	Tumour suppressor; inflammation regulation	Modulates tumour‐microbiome inflammatory crosstalk	∼50% of NSCLC; ICI response modifier	^71^
HLA Class I	Heterozygosity at HLA‐A/B/C	Antigen presentation	HLA‐restricted presentation of microbial peptides is established^84^; an effect of HLA genotype on microbiome composition in NSCLC is not measured	Correlates with ICI response	^82^, ^83^
LRRK2	rs11564258; G2019S	Kinase; macrophage function (NFAT regulation)	Regulates NFAT signalling in macrophages and modifies experimental colitis severity^86^; no microbiome sequencing was performed in that study, and a microbiome effect is inferred, not measured	Crohn link; NSCLC tumour expression	^86^
IL17A	rs2275913	Th17 cytokine	Modulates Th17/Treg balance	Lung cancer susceptibility; mucosal immunity	^87^

*Note*: All entries in this table are evidence level E4 with respect to NSCLC brain metastasis: each gene–microbiome or gene–immunity link has been established in another disease context and is extrapolated here. No row represents a demonstrated modifier of brain‐metastasis risk.

Abbreviations: BM, brain metastasis; ICI, immune checkpoint inhibitor; IBD, inflammatory bowel disease; LoF, loss of function.

### 3.5 Animal models and lung‐resident microbiome heritability

Mouse models provide mechanistic resolution for host–microbiome–cancer interactions. Genetically engineered lung cancer models can be crossed to germ‐free or gnotobiotic backgrounds. The autochthonous models for which this has actually been done are *Kras* ^LSL‐G12D^ and *Kras* ^LSL‐G12D^; *Trp53* ^fl/fl^ ^30^, ^88^; EGFR‐driven and EML4‐ALK models exist but have not, to our knowledge, been used gnotobiotically, and ALK F1174L is a neuroblastoma‐associated kinase‐domain mutation rather than an NSCLC driver allele; a limitation specific to Node 4 must be stated alongside them: MAIT cells constitute roughly 0.1% or less of T cells in specific‐pathogen‐free C57BL/6 mice, against the 1%–10% of circulating T cells reported in humans, so a null result in *Mr1* ^−/−^ or *Traj33* ^−/−^ animals on a standard background may reflect the small size of the compartment rather than its irrelevance, and MAIT‐enriched backgrounds or adoptive transfer should be used before a negative result is treated as decisive; such studies show germ‐free conditions alter NSCLC progression and immune infiltrate, and local manipulation of the pulmonary microbiota by aerosolized antibiotic or probiotic changes lung metastatic burden in mice,^31^ although direct germ‐free brain metastasis studies remain absent. Antibiotic‐induced dysbiosis perturbs tumour growth, intratumoral immunity, and metastasis frequency (E2). For brain metastasis specifically, intracardiac or intracarotid tumour cell injection across microbiome backgrounds offers tractable systems for premetastatic niche conditioning hypotheses. A key limitation is the low frequency of spontaneous brain metastasis in standard mouse NSCLC models, which forces reliance on experimental seeding that may not fully recapitulate premetastatic biology; this is the structural reason no result in this field currently reaches E1. The specific perturbation designs, and the controls that would make their results interpretable, are set out in Section 7.

The lung microbiome, distinct from but communicating with the gut, is shaped by host genetics and by smoking, air pollution and infection. Healthy lung harbours low‐biomass communities^89^, ^90^ of *Streptococcus*, *Prevotella*, *Veillonella* and *Pseudomonas*, shifting toward oral commensal enrichment in smokers, in chronic obstructive pulmonary disease^91^ and in cancer; because these are low‐biomass specimens, every compositional claim in this literature is D1 and is sensitive to reagent background.^50^, ^51^ Host genetic modifiers include mucin genes (MUC1, MUC5AC, MUC5B), surfactant proteins and antimicrobial peptide loci. Lung microbiome composition differs between lung tumours and adjacent or airway tissue and varies with histology and TP53 status^18^, ^71^; whether it diverges between driver‐mutation subgroups, or between patients who do and do not subsequently develop brain metastasis has not been tested, and Mendelian randomization in large NSCLC cohorts with paired germline and microbiome data would be required for causal inference.

### 3.6 Synthesis and outstanding questions

Host genetic architecture sits upstream of the microbiome–brain metastasis axis, simultaneously modulating microbiome composition, tumour‐intrinsic features, and anti‐tumour immunity. Three priority questions emerge: (i) do specific host variants identify NSCLC patients at high brain metastasis risk via microbiome‐mediated mechanisms; (ii) can pretreatment microbiome composition modify the genetic risk landscape; (iii) do driver mutations causally shape the lung tumour microbiome in ways relevant to metastatic potential, or reflect shared upstream factors? These questions are tractable using existing NSCLC molecular epidemiology infrastructure. None has yet been answered, and Section 6.6 assesses whether microbial features could add anything to the molecular risk models already in clinical use.

## 4 THE PROPOSED MICROBIOME–BRAIN METASTASIS AXIS

### 4.1 Why the microbiome might matter for brain metastasis

The premetastatic niche concept, introduced by Kaplan, Lyden, and colleagues^92^, ^93^ over the past two decades, established that distant organs are preconditioned for tumour cell colonization by signals derived from the primary tumour before metastatic seeding occurs. The brain also imposes distinctive constraints on colonization: an intact blood–brain barrier, resident microglial and astrocytic surveillance, and a metabolically specialized parenchyma. Yet despite these constraints, brain metastases occur in 30%–50% of NSCLC patients, often appearing months to years after primary diagnosis and frequently in the absence of progressing extracranial disease, implying that the cerebral compartment becomes selectively permissive at specific time points. The systemic microbiome is a candidate contributor to this selective permissiveness, for three reasons, none of which is itself evidence that it does contribute. Gut microbiome composition influences the systemic cytokine milieu, immune cell maturation programs, and BBB permeability through mechanisms that have been mechanistically dissected in models of neurodegeneration, depression, and stroke^26^, ^27^, ^28^, ^29^ (E2). Gut microbiome perturbation modulates anti‐tumour T cell function, immune checkpoint inhibitor (ICI) response, and tumour‐derived inflammation^7^, ^8^, ^9^ (E2–E3). By extension, the same microbiome‐derived signals that prime the brain for inflammation in neurodegenerative models could prime the brain for metastatic colonization (E4). Recent observational human studies have reported that gut microbial signatures distinguish lung cancer patients with versus without brain metastasis.^20^, ^21^ It is important to be precise about what this does and does not show. Those samples were collected from patients who already had brain metastases, so the comparison identifies a correlate of brain‐metastasis status (E3). It cannot establish that the microbial difference preceded the metastasis, and it is equally compatible with the metastasis, its treatment, or the greater systemic disease burden that accompanies it having produced the microbial difference.

The classical premetastatic niche involves four canonical features: hematopoietic progenitor recruitment driven by primary tumour secretion of VEGFR1‐mobilizing signals^92^; extracellular matrix remodeling through fibronectin and tenascin‐C deposition; vascular hyperpermeability mediated by VEGF and angiopoietin‐2; and immunosuppressive cell accumulation involving myeloid‐derived suppressor cells (MDSCs) and regulatory T cells. In the brain, additional cerebral‐specific events occur—astrocyte activation toward a reactive phenotype, microglial priming toward an inflammation‐permissive state, and tight junction modulation between brain microvascular endothelial cells. Three principal routes could in principle connect systemic microbial biology to cerebral niche conditioning. First, microbial metabolites including short‐chain fatty acids (SCFAs), tryptophan‐derived indoles and kynurenines, and bile acids circulate systemically and reach the brain at biologically active concentrations through specific transporters. Second, microbiome‐modulated immune cells (MAIT cells, NK cells, monocyte subsets) traffic to brain through BBB‐localized chemokine gradients. Third, microbial pathogen‐associated molecular patterns (LPS, peptidoglycan, β‐glucan) circulate at low levels and engage pattern recognition receptors on peripheral and BBB‐resident immune cells. Studies of gut microbiota effects on brain microglial maturation demonstrate^94^, ^95^ that microbiome composition shapes microglial transcriptional state long‐term; perturbation of this state through antibiotic exposure or germ‐free rearing produces aberrant microglial activation phenotypes, and germ‐free rearing increases BBB permeability with reduced occludin and claudin‐5 expression, an effect partly reversed by SCFA administration^26^ (E2). By analogous logic, microbiome‐shaped microglia in adult NSCLC patients may be more or less permissive to incoming tumour cells (E4). No direct evidence for this hypothesis exists in cancer. Neither Ref. 20 nor Ref. 21 sampled patients before brain metastasis developed, and neither reports a prospective prediction, so microbiome signatures cannot be said to predict brain‐metastasis occurrence prior to clinical detection.

### 4.2 Microbial metabolites and blood–brain barrier modulation

Microbial metabolites comprise the most directly tractable component of the axis, and their sources, transport routes and barrier and glial effects are summarized in Figure 2. Three classes dominate the relevant biology, and their microbial sources, receptors and reported disease associations are tabulated in Table 10. Short‐chain fatty acids (SCFAs), principally butyrate, propionate, and acetate,^96^, ^97^ are produced through bacterial fermentation of dietary fibre by Firmicutes and Bacteroidetes commensals. SCFAs reach portal circulation at high concentrations and engage the short‐chain fatty acid receptors FFAR3 (GPR41), FFAR2 (GPR43), and HCAR2 (GPR109A), and inhibit histone deacetylases; FFAR2 and HCAR2 assignments derive from *Ffar2* ^−/−^ ^58^ and *Hcar2* ^−/−^ ^59^ mice in colitis and colon carcinogenesis, and FFAR3 from *Ffar3* ^−/−^ mice in host energy metabolism^60^ (E2); systemic concentrations are substantially lower than portal levels, and direct receptor engagement at the blood–brain barrier remains incompletely characterized. Their effects on the BBB are reported to be dose‐dependent and biphasic,^97^ a pattern summarized in a review rather than established by a single primary dose–response study at the barrier (E2): physiological levels strengthen tight junction expression (claudin‐5, occludin) and reduce endothelial permeability, whereas supraphysiological levels can disrupt barrier integrity. SCFAs also modulate microglial function through both receptor engagement and HDAC inhibition. Depletion of *Marvinbryantia*, a Firmicutes genus described as a butyrate producer in^21^ but characterized in its original species description as a formate‐dependent homoacetogen that ferments glucose to acetate through the Wood–Ljungdahl pathway,^98^ so that the step from its depletion to reduced butyrate availability is itself uncertain, was a top machine learning‐derived discriminator of brain metastasis status in SCLC patients,^21^ and altered SCFA‐related metabolite profiles were observed in NSCLC brain metastasis patients^20^; these observations are consistent with a model in which BBB integrity and homeostatic microglial tone are compromised at the premetastatic stage, although neither study measured BBB integrity or microglial state, the specific butyrate‐producing taxa identified differ between the two studies, and the inference from taxonomic depletion to reduced butyrate availability was not confirmed by direct quantification of butyrate in either cohort. Tryptophan‐derived metabolites, including kynurenine, kynurenic acid, indoles, and indole‐3‐aldehyde, are produced through both host (IDO1/TDO2) and microbial pathways. Microbial indoles engage the aryl hydrocarbon receptor^99^, ^100^ (AhR) on barrier epithelial cells, dendritic cells, and astrocytes, where they regulate inflammatory tone; astrocyte‐ and microglia‐restricted AhR deletion established this pathway causally in experimental CNS inflammation^27^, ^28^ (E2). Kynurenine is generated principally by the host kynurenine pathway (IDO1/TDO2 and formamidase) rather than by bacterial tryptophanase, which instead yields indole; it crosses the BBB through the large‐neutral‐amino‐acid transporter LAT1^56^ and is the principal substrate for downstream neuroactive metabolites including quinolinic acid (NMDAR agonist) and kynurenic acid (NMDAR antagonist).^61^ The kynurenine pathway is elevated in patients with NSCLC and other solid tumours, and altered serum kynurenine‐to‐tryptophan ratios correlate with tumour immune escape.^62^ Recent NSCLC brain metastasis cohorts have detected altered tryptophan‐derivative profiles in patients with brain metastasis,^20^ suggesting a possible microbial contribution to systemic kynurenine biology, although host IDO1 induction by tumour‐associated inflammation is an equally parsimonious explanation that these cohorts cannot exclude. A separate NSCLC series has profiled plasma tryptophan metabolites as candidate immunotherapy‐response biomarkers, but enrolled no patients with brain metastasis and reported no brain‐metastasis endpoint.^101^ Secondary bile acids, produced through bacterial 7α‐dehydroxylation^63^, ^65^ of primary bile acids, modulate the regulatory T cell versus Th17 cell balance, with downstream effects on systemic anti‐tumour immunity. The mechanism reported in the primary work is not farnesoid X receptor or TGR5 signalling: 3‐oxolithocholic acid inhibits RORγt and suppresses Th17 differentiation, while isoallolithocholic acid promotes regulatory T cell differentiation through the *FOXP3* CNS3 enhancer,^63^ and bile‐acid metabolites maintain colonic RORγ+ regulatory T cells^64^ (E2, in mouse colon and systemic immunity; Table 7). Bacterial lipopolysaccharide circulates at low concentrations^66^ even in health and engages TLR4 on peripheral and CNS‐resident myeloid cells; elevated systemic LPS, as occurs with gut barrier dysfunction, can prime microglia towards a pro‐inflammatory phenotype that promotes neuroinflammation and is hypothesized to facilitate metastatic colonization (E4; no study has measured circulating LPS or LPS‐binding protein in a lung cancer brain‐metastasis cohort).

**FIGURE 2:**
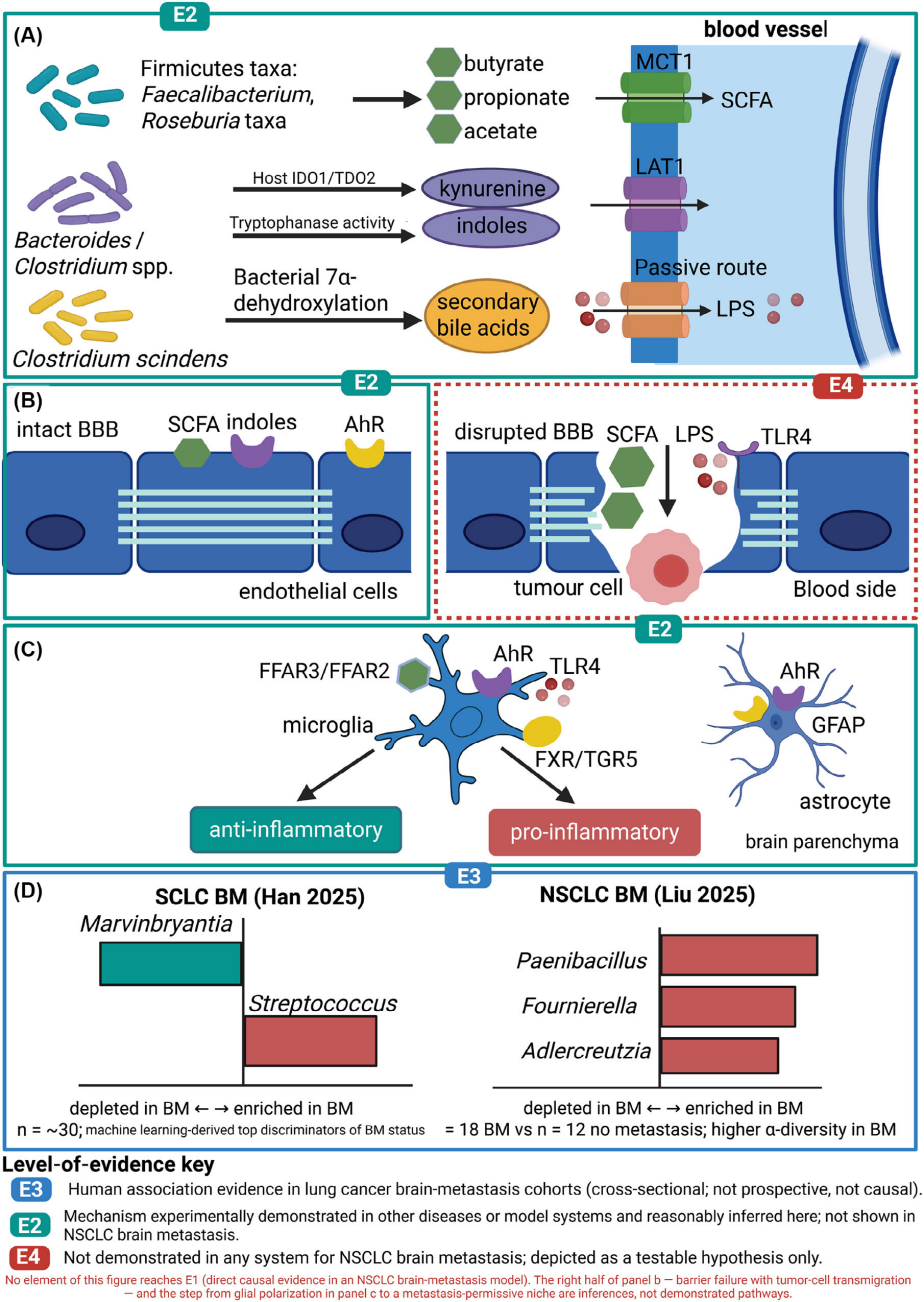
Microbiome‐derived metabolites at the gut–blood–brain interface and their proposed effects on the cerebral premetastatic niche, a hypothesized state rather than a demonstrated one. (A) Gut microbiota, dominated by Firmicutes and Bacteroidetes, produce three principal classes of CNS‐relevant metabolites: short‐chain fatty acids (SCFAs: butyrate, propionate, acetate) through fermentation of dietary fibre by *Faecalibacterium* and *Roseburia* taxa; tryptophan derivatives (indoles and indole‐3‐aldehyde through bacterial tryptophanase; kynurenine through the host IDO1/TDO2 pathway, which bacterial tryptophanase does not generate); and secondary bile acids (deoxycholate, lithocholate) through bacterial 7α‐dehydroxylation of primary bile acids. Transport routes are shown alongside: SCFAs reach the brain via MCT1, kynurenine via LAT1, and lipopolysaccharide and other PAMPs circulate at low concentrations. (B) The blood–brain barrier: at physiological concentrations SCFAs support tight junction protein expression (claudin‐5, occludin, ZO‐1) on brain microvascular endothelium and tryptophan metabolites engage endothelial AhR, whereas LPS‐driven TLR4 signalling loosens tight junctions; the right‐hand half depicts tumour cell transmigration through a disrupted barrier. (C) Microglial and astrocytic effects: SCFAs engage FFAR3 (GPR41) and FFAR2 (GPR43)—butyrate also engages HCAR2 (GPR109A), which Table 10 lists but this panel does not draw—and inhibit HDACs; tryptophan‐derived indoles engage AhR on astrocytes and microglia; bile acids engage FXR/TGR5; LPS engages TLR4 to promote pro‐inflammatory polarization. (D) Disease‐relevant signatures: *Marvinbryantia* depletion together with *Streptococcus* enrichment have been reported in SCLC patients with brain metastasis,^21^ while NSCLC brain metastasis cohorts show enrichment of *Paenibacillus*, *Fournierella*, and *Adlercreutzia* with altered SCFA‐related and tryptophan‐derivative metabolite profiles^20^; both cohorts are single‐centre and from the same research group, and independent replication is pending. **Data provenance**: Panel d redraws taxon‐level results reported in Ref. [20] (*n* = 18 NSCLC with brain metastasis versus *n* = 12 without distant metastasis; faecal shotgun metagenomics with untargeted metabolomics) and Ref. [21] (SCLC, approximately 30 patients; faecal metagenomics with machine‐learning feature ranking). Repository, platform and reported quality control for both are listed in Table 5. **Evidence annotation**: Coloured boxes state the evidence level of each element, as defined in Section 2.5 and Table 2: solid blue, human association evidence in lung cancer brain‐metastasis cohorts (E3); solid green, mechanism demonstrated experimentally in another disease or model system and extrapolated here (E2); red dashed, not demonstrated in any system for NSCLC brain metastasis and shown as a testable hypothesis only (E4). No element of this figure reaches E1. The relationship drawn here, from gut dysbiosis through altered metabolites and impaired barrier integrity to enhanced cerebral colonization, is an inference rather than a demonstrated causal chain. Panels prepared by the authors and created with BioRender.com; no previously published material is reproduced.

**TABLE 10:** Microbiome‐derived metabolites at the gut–blood–brain interface: Microbial sources, receptors, barrier and immune effects.

Metabolite class	Microbial source	Receptor / mechanism	Effect on BBB and immune compartment	Lung cancer BM relevance (NSCLC unless SCLC is stated)	Refs
Butyrate (SCFA)	*Faecalibacterium*, *Roseburia*, *Marvinbryantia*	FFAR2 (GPR43), FFAR3 (GPR41) and HCAR2 (GPR109A) agonist; HDAC inhibitor	Strengthens tight junctions; anti‐inflammatory microglial polarization	Producer taxa depleted in SCLC BM^21^; butyrate itself not quantified in either cohort (inferred; see Table 11)	^20^, ^21^
Propionate (SCFA)	*Bacteroides*, *Akkermansia*	FFAR3 (GPR41, preferred) and FFAR2 (GPR43) agonist; HDAC inhibitor	Strengthens TJ; Treg expansion	Not quantified; inferred from producer‐taxon abundance^20^ (see Table 11)	^20^
Acetate (SCFA)	Many commensals	FFAR2 (GPR43) agonist; weak FFAR3 activity	Modest TJ effects; microglial modulation	Not measured in any lung cancer brain‐metastasis cohort	^96^, ^97^
Kynurenine	Indirect: microbial tryptophan consumption and indole production alter host substrate availability; kynurenine itself is made by the host IDO1/TDO2 pathway, and bacterial tryptophanase yields indole, not kynurenine	LAT1 transport; AhR/NMDAR modulator	Modest direct BBB effect; astrocyte AhR signalling	Altered tryptophan‐derivative profile reported in one NSCLC BM cohort^20^; microbial versus host IDO1 origin not distinguished	^20^
Indole‐3‐aldehyde	*Lactobacillus*, gut commensals	AhR agonist	Tightens endothelial barrier; astrocyte modulation	Not measured in any lung cancer brain‐metastasis cohort	^99^, ^100^
Indoxyl sulphate	Gut commensals (downstream)	AhR agonist; oxidative stress	Disrupts TJ at high levels; pro‐inflammatory	Not measured in any lung cancer brain‐metastasis cohort	^99^, ^100^
Deoxycholate (BA)	Bacterial 7α‐dehydroxylation	FXR and TGR5 agonist; distinct oxo‐ and iso‐bile acids act on RORγt and FOXP3 directly^63^	Variable BBB effect; Treg/Th17 balance shifted by 3‐oxoLCA (RORγt binding) and isoalloLCA (FOXP3 CNS3 enhancer)^63^; bile‐acid metabolites also maintain gut RORγ+ regulatory T cells^64^	Not measured in any lung cancer brain‐metastasis cohort	^63^, ^65^
LPS / endotoxin	Gram‐negative bacteria	TLR4 agonist	Drives pro‐inflammatory microglial polarization and chronic neuroinflammation in rodent models^57^; no tight‐junction or barrier‐permeability readout was reported in that work	Not measured: no lung cancer brain‐metastasis cohort has reported circulating LPS or LPS‐binding protein	^66^
Peptidoglycan	Gram‐positive bacteria	NOD1/2; TLR2 agonist	Microglial activation; circulating PAMP	Not measured in any lung cancer brain‐metastasis cohort	^67^
Benzoic acid	Gut commensals; xenobiotic metabolism	Antimicrobial; AhR; uncharacterized CNS effects	Unknown direct BBB effect	Top ML discriminator of SCLC BM status	^21^

*Note*: Columns on receptor and mechanism are supported by experimental work in non‐cancer systems (E2). The lung cancer brain‐metastasis column reports what was measured in the cited cohorts and should not be read as a demonstrated mechanism; two rows report findings from the SCLC cohort^21^ and say so, because no NSCLC result exists for them. Table 11 states, for each metabolite, whether the metabolite itself was measured or its abundance was inferred from taxonomy.

Abbreviations: BBB, blood–brain barrier; BA, bile acid; K/T, kynurenine‐to‐tryptophan; ML, machine learning; SCFA, short‐chain fatty acid; TJ, tight junction.

### 4.3 Separating direct from extrapolated evidence for each metabolite mechanism

The metabolite literature draws overwhelmingly on neurological disease, inflammatory models and general cancer immunology, and it is easy to write about it in a way that reads as though the evidence came from NSCLC brain metastasis. Table 6 makes the provenance of each metabolite–mechanism pair explicit: the primary evidence source, the disease or model system in which it was obtained, whether the link to NSCLC brain metastasis is direct or extrapolated, whether the metabolite was measured or inferred, and the validation status.

Two conclusions follow from that table and govern how the rest of this Review should be read. First, no metabolite–mechanism pair in the axis has been demonstrated in NSCLC brain metastasis; the strongest entries are E2, obtained in germ‐free mice, experimental autoimmune encephalomyelitis, colitis and stroke models. Second, and more specifically, functional metabolic change must not be inferred from taxonomic abundance. A shift in the relative abundance of a butyrate‐producing genus does not establish that butyrate availability has changed: substrate supply, cross‐feeding, absorptive capacity, host consumption and transit time all intervene, and the relationship between taxonomic potential and realized metabolite flux is weak in human cohorts. Where the cited lung cancer studies performed untargeted metabolomics,^20^, ^21^ we report the metabolite result; where they reported only taxonomy, we say so and treat the functional claim as inference. Quantitative, targeted assays of plasma SCFAs, kynurenine‐to‐tryptophan ratio and bile acid pools, run alongside metagenomics in the same specimens, are the minimum requirement for any future study intending to make a functional claim.

### 4.4 Astrocyte and microglial priming by circulating microbial signals

Microglia are yolk sac‐derived and long‐lived, astrocytes are neuroectodermal, and both show transcriptional plasticity in response to systemic inflammatory signals.^102^, ^103^, ^104^ Tumour cell entry into the brain triggers astrocyte activation,^105^, ^106^, ^107^ formation of reactive astrocyte clusters around tumour microdeposits, and bidirectional metabolic crosstalk that can be either growth‐promoting or restrictive depending on context. Microglia in the metastatic niche initially attempt phagocytic clearance but can shift to tumour‐supportive phenotypes once metastasis is established, and together with astrocytes and recruited myeloid cells they define the brain tumour microenvironmental landscape.^108^ Gut microbiota influences microglial transcriptional programs through SCFA‐mediated HDAC inhibition and through circulating PAMP signals reaching the brain. Germ‐free mice show aberrant microglial phenotypes that can be partially rescued by SCFA supplementation, and antibiotic‐treated mice display similar microglial transcriptional dysregulation^94^ (E2). In aging and neurodegeneration models, gut dysbiosis primes microglia toward pro‐inflammatory polarization^94^, ^95^ and accelerates pathology, and colonization of germ‐free α‐synuclein‐overexpressing mice with microbiota from affected patients is sufficient to worsen motor phenotype and microglial activation,^29^ which is among the strongest available demonstrations that a gut community can causally set CNS myeloid tone (E2). By analogous reasoning, microbiome perturbation in NSCLC patients may prime the brain microenvironment toward a state that is paradoxically more permissive to metastatic colonization, through mechanisms that include impaired homeostatic surveillance, altered cytokine output, and compromised phagocytic clearance of disseminated tumour cells (E4). Astrocytes show parallel sensitivity to microbiome‐derived signals, particularly indoles engaging AhR, established by astrocyte‐ and microglia‐restricted *Ahr* deletion^27^, ^28^ (E2); an equivalent astrocyte‐restricted bile‐acid or FXR/TGR5 experiment has not been reported (E4). The convergent proposal is that microbiome composition, distant from the cerebral compartment, may shape the immune tone of the niche that incoming tumour cells will encounter. This biology is currently best characterized in mouse models of neurodegeneration; translation to human NSCLC brain metastasis cohorts is an active and tractable experimental priority, and has not been attempted. Microbial composition across the gut, lung tumour and brain metastasis compartments, with the detection tier reached in each, is summarized in Table 11.

**TABLE 11:** Microbial composition across gut, lung primary tumour and brain metastasis compartments in NSCLC.

Compartment	Healthy composition	NSCLC‐associated composition	Shifts associated with BM	Methodology	Detection tier attained	Refs
Gut	Firmicutes / Bacteroidetes dominant; high diversity	Cohort‐dependent: *Streptococcus*↑ in SCLC^21^; *Paenibacillus*, *Fournierella*, *Adlercreutzia* ↑ in NSCLC^20^	SCLC BM^21^: *Marvinbryantia*↓, *Streptococcus*↑; NSCLC BM^20^: *Paenibacillus*↑, *Fournierella*↑, *Adlercreutzia*↑, with higher α‐diversity reported	Stool 16S rRNA / shotgun metagenomics	D1 (stool is high biomass; the limitation here is design, not detection)	^20^, ^21^
Lung airway	*Streptococcus*, *Prevotella*, *Veillonella* (low biomass)	Oral commensal enrichment; *Tannerella*↑	Increased oral commensal translocation	BAL 16S rRNA / metagenomics	D1	^18^
NSCLC primary tumour	Not applicable (tumour tissue)	*Streptococcus* and *Veillonella* reads, and fungal reads (*Candida*, *Malassezia*, *Aspergillus*); intracellular localization has not been demonstrated in lung cancer	Higher frequency of fungus‐associated host cells reported; assignment of a microbe to a host cell is an inference (Section 5.8.4)	Tissue bacterial 16S profiling with LPS/LTA imaging^34^ plus computational mining of single‐cell data for bacterial and fungal reads^19^; **no ITS amplicon sequencing was performed in either study**	D1 for the compositional profile; D2 for the presence of bacterial structures, by LPS/LTA imaging in Ref. 34	^19^, ^34^
NSCLC brain metastasis	Generally considered sterile	Reads predominantly bacterial (*Streptococcus*, *Veillonella*, Enterobacteriaceae) with few fungal reads; no viable organism demonstrated	Reported kingdom‐level shift to bacterial dominance; specimens not within‐patient paired (Section 4.5)	Computational mining of separately generated public scRNA‐seq datasets; extraction blanks not available	**D1 only**. No in situ localization, no viability assay, no orthogonal replication	^19^
Plasma metabolome	Balanced SCFAs/kynurenines/bile acids	Reduced SCFAs; elevated kynurenine ratio; altered bile acids	Altered metabolite profile reported; targeted quantification of SCFAs not performed	Untargeted metabolomics (LC‐MS/MS)	Not applicable (metabolite assay)	^20^, ^21^
Oral microbiome	*Prevotella*, *Streptococcus*, *Veillonella*	Periodontal pathobiont enrichment	Periodontal pathogen overrepresentation	Saliva / buccal swab 16S	D1	^109^

*Note*: The detection‐tier column states the highest tier (Section 2.6, Table 2) reached for each compartment. Every tumour and brain‐metastasis entry rests on D1, nucleic‐acid detection, and none has been confirmed by spatial localization, viability assay or orthogonal replication. The brain‐metastasis row in particular should be read as describing sequence detected in a low‐biomass specimen, not a demonstrated resident community. Abbreviations: BAL, bronchoalveolar lavage; BM, brain metastasis.

### 4.5 The lung‐to‐brain microbial compositional shift

The cerebral compartment as it is proposed to be conditioned before tumour cell arrival is shown in Figure 3. A striking observation from recent single‐cell intratumour microbiome analyses is that NSCLC primary tumours show high frequencies of fungi‐associated host cells, whereas brain metastatic lesions are dominated by bacteria‐associated cells.^19^ Before considering what this might mean, the design of the underlying analysis must be stated plainly. The primary‐tumour and brain‐metastasis single‐cell libraries were not obtained from the same patients. They were generated in separate studies, in different laboratories and sequencing runs, deposited publicly, and subsequently aggregated and mined for microbial reads with the SAHMI pipeline.^53^ There is therefore a technical explanation for the kingdom‐level difference that the data cannot exclude: the two specimen sets carry different reagent, environmental and batch backgrounds, and low‐biomass microbial read profiles are exquisitely sensitive to exactly these differences.^33^, ^50^, ^51^ The field has already learned this lesson expensively, in the retraction of a high‐profile pan‐cancer microbial classifier whose signal proved to be largely technical.^14^, ^15^ This kingdom‐level compositional shift would, if biological, admit one of three possibilities: (i) bacteria, but not fungi, accompany metastasizing tumour cells through systemic circulation; (ii) bacteria are preferentially recruited to or proliferate within established brain metastases; or (iii) brain‐resident microbial communities, a controversial concept supported by some autopsy studies but subject to contamination concerns inherent to low‐biomass sequencing, selectively expand bacterial taxa. Each possibility carries distinct experimental and therapeutic implications. Mechanistic candidates for selective bacterial accompaniment include intracellular bacteria within metastasizing tumour cells, as documented in colorectal and oral squamous cell carcinoma by Galeano‐Niño and colleagues^35^ and in breast cancer by Fu and colleagues,^110^ and bacterial dissemination through an impaired gut vascular barrier has been shown to seed hepatic metastases in colorectal cancer models^111^ (E2). If lung tumours harbour both extracellular fungi and intracellular bacteria, only the intracellular component would survive shear forces and immune clearance during systemic dissemination, producing the observed bacterial predominance in brain metastases; this is a hypothesis (E4), and it has not been tested in any lung cancer system. Alternative mechanisms include direct bacterial dissemination through hematogenous routes from gut or lung microbiome reservoirs, with subsequent CNS recruitment. The observation that gut *Streptococcus* is enriched in SCLC patients with brain metastasis^21^ is consistent with hematogenous dissemination of gut commensals to the brain microenvironment, although direct evidence remains pending and the analogous NSCLC cohort^20^ reported a different set of enriched taxa.

**FIGURE 3:**
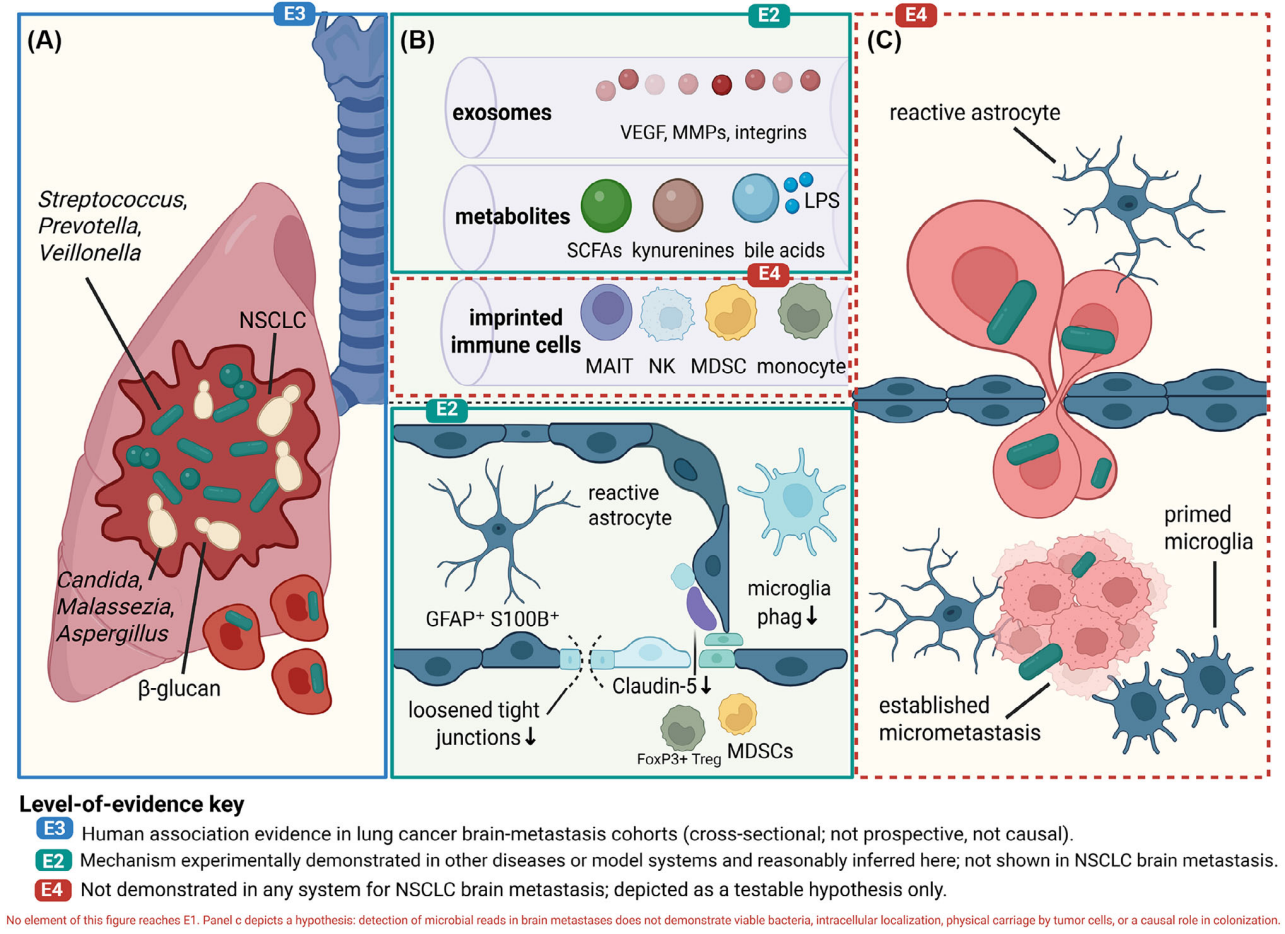
The cerebral premetastatic niche as proposed to be conditioned by microbiome‐derived signals; the niche and the influence are both hypotheses (Section 4.10). (A) Primary NSCLC tumour harbouring bacterial (*Streptococcus*, *Prevotella*, *Veillonella*) and fungal (*Candida*, *Malassezia*, *Aspergillus*) components, with β‐glucan available for Dectin‐1 engagement. (B) Tumour‐derived systemic mediators: exosomes carrying VEGF, MMPs and integrins; circulating microbial and host metabolites (SCFAs, kynurenines, bile acids, LPS); and microbiome‐imprinted immune populations (MAIT, NK, MDSC, monocytes). Below, the cerebral compartment showing reactive GFAP+S100B+ astrocytes, loosened endothelial tight junctions with reduced claudin‐5, reduced microglial phagocytic capacity, and recruitment of FoxP3+ regulatory T cells and MDSCs. (C) Tumour cell transmigration with associated bacteria, reactive astrocytes, primed microglia and an established micrometastasis. **Evidence annotation**: Coloured boxes state evidence level as defined in Section 2.5 and Table 2: solid blue (E3), human association evidence in lung cancer brain‐metastasis cohorts; solid green (E2), mechanism demonstrated in another disease or model system and extrapolated here; red dashed (E4), not demonstrated in any system for NSCLC brain metastasis. Panel c is annotated E4 in its entirety. Detection of microbial reads in brain metastases does not demonstrate viable bacteria, intracellular localization, physical carriage by tumour cells, or a causal role in colonization, and the depiction of bacteria traveling within transmigrating tumour cells is a hypothesis requiring within‐patient paired sampling, spatial localization, viability assays and experimental perturbation (Sections 2.6, 4.5 and 5.8). Panels prepared by the authors and created with BioRender.com; no previously published material is reproduced.

This compositional shift also has possible therapeutic implications, which we state conditionally because the antecedent is unproven. If bacteria within brain metastases are present, viable and biologically active, they represent a potential therapeutic target: selective antimicrobial intervention could in principle disrupt metastatic colonization without targeting host cells. Conversely, if bacterial sequence detection in brain metastases is a technical artefact, or reflects organisms that are a passive marker rather than an active driver, antimicrobial intervention would be ineffective or harmful. Distinguishing these possibilities is a critical experimental priority and is technically tractable using kingdom‐resolved spatial transcriptomics combined with germ‐free or antibiotic‐treated mouse models of NSCLC brain metastasis. Because the fungi‐to‐bacteria shift currently rests on a single SAHMI‐based reanalysis of separately generated public scRNA‐seq datasets,^19^ confirmatory experiments are essential: (i) *within‐patient* paired primary‐tumour and brain‐metastasis sampling with kingdom‐resolved spatial transcriptomics under stringent low‐biomass contamination controls, to reach detection tier D2; (ii) gnotobiotic and germ‐free murine NSCLC brain metastasis models with intracellular bacterial manipulation; and (iii) replication in independent multi‐centre cohorts using orthogonal detection chemistry, to reach D4. Until at least D2 is reached with within‐patient pairing, the kingdom‐level shift should be treated as a testable hypothesis and not as an established feature of NSCLC biology, and it should not be used as the rationale for an antimicrobial intervention.

### 4.6 Microbiome‐modulated immune cell trafficking to the cerebral compartment

The cerebral immune compartment is monitored not only by resident microglia and astrocytes but also by peripheral immune cells that traffic into the CNS through three routes: the BBB itself via diapedesis through brain microvascular endothelium, the blood‐CSF barrier at the choroid plexus, and the meningeal lymphatic system. Several peripheral immune populations are microbiome‐imprinted and have established roles in anti‐tumour immunity, making them candidate intermediates between gut microbiome and cerebral premetastatic niche. MAIT cells, semi‐invariant T cells^112^, ^113^, ^114^, ^115^ (TRAV1‐2/Vα7.2 paired with TRAJ33, TRAJ20, or TRAJ12 in humans) that recognize riboflavin‐biosynthesis intermediates presented on MR1 (Figure 4), can comprise up to approximately 10% of circulating T cells in some individuals (typical range 1%–10%, with substantial inter‐individual variability) and are enriched in liver and mucosal tissues. They are imprinted by microbial exposure during a defined early‐life window. MAIT cells exhibit dual function in cancer: cytokine‐driven activation produces anti‐tumour responses through IFN‐γ and granzyme B, whereas chronic activation can drive a pro‐tumour IL‐17‐producing phenotype. MAIT cell frequency in lung tumour infiltrate is altered in NSCLC, and recent work has documented MAIT cell accumulation in the inflamed central nervous system in experimental autoimmune encephalomyelitis,^46^ demonstrating that MAIT cells can traffic to and accumulate in CNS inflammatory contexts (E2). Natural killer (NK) cells, proposed to be microbiome‐modulated through gut‐derived SCFAs and bile acids (reviewed in^116^; no primary study has tested this in a lung cancer or brain‐metastasis setting), are key sentinels for circulating tumour cells and are recruited to the cerebral compartment under inflammatory conditions. Myeloid‐derived suppressor cells (MDSCs) and monocyte subsets^117^, ^118^ accumulate in the premetastatic niche and are sensitive to microbiome‐derived TLR signalling, particularly LPS engagement of TLR4 in CD14+ monocytes. The integrated picture is that microbiome composition in gut and lung tumour compartments shapes peripheral immune cell phenotypes, which in turn traffic to the brain and shape the local premetastatic milieu before tumour cell arrival. This biology has been extensively characterized for hepatic and pulmonary metastases but has not yet been examined for brain metastases. We are not aware of any study that has measured microbiome‐imprinted immune‐cell trafficking into the human or murine brain in a lung cancer context, and this node therefore stands at E4.

**FIGURE 4:**
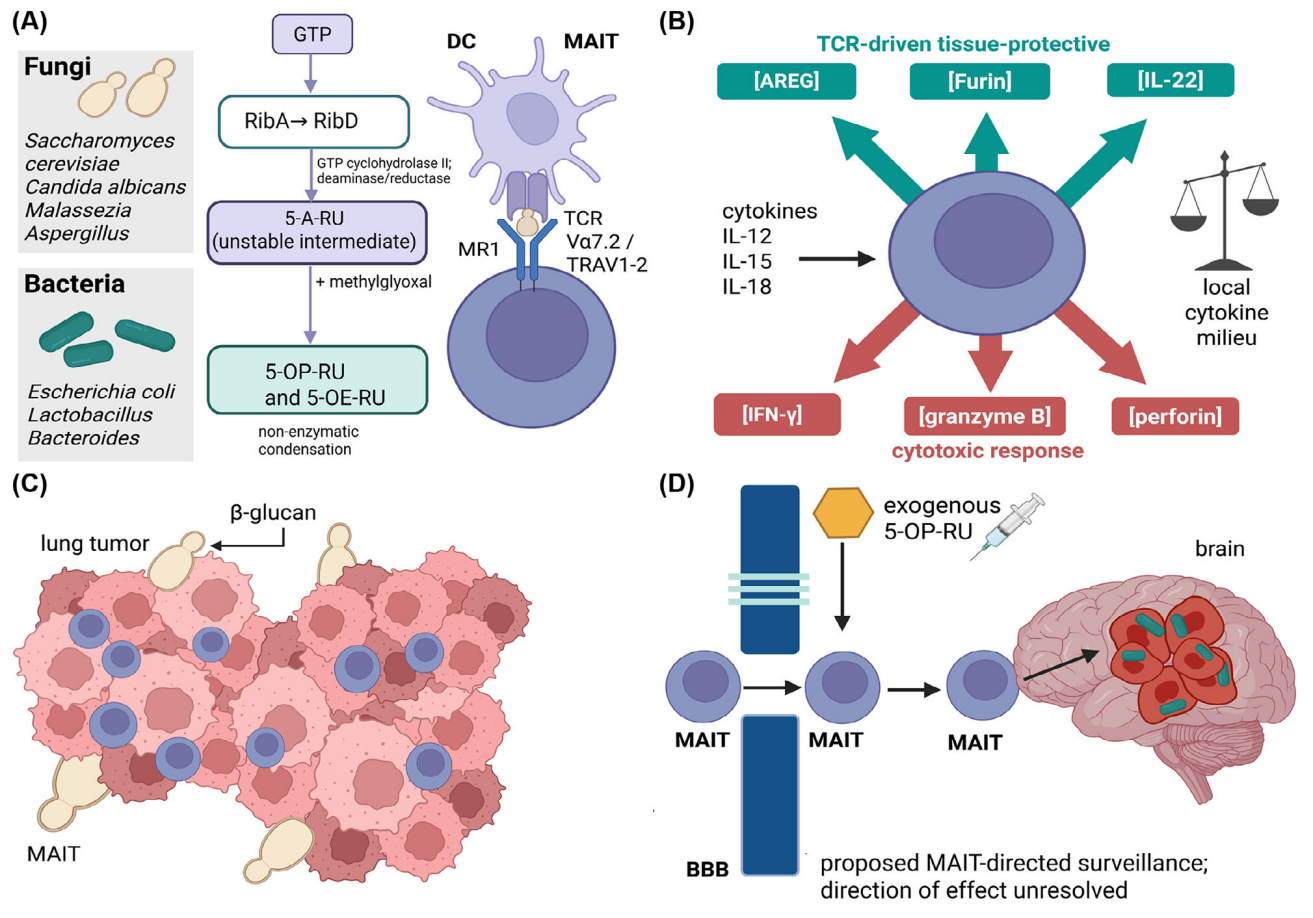
The MR1/MAIT cell axis in anti‐tumour immunity and brain metastasis surveillance. **(A)** Microbial riboflavin (vitamin B2) metabolism and MR1‐restricted recognition. 5‐A‐RU, an intermediate of the bacterial and fungal riboflavin pathway, condenses non‐enzymatically with methylglyoxal to form 5‐OP‐RU, which is captured by MR1 and presented to the semi‐invariant MAIT T‐cell receptor (Vα7.2/TRAV1‐2). The pathway is drawn directionally, from GTP through RibA and RibD to 5‐A‐RU and then, by non‐enzymatic condensation with methylglyoxal, to 5‐OP‐RU; riboflavin itself is the downstream end product of the same pathway and is not shown. **(B)** Divergent functional outcomes: T‐cell‐receptor‐driven activation supports a tissue‐protective program (amphiregulin, Furin, IL‐22), whereas cytokine‐driven activation (IL‐12, IL‐15, IL‐18) supports a cytotoxic program (IFN‐γ, granzyme B, perforin); the local cytokine milieu sets the balance. **(C)** MAIT phenotype in NSCLC: altered MAIT frequency and function in peripheral blood and tumour infiltrate, with depletion or exhaustion reported to associate with poorer outcomes; the source is a review of MAIT biology,^115^ not a primary NSCLC cohort. The panel is a schematic of MAIT cells within a lung tumour alongside fungal β‐glucan; it carries no quantitative data and none should be read from it. **(D)** Proposed relevance to the cerebral compartment: exogenous 5‐OP‐RU as a pharmacological MAIT modulator, MAIT trafficking across the blood–brain barrier under inflammation, and proposed brain‐metastasis surveillance, based on MAIT accumulation in the inflamed CNS in experimental autoimmune encephalomyelitis.^46^ **Evidence status**. Panels a to c are E2: the biochemistry and the two activation programs are established in infection and autoimmunity models. Panel d is E4: no study has examined MAIT cells in NSCLC brain metastasis in humans or in a model system. The molecular detail of the 5‐OP‐RU switch, and the pharmacological liabilities of targeting it, are treated in Sections 7 and 8.5. Panels prepared by the authors and created with BioRender.com; no previously published material is reproduced.

### 4.7 Blood‐brain barrier integrity, tight junctions, and microbial influence

The BBB consists of brain microvascular endothelial cells joined by tight junctions^119^, ^120^, ^121^ (claudin‐5, occludin, ZO‐1), supported by pericytes and astrocyte end‐feet, and modulated by neurovascular signals; claudin‐5 is the dominant determinant of size‐selective paracellular permeability, and its deletion in mice opens the barrier to small molecules without gross vascular disruption.^48^, ^49^ Tight junction integrity is sensitive to inflammatory cytokines^119^ (TNF‐α, IFN‐γ, IL‐6, IL‐1β), to oxidative stress, and to direct microbial signals. Tumour cell extravasation into the brain requires either passive BBB disruption (often inflammation‐mediated) or active transcytosis through endothelial cells; both can be modulated by systemic microbial signals. Multiple microbiome‐derived signals affect BBB function. SCFAs at physiological concentrations support tight junction protein expression and reduce endothelial permeability, and germ‐free mice have increased BBB permeability with reduced occludin and claudin‐5 that is partly corrected by mono‐colonization or sodium butyrate^26^ (E2), whereas low SCFA states (as inferred from depletion of butyrate‐producing *Marvinbryantia* in SCLC brain metastasis patients^21^ and altered SCFA‐related metabolite profiles in NSCLC brain metastasis patients^20^) may permit baseline BBB hyperpermeability; we stress that this is an inference from taxonomy and metabolite profiling in patients in whom no measurement of barrier function was made. In rodent models of stroke and neurodegeneration, gut microbiome perturbation produces measurable BBB permeability changes within days, and microbiome restoration partially reverses these changes (E2). By extension, lung cancer patients with reduced abundance of butyrate‐producing taxa and elevated systemic LPS load might be expected to exhibit baseline BBB hyperpermeability that increases susceptibility to metastatic colonization, although this end‐to‐end causal chain has not been tested in NSCLC or in any cancer model, and neither circulating LPS nor barrier permeability has been measured in the published lung cancer brain‐metastasis cohorts (E4). Pharmacological BBB stabilization, which would require agents that target endothelial tight junction proteins or that restore SCFA signalling, none of which has been shown to stabilize the human barrier for an anti‐metastatic purpose (E4; Section 8.6), represents an underexplored therapeutic strategy that could complement microbiome‐directed interventions, and endothelial‐restricted *Cldn5* deletion provides the genetic tool needed to test whether barrier integrity is on the causal path at all (Section 7, Table 8).

### 4.8 Intratumoral microbial reads and metastatic clone selection

The intratumoral microbiome of primary NSCLC tumours is detectable, and is a candidate modifier of metastatic behaviour. Pan‐cancer analyses^34^, ^54^ document detectable intratumoral microbial communities across essentially all examined cancer types with cancer‐type‐specific composition profiles. In lung cancer, intratumoral and lower‐airway microbial composition has been associated with histology and TP53 status^18^, ^71^ and with disease progression,^122^ and oral commensals (*Streptococcus*, *Veillonella*, *Prevotella*) are enriched in lung tumours, where that enrichment is associated with local inflammatory signalling.^18^ These are cross‐sectional human associations (E3/D1). The strongest reported link between tumour microbiome composition and survival comes from pancreatic, not lung, cancer,^123^ and the somatic‐genome and mutational‐signature analyses that define the lung histological classes themselves^124^, ^125^ included no microbial sequencing. Single‐cell resolution analyses further reveal that specific bacterial taxa associate with specific tumour cell phenotypes and immune cell subsets.^19^, ^35^ Mechanistically, intratumoral microbes may influence metastatic dissemination through (i) intracellular bacteria modulating PD‐L1, glycolytic phenotype, and motility^35^, ^110^, ^126^; (ii) local microbial metabolites shaping the tumour immune microenvironment, including presentation of bacteria‐derived peptides on tumour HLA molecules^84^; and (iii) circulating tumour‐derived DAMP/PAMP signals contributing to premetastatic niche conditioning. Whether the lung‐to‐brain bacterial bias^19^ reflects tumour‐intrinsic selection (clones harboring intracellular bacteria preferentially metastasize), a technical difference between the aggregated specimen sets (Section 4.5), or environmental selection within the cerebral compartment is unresolved (see Section 5.3 for further mechanistic dissection).

### 4.9 Cytokine milieu and the integrated immune landscape

The microbiome shapes systemic and local cytokine milieux that could condition the cerebral premetastatic niche. IL‐6 signalling through JAK–STAT3 supports tumour survival and inflammatory tone^127^ and is proposed to increase blood–brain barrier endothelial permeability, a step for which we are not aware of a primary measurement in a lung cancer brain‐metastasis system (E4), while SCFA‐driven regulatory T cell induction^44^, ^45^, ^128^ dampens anti‐tumour surveillance yet supports barrier integrity. The net cytokine balance, rather than any single mediator, determines whether the cerebral microenvironment is permissive or restrictive for metastatic colonization. No study has measured plasma cytokines alongside gut metagenomics in a lung cancer cohort with brain‐metastasis outcomes, so the entire content of this subsection is E2 with respect to the individual cytokine–barrier relationships and E4 with respect to their relevance to NSCLC brain metastasis. Establishing the causal chain requires paired gut metagenomics, plasma cytokine and quantitative metabolite profiling, and contrast‐enhanced permeability imaging in the same patients, as specified in Section 8.8.

### 4.10 The integrated axis

These mechanisms together describe a tractable and falsifiable hypothesis, which we state as a hypothesis rather than as a cascade. Gut and lung microbiome compositions, shaped by host genetics and environmental factors, produce circulating metabolites and PAMP signals that could modulate BBB tight junction integrity, microglial and astrocytic activation state, and the cerebral cytokine milieu—and thereby establish a permissive or restrictive premetastatic state for incoming tumour cells. Microbiome‐modulated MAIT, NK, monocyte, and MDSC populations may traffic to the cerebral compartment in parallel; intratumoral microbes within primary lung tumours may influence metastatic clone selection, and the reported bacterial predominance in brain metastases^19^ has been interpreted as selective bacterial accompaniment during dissemination, an interpretation that the underlying data cannot currently support (Section 4.5); and host genetic factors (CARD9, CLEC7A, FUT2; EGFR, ALK, TP53, HLA) are proposed to operate at every level. Kingdom‐resolved attribution of cerebral microbial signals to specific gut, lung, or intratumoral sources remains technically demanding, particularly given the low microbial biomass and contamination risks of cerebral tissue analysis; the framework is therefore structured to be falsifiable through prospective cohorts with rigorous contamination controls, kingdom‐resolved meta‐transcriptomics in within‐patient matched primary‐metastasis pairs, and gnotobiotic mouse models. Applying the grading scheme of Section 2 to this integrated picture gives the result shown in Figure 1b: three of the five nodes have established E2 support for their constituent biology in other systems, one has partial or indirect E2 evidence only, and the host‐genetics node has none; every node rests on E4 extrapolation for its relevance to lung cancer brain metastasis, and none has E1 support. The axis is a hypothesis with a defined experimental agenda, which Section 7 specifies.

## 5 CROSS‐COMPARTMENT MICROBIAL CROSSTALK: GUT–LUNG–BRAIN COMMUNICATION

### 5.1 The gut–lung axis in cancer

The gut–lung axis describes bidirectional communication^129^ between gastrointestinal and pulmonary mucosal sites through shared lymphatic drainage, systemic immune signalling, and microbial metabolite exchange; the three compartments considered in this section, with the routes proposed to connect them, are shown in Figure 5. In cancer, this axis bears directly on NSCLC: gut dysbiosis perturbs anti‐tumour immunity in the lung, and gut microbiome composition is associated with ICI response in NSCLC patients.^7^, ^8^, ^9^ The gut–lung axis is proposed to extend to brain through gut‐derived metabolites and immune cells that reach both pulmonary and cerebral compartments, providing a substrate for coordinated multi‐organ effects on NSCLC progression and brain metastasis. Recent NSCLC and SCLC brain metastasis cohort studies have reported associations between gut microbiome composition and both brain metastasis status^20^, ^21^ and radiotherapy response in established brain metastasis,^22^ providing initial human‐cohort evidence (subject to the limitations discussed in Section 6.10) that gut microbial composition is associated with intracranial clinical endpoints in lung cancer. No component of the cerebral compartment was measured in any of these studies; the brain enters only as a clinical endpoint.

**FIGURE 5:**
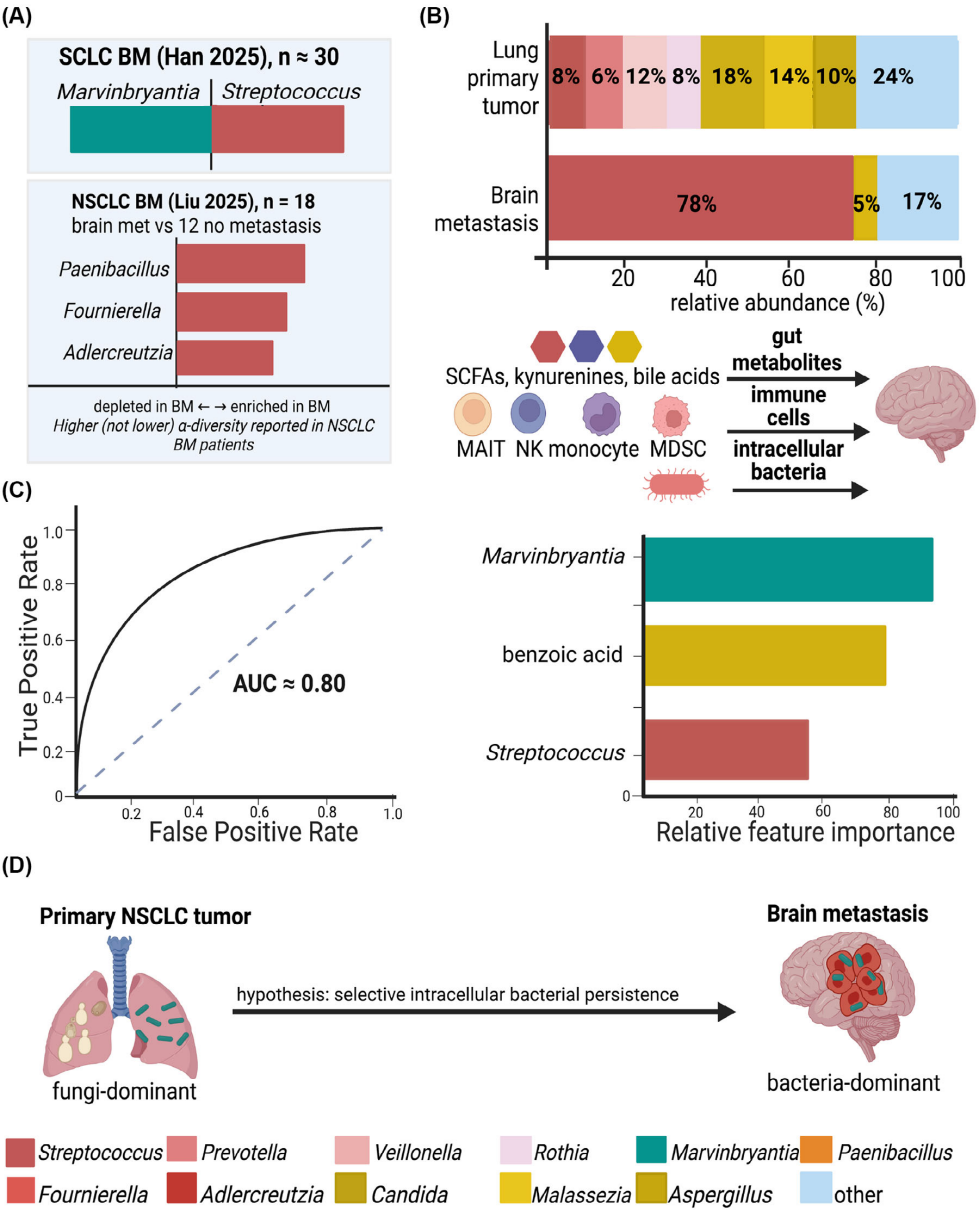
Cross‐compartment microbiome architecture in NSCLC: gut, lung tumour, and brain metastasis compartments, the routes of communication between them, and the microbial and metabolite species proposed to traverse each route. (A) Gut microbial features reported in the two single‐centre lung cancer cohorts: *Marvinbryantia* depletion with *Streptococcus* enrichment in SCLC brain metastasis,^21^ and *Paenibacillus*, *Fournierella* and *Adlercreutzia* enrichment with higher (not lower) α‐diversity in NSCLC brain metastasis.^20^ (B) Lung tumour compartment: reads from oral commensals (*Streptococcus*, *Prevotella*, *Veillonella*, *Rothia*) are detectable in primary NSCLC,^18^, ^34^ and fungal reads (*Candida*, *Malassezia*, *Aspergillus*) in the single‐cell mining study^19^; ^34^ profiled bacteria only. **The relative‐abundance values drawn in this panel are schematic: they illustrate qualitative composition and are not directly reported numerical proportions from any cited study**. (C) The machine‐learning classifier performance shown is the internally cross‐validated estimate reported for the SCLC cohort^21^ and is expected to be optimistic (PROBAST high risk of bias, analysis domain; Table 3); it is reproduced here for orientation and not as a validated operating characteristic. **The curve shape itself is schematic**:^21^ **reports a summary AUC only, with no published ROC curve, no confidence band and no operating point. An empirical cross‐validated ROC from a cohort of roughly 30 patients would be a step function with at most a few dozen vertices and a wide confidence band; the smooth curve drawn here conveys a precision the underlying data do not support**. The ranking beside the ROC curve shows the three discriminators reported in Ref. [21]—*Marvinbryantia*, benzoic acid and *Streptococcus*—in the order reported; the bar heights are relative and are not published importance values. (D) Reported predominance of bacterial over fungal reads in brain metastasis specimens relative to primary tumours^19^; the specimens compared were not within‐patient paired, and the arrow depicting selective bacterial accompaniment of disseminating tumour cells is a hypothesis (E4), not a demonstrated route (Sections 4.5 and 5.5). **One caution about the artwork: the words “selective intracellular bacterial persistence; shear‐force survival” are printed on the arrow itself, which gives a hypothesis the appearance of a labelled mechanism. They should be read as the proposal set out in** Section 5.3 **, not as a finding. The kingdom‐level comparison reported in Ref. [** ^19^] **is between the relative frequencies of host cells carrying reads of each kingdom, not between relative abundances; the stacked bars of panel b are schematic, as stated above. Data provenance**: Panel a redraws taxon results from Refs. 20 and 21; panel b is schematic, as stated above; panel c redraws the internally cross‐validated classifier performance reported in^21^; panel d depicts the kingdom‐level comparison reported in,^19^ a SAHMI‐based reanalysis of separately generated public single‐cell datasets. Accessions, platforms, specimen numbers and reported quality control for all four sources are listed in Table 5. **Evidence status**: Compartment compositions are detection tier D1 throughout (Table 11). Arrows between compartments denote proposed routes of communication, not demonstrated fluxes; no study has traced a defined microbial or metabolite species from gut or lung to the cerebral compartment in lung cancer. Panels prepared by the authors and created with BioRender.com; no previously published material is reproduced.

### 5.2 Microbial reads in the lung tumour compartment

NSCLC tumours harbor distinctive intratumoral microbial communities that differ from healthy lung and adjacent normal tissue. Oral commensals (*Streptococcus*, *Prevotella*, *Veillonella*, *Rothia*) are enriched in lung tumors^18^, ^122^ (E3/D1), likely through microaspiration and impaired clearance, and the tumour microbiome composition varies with histology and TP53 status^71^ (E3), and lower‐airway enrichment of oral commensals including *Veillonella* is associated with worse progression in lung cancer^122^ (E3); a specific prognostic association for *Streptococcus* has been proposed but not established. In a mouse model, commensal‐driven γδ T cell inflammation promoted lung tumour development^30^ (E2). Whether these sequences correspond to a viable, metabolically active community in human NSCLC is untested (D1; Table 11). In mouse models the lung community is functionally consequential: local PAMP production drives cytokine signalling and immune infiltrate modulation; the clearest causal evidence comes from mouse models in which local commensals drive γδ T17 inflammation and lung tumour growth^30^ and in which aerosolized antibiotic or probiotic administration changes pulmonary metastatic burden^31^; in pancreatic cancer, ablation of the intratumoral bacterial community reverses intratumoral immune suppression and restores sensitivity to checkpoint blockade,^79^ which is the closest existing demonstration that an intratumoral community is functionally consequential rather than incidental (E2). Intratumoral microbes have also been documented within tumour cells themselves,^35^, ^110^ where intracellular bacteria modulate phenotypes including PD‐L1 expression, metabolic reprogramming, and motility. Whether lung tumour microbial composition differs between NSCLC patients who do and do not subsequently develop brain metastasis has not been tested (Section 3.5), and no longitudinal evidence links any lung tumour signature to brain metastasis development, and single‐cell analyses^19^ reveal heterogeneity at cellular resolution.

### 5.3 Intracellular bacteria in tumour cells and metastatic potential

A particularly striking finding from the past 5 years is that some intratumoral bacteria are intracellular, residing within tumour cells and immune cells in oral squamous cell and colorectal carcinoma, where the localization was confirmed by fluorescence in situ hybridization and spatial profiling (D2)^35^; no equivalent single‐cell‐resolution or intracellular localization work has been performed in lung cancer. Bacterial lipopolysaccharide and lipoteichoic acid have been imaged in lung tumour tissue in the pan‐cancer survey,^34^ which reaches D2 for the presence of bacterial structures in the primary tumour; nothing at that tier has been reported for an NSCLC brain metastasis, where every claim remains at D1. Single‐cell sequencing of human tumours paired with bacterial DNA detection has revealed cell type‐specific bacterial associations: certain bacterial taxa preferentially associate with epithelial tumour cells, others with macrophages, others with T cells. Intracellular bacteria within tumour cells modulate cellular phenotypes including immune evasion through PD‐L1 induction, metabolic reprogramming towards glycolytic phenotypes, and acquisition of motile and mesenchymal characteristics that promote dissemination; in oral and colorectal cancer these localizations were confirmed by fluorescence in situ hybridization and spatial profiling, reaching detection tier D2,^35^ and in breast cancer intracellular bacteria were shown to promote metastatic colonization through resistance to fluid shear stress^110^ (E2). These results in other tumour types make intracellular persistence a plausible, but unverified, explanation for the bacterial predominance reported in NSCLC brain metastases.^19^ No comparable localization or functional work has been performed in lung cancer, and the NSCLC observation itself remains at D1 (Section 4.5). Whether specific intracellular bacterial species confer brain tropism, and whether they actively contribute to cerebral colonization versus passively accompany metastasizing tumour cells, are critical unanswered questions. Experimental dissection requires manipulation of intracellular bacterial load in NSCLC models combined with brain seeding assays, methodologies that are now technically tractable and are specified in Table 8. The mechanism also has therapeutic implications: selective targeting of intracellular bacteria, achievable through narrow‐spectrum intracellularly active antimicrobials, may represent an underexplored anti‐metastatic strategy in NSCLC patients at high risk for brain metastasis, although we emphasize that proposing an antimicrobial intervention on the basis of D1 evidence would be premature, and that antibiotic exposure is itself associated with worse immunotherapy outcomes in NSCLC^37^, ^130^.

### 5.4 The MR1/MAIT cell axis in anti‐tumour immunity

The MR1/MAIT axis represents one of the most direct molecular bridges between microbiome biology and anti‐tumour immunity, linking microbial vitamin B2 metabolism to T cell‐mediated tumour surveillance. MAIT cells, semi‐invariant T cells (TRAV1‐2/Vα7.2 paired with TRAJ33,^112^ TRAJ20, or TRAJ12 in humans; Vα19‐Jα33 in mice; see Figure 4) recognize riboflavin‐biosynthesis intermediates (principally 5‐OP‐RU, condensed non‐enzymatically from microbial 5‐A‐RU and methylglyoxal) presented on MR1, a non‐classical MHC class I‐like molecule.^131^ The discovery chain is worth stating precisely, because it is what makes this node unusual: MR1 was shown to present microbial vitamin B metabolites to MAIT cells^132^; the activating ligand was identified as 5‐OP‐RU, formed non‐enzymatically from the riboflavin‐pathway intermediate 5‐A‐RU and a reactive carbonyl^133^; and structural work has since defined how ligand potency is determined by the covalent Schiff base formed with MR1 Lys43.^134^ Because the riboflavin biosynthesis pathway is present in most bacteria and many fungi but absent from mammalian genomes, 5‐OP‐RU and its close congeners are the only known class of *microbially derived* MR1 agonist and cannot be produced by the host. MR1 can also present non‐riboflavin ligands—drug and drug‐like small molecules that modulate MAIT responses,^135^ and an as‐yet‐unidentified MR1‐presented ligand on tumour cells, recognized by MR1‐restricted T cells that are not MAIT cells^136^—so 5‐OP‐RU is the dominant and best‐characterized agonist class rather than the only one. MAIT cell activation through this route is therefore an essentially unambiguous report of microbial metabolic activity, which is what makes the MR1–5‐OP‐RU pair an attractive molecular switch connecting microbial metabolism to immune surveillance (Section 8.5). The functional consequence is established in infection models: MAIT activation and accumulation in vivo depend on microbial riboflavin synthesis, riboflavin‐pathway‐deficient organisms failing to elicit the response that their pathway‐competent counterparts do,^137^ and MAIT cells confer MR1‐dependent protection against pulmonary bacterial infection^138^ (E2). MAIT cells can comprise up to approximately 10% of circulating T cells in some individuals, with typical frequencies of 1%–10% and substantial inter‐individual variability, and are enriched in liver, mucosal tissues, and peripheral tumour infiltrate. In NSCLC, MAIT cell frequency and phenotype are reported to be altered in tumour infiltrate and peripheral blood, with reduced cytotoxic function in advanced disease; this summary is drawn from a review of MAIT biology^115^ rather than from a primary NSCLC cohort, and we have not found a primary series reporting MAIT frequency against a brain‐metastasis endpoint. MAIT cell activation produces dual functional outputs depending on activation mode and cytokine milieu: TCR‐driven activation through MR1‐5‐OP‐RU induces a tissue‐repair program (amphiregulin, Furin, IL‐22) that supports epithelial integrity, while cytokine‐driven activation (IL‐12, IL‐15, IL‐18) drives a Th1‐like cytotoxic program (IFN‐γ, granzyme B, perforin) that mediates direct anti‐tumour function. MAIT cells accumulate in the inflamed CNS in experimental autoimmune encephalomyelitis with both MAIT17 and MAIT1/17 subsets, suggesting potential involvement in brain metastasis surveillance.^46^ Therapeutic targeting of MR1/MAIT, through exogenous 5‐OP‐RU or pharmacological MAIT modulators, is an emerging strategy that may have specific utility in NSCLC brain metastasis control and may complement existing ICI approaches; the pharmacological obstacles to doing so safely are substantial and are set out in Section 8.5.

### 5.5 Direct microbial translocation to the brain in NSCLC

Whether and how microbes directly reach the brain in NSCLC patients remains controversial. The healthy brain is generally considered sterile, but reports of fungal and bacterial DNA in brain tissue have suggested possible low‐biomass CNS microbial communities, with intensified scrutiny in cancer contexts; the bacterial evidence in tumours is from pan‐cancer profiling^34^ and the fungal evidence from computational mining of single‐cell data.^19^ Plausible translocation routes include (i) hematogenous dissemination from gut or lung reservoirs requiring BBB compromise; (ii) neural routes via olfactory or vagal nerves, documented for certain neurotropic pathogens; (iii) accompaniment within metastasizing tumour cells, particularly intracellular bacteria.^35^, ^110^ All three are E4 in lung cancer: none has been demonstrated, or excluded, in any NSCLC system. Single‐cell evidence of bacterial enrichment in NSCLC brain metastases versus primary tumours^19^ is compatible with intracellular bacterial accompaniment, but it is equally compatible with differential contamination background between the separately generated specimen sets that were compared (Section 4.5), and it does not exclude the other routes. Low‐biomass CNS sequencing is highly susceptible to contamination and requires negative extraction blanks, contamination‐aware bioinformatics, orthogonal validation (FISH, culture), and cross‐laboratory reproducibility; none of these has been reported for a brain metastasis specimen in lung cancer. Whether detected bacteria are active drivers or passive markers of metastatic colonization is unresolved, and on present evidence it is not established that viable bacteria are present in NSCLC brain metastases at all.

### 5.6 Bacterial‐fungal cross‐kingdom interactions in NSCLC tumours

Lung tumours harbour both bacteria and fungi, and the interactions between these kingdoms are functionally consequential in other systems, although no study has measured any function of either compartment in NSCLC tissue. Cross‐kingdom signalling occurs through bacterial–fungal biofilm formation, competitive metabolism, and immune cross‐regulation. In NSCLC primary tumours, fungi associate with specific host cell types^54^ (E3/D1); the functional work linking a tumour mycobiome to anti‐tumour immunity has been performed in pancreatic^139^ and colorectal^140^ cancer, not in lung (E3/D1) and are proposed to contribute to local immunity through β‐glucan recognition via Dectin–CARD9 signalling (E4 in lung cancer; E2 in fungal immunology). Fungal taxa documented in NSCLC tumour tissue include *Candida*, *Malassezia*, *Aspergillus*, and *Cladosporium*, with composition varying by histology, anatomical location, and inflammatory state. The recent observation that NSCLC primary tumours are fungi‐enriched but brain metastases are bacteria‐dominant^19^ has been read as suggesting that the fungal compartment may be more strongly constrained to the primary tumour microenvironment, while bacteria preferentially accompany metastatic dissemination; as set out in Section 4.5, the comparison that generated this observation was between specimen sets from different patients and different sequencing batches, so a technical origin for the difference has not been excluded. Mechanisms for this differential trafficking include intracellular bacterial persistence in tumour cells (in contrast to predominantly extracellular fungi), differential survival in systemic circulation, or selective fungal sequestration in lung tissue through size or surface characteristics. The therapeutic implications include the possibility that selective intervention against either bacterial or fungal components could differentially affect primary tumour versus metastatic biology in NSCLC. Note that fungal contamination in low‐biomass tumour sequencing remains a substantial methodological concern; independent validation with orthogonal methods is essential, and is the subject of Section 5.8.

### 5.7 Barrier integrity across gut, lung and brain compartments

The gut–lung–brain communication relies on intact yet selectively permeable barriers at each compartment. Gut barrier dysfunction (leaky gut) elevates systemic LPS load and is associated with cancer, neurodegeneration, and altered immunity; in colorectal cancer models, impairment of the gut vascular barrier is sufficient to permit bacterial dissemination and to promote hepatic metastasis, which is the closest existing analogue to the process proposed here and is itself E2 evidence from another organ and tumour type.^111^ Pulmonary barrier dysfunction permits lung tumour microbiome enrichment and creates a permissive environment for tumour‐associated inflammation. BBB dysfunction would permit both microbial signalling and tumour cell extravasation into brain; no study has measured barrier permeability in NSCLC patients before the brain metastasis occurred, so whether such dysfunction precedes clinical detection of metastasis is unknown. Pharmacological barrier stabilization through agents targeting tight junction proteins (claudin‐5 modulators), microbiome‐derived metabolites that strengthen barrier function (butyrate, indoles), and dietary interventions that support mucosal health represent underexplored therapeutic targets that may complement immunotherapy; whether they alter brain metastatic colonization in NSCLC is untested.

### 5.8 Methodological caution in low‐biomass microbial analysis: what detection can and cannot support

Almost every microbial claim in this Review derives from sequencing of specimens with extremely low microbial biomass: lung tumour tissue, brain metastasis tissue and, in the case of computational mining, host transcriptomes not generated for microbial analysis at all. This is the single greatest threat to the validity of the field, and it warrants its own treatment rather than a caveat appended to individual results.

#### 5.8.1 Why low‐biomass specimens fail

Four failure modes recur. **Reagent and laboratory contamination**: DNA extraction kits, polymerases, water and laboratory surfaces carry a reproducible bacterial DNA background that dominates the signal once template input falls below roughly 10^3^–10^4^ genome copies, and that differs systematically between kit lots, extraction batches and laboratories.^50^, ^51^ When cases and controls are processed in different batches, this background becomes a perfect confounder of group assignment. **Index hopping and cross‐contamination on the sequencer**: Patterned flow cells misassign a small percentage of reads between multiplexed libraries; against a genuine signal of a few hundred reads, bleed‐through from a high‐biomass library on the same run is sufficient to create an apparent community. **Database misclassification**: Short reads from conserved regions, host‐derived sequences and low‐complexity regions are routinely misassigned to bacterial taxa, and reference databases contain contaminated assemblies. **Analytic artefact**: The most consequential demonstration is the retraction of a widely cited pan‐cancer classifier of tumour‐associated microbial reads^15^ after independent reanalysis showed that its apparent discriminative power was generated by data‐processing choices and contamination rather than biology.^14^ That episode should inform how every claim in this Review, including the lung‐to‐brain kingdom shift, is weighted.

#### 5.8.2 The controls that should be mandatory

At minimum, a study reporting microbial content of tumour or brain tissue should include, and report: negative extraction blanks processed in parallel with every batch at a defined ratio (we suggest at least one blank per eight specimens); no‐template amplification controls; positive controls consisting of a defined mock community spanning several orders of magnitude of input; randomized, batch‐matched allocation of cases and controls across extraction and sequencing runs; quantification of total bacterial load by qPCR against a standard curve, so that relative abundance is interpretable; statistical decontamination with a prevalence‐ and frequency‐based method such as decontam^32^ or source‐tracking with SCRuB,^33^ with the identity and abundance of removed taxa reported rather than silently discarded; and deposition of raw reads including blanks. None of the four lung cancer brain‐metastasis studies reviewed here^19^, ^20^, ^21^, ^22^ reports this full set, and the computational reanalysis^19^ could not have done so, because the underlying libraries were generated for other purposes and no blanks exist.

#### 5.8.3 Moving beyond detection: localization, viability and reproducibility

Controls constrain false positives; they do not convert a sequence into an organism. Advancing from D1 to D2 requires demonstrating that microbial structures are physically present in the tissue and where they sit relative to tumour and immune cells. Bacterial 16S fluorescence in situ hybridization with probe‐competition controls, RNAscope for bacterial transcripts, immunostaining for lipopolysaccharide or peptidoglycan, immuno‐electron microscopy for intracellular localization, and kingdom‐resolved spatial transcriptomics are all established, and were used to establish intracellular and microniche localization in oral and colorectal carcinoma.^35^ Advancing from D2 to D3 requires evidence of viability: culturomics under conditions appropriate to fastidious organisms with a stated limit of detection, propidium monoazide sequencing to exclude free and non‐viable DNA, rRNA‐to‐rDNA ratios, metatranscriptomics demonstrating active transcription, or stable‐isotope probing demonstrating metabolic incorporation. Advancing to D4 requires independent cohorts, at least two orthogonal chemistries, and within‐patient paired primary and metastatic specimens.

#### 5.8.4 Specific limitations of single‐cell microbial detection

Computational recovery of microbial reads from single‐cell RNA sequencing, of which SAHMI^53^ is the leading implementation, is a powerful idea whose limitations are specific and material. The assay was not designed for microbial detection: poly(A) selection depletes most bacterial transcripts, so recovered reads are a small, biased and lysis‐dependent subset. Ambient RNA and barcode swapping distribute reads across droplets, so the assignment of a microbe to a particular host cell is an inference rather than an observation of physical association, and the apparent frequency of microbe‐associated cells depends on ambient‐RNA correction. Single‐cell suspensions are prepared by enzymatic dissociation using reagents that carry their own bacterial background. Most importantly, retrospective mining cannot supply the negative controls that the method requires, because they were never collected. These constraints do not invalidate the approach; they mean that a single‐cell microbial result is a hypothesis‐generating D1 observation that requires orthogonal confirmation in the same tissue, and that comparisons between datasets generated in different laboratories, as in the lung‐to‐brain comparison,^19^ are particularly vulnerable.

We have applied these standards uniformly. Where the original studies did not report a control, we say so rather than assume it was performed, and the detection tier assigned in Tables 2, 11 and 3 reflects what was demonstrated rather than what was asserted.

### 5.9 Synthesis

The gut–lung–brain compartmental axis provides the anatomical and physiological substrate on which the microbiome–brain metastasis axis is proposed to operate. Gut‐derived signals reach lung and brain through systemic circulation and immune trafficking; lung tumour microbes are hypothesized to contribute to local immunity and metastatic potential through PAMP signalling, immune modulation and intracellular bacterial persistence, a mechanism demonstrated in other tumour types (E2) but not measured in lung cancer; and it has been hypothesized that intratumoral microbes, particularly intracellular bacteria, accompany metastasizing tumour cells to the cerebral compartment. This is not a demonstrated fact. It is a hypothesis derived from D1 detection in unpaired specimens, supported by analogy to other tumour types, and it will remain a hypothesis until within‐patient paired sampling, spatial localization, viability assays and experimental perturbation have been performed. The compartmental view also clarifies therapeutic stratification: interventions targeting gut microbiome (probiotics, FMT, dietary modulation) versus lung microbiome (inhaled antimicrobials, anti‐inflammatory bronchoscopy preparations) versus intratumoral microbiome (selective antimicrobial therapy combined with ICI) may have differential utility depending on the dominant axis node in individual patients, a stratification that is at present a design proposal rather than a clinical strategy (Section 8.8). The next section reviews clinical evidence in NSCLC brain metastasis cohorts that bears on components of this axis.

## 6 CLINICAL EVIDENCE: MICROBIOME AND BRAIN METASTASIS IN NSCLC

### 6.1 Epidemiology of brain metastasis in NSCLC

Brain metastasis is among the most common metastatic complications^1^, ^2^ of NSCLC, occurring in 30–50% of patients with locally advanced or metastatic disease over the course of their illness. Risk factors include younger age, adenocarcinoma histology, EGFR and ALK driver alterations, larger primary tumour size, and lymph node involvement. Median overall survival after brain metastasis diagnosis is 7–12 months in unselected populations,^6^ with longer survival in patients with limited intracranial disease, targetable driver alterations, and preserved performance status, and is estimated in practice with the Lung‐molGPA index, which combines age, performance status, extracranial metastasis, number of CNS lesions and EGFR/ALK status.^141^ Local therapy and CNS‐active systemic agents have improved outcomes: pembrolizumab is standard first‐line therapy in PD‐L1‐high NSCLC,^142^ and a dedicated trial has reported intracranial activity in patients with untreated brain metastases.^143^ These advances have improved outcomes without changing the fundamental biology of cerebral colonization. Prevention of brain metastasis, rather than treatment after its occurrence, represents a major unmet need. Whether microbiome‐based stratification could contribute to meeting it is an open research question, and we use the word prevention in this Review only in that sense: as an objective for future trials, never as a description of a currently available strategy.

### 6.2 Gut microbial profiles differ between lung cancer patients with and without brain metastasis

Two recent single‐centre metagenomic studies from the same research group at Dalian Medical University have begun to address whether gut microbiome composition differs between lung cancer patients with versus without brain metastasis. Liu et al. (2025) profiled faecal samples from NSCLC patients who already had brain metastasis (*n* = 18) and NSCLC patients without distant metastasis (*n* = 12), collected at a single time point after the metastasis was established rather than before it, reporting enrichment of *Paenibacillus*, *Fournierella*, and *Adlercreutzia* in the brain metastasis group; notably, α‐diversity was higher (not lower) in the brain metastasis group, and metabolomic analysis revealed altered short‐chain fatty acid and immune‐modulatory metabolite profiles, including angiotensin (1–7).^20^ In parallel, Han et al. (2025) conducted a similar analysis in SCLC and used machine learning to identify *Marvinbryantia* depletion and the metabolite benzoic acid as top discriminators of brain metastasis status, with an integrated microbiome–metabolite model reaching AUC ≈ 0.80 in cross‐validation and *Streptococcus* enrichment in the brain metastasis group also reported.^21^ The two cohorts therefore agree that gut microbial communities differ in brain metastasis patients, but the specific enriched/depleted taxa are largely non‐overlapping between NSCLC and SCLC, and direction of α‐diversity differs from the dysbiosis pattern often assumed for cancer cohorts. It is essential to be exact about the inferential status of these results. The word predicts is not supportable here. In both studies the faecal sample was obtained from patients who already had brain metastases, and the comparator was patients without distant metastasis. The design can therefore identify a correlate of brain‐metastasis status, and can support a cross‐sectional classifier; it cannot show that the microbial difference existed before the metastasis, cannot support prospective prediction, and cannot distinguish a brain‐specific association from the effects of disease stage, metastatic burden, treatment exposure, cachexia, systemic inflammation, antibiotic use or diet. The comparator group compounds this: patients without distant metastasis differ from patients with brain metastasis in overall tumour burden as well as in the site of spread, so a stage‐matched extracranial‐metastatic control group would be required before any association could be called brain‐specific. The reported AUC of approximately 0.80 is an internally cross‐validated estimate in roughly 30 patients with a large candidate predictor space, which by PROBAST criteria^39^ is high risk of bias in the analysis domain and is expected to be optimistic. The quantitative point is worth stating plainly: with approximately 15 patients per group, the two‐sided 95% confidence interval around an AUC of 0.80 spans roughly 0.62 to 0.97, so the estimate is not reliably distinguishable from weak discrimination, and no confidence interval was reported. Accordingly, we describe these findings as associations with brain‐metastasis status, and the taxa as candidate biomarkers requiring prospective validation, rather than as a premetastatic signature, an early‐detection biomarker or a basis for a prevention strategy. They do motivate candidate mechanisms, and they do justify a properly designed prospective study. Further limitations apply: both NSCLC and SCLC cohorts are from the same single centre and same research group, sample sizes are modest (*n *= 30 in NSCLC; comparable in SCLC), replication in genuinely independent multi‐centre cohorts is pending, and confounding by clinical exposures (antibiotics, steroids, chemotherapy) requires careful stratification. Larger prospective multi‐centre cohorts with serial sampling beginning before brain metastasis occurs, stage‐matched extracranial‐metastatic controls, standardized CNS imaging, competing‐risk analysis and detailed exposure ascertainment are essential next steps before these signatures can be regarded as clinically actionable.

### 6.3 Gut microbiome and immune checkpoint inhibitor response in NSCLC

The gut microbiome's role in modulating ICI response is the best‐replicated finding in this field for NSCLC and other solid tumours. Multiple independent cohorts have documented that gut microbiome composition is associated with ICI efficacy,^7^, ^8^, ^9^ with *Akkermansia muciniphila* and *Faecalibacterium prausnitzii* enrichment in responders, and mouse work establishes that defined commensals, commensal consortia and single microbial metabolites are sufficient to restore or enhance checkpoint‐blockade efficacy^10^, ^11^, ^12^ (E2). Antibiotic exposure within 30–60 days before or after ICI initiation reduces response rates^130^ and shortens survival, consistent with microbiome‐mediated effects.^37^ Proton pump inhibitors similarly correlate with reduced ICI efficacy.^144^ In NSCLC patients with brain metastasis specifically, intracranial response rates to ICIs vary widely; whether any of that variation is attributable to gut microbiome composition has not been studied, and no cohort has measured intracranial response alongside microbiome profiling. It is plausible, but unproven, that the same microbiome‐modulated peripheral immune cells traffic into the cerebral compartment and shape intracranial response (E4). Whether gut microbiome composition independently predicts intracranial response (over and above extracranial response) is an important question for clinical microbiome‐immunotherapy stratification and is, at present, entirely unaddressed by the literature. Microbiome‐modulating interventions, including probiotic supplementation, faecal microbiota transplantation, and dietary fiber modulation, are now entering clinical trials as adjuvants to checkpoint blockade in NSCLC, with potential to affect intracranial disease control. We are not aware of a registered trial in this area that uses an intracranial endpoint, though this is drawn from a narrative rather than an exhaustive registry search.

### 6.4 Gut microbiome and radiotherapy response in NSCLC brain metastasis

Stereotactic radiosurgery and whole‐brain radiotherapy are mainstays of NSCLC brain metastasis management. Treatment outcomes vary substantially across patients and have not been well predicted by tumour‐ or clinical‐level factors alone. Liang et al. (2025) conducted a prospective observational study of NSCLC patients with brain metastasis receiving radiotherapy and reported that pretreatment gut microbiome composition is associated with radiotherapy response and intracranial disease control.^22^ Specific bacterial taxa including *Bifidobacterium* were enriched in responders (E3). The scope of this finding must be stated explicitly. The study population consisted of patients who already had brain metastases; the intervention was cranial radiotherapy; the endpoint was radiotherapy response. It is therefore evidence about treatment response in established intracranial disease and nothing else. It is not evidence about brain‐metastasis occurrence, about prevention, or about intracranial response to immunotherapy, and it should not be cited in support of any of those. Reference [^22^] does not support a prediction concerning intracranial ICI response; that prediction is labelled untested (Table 6). The biological basis for microbiome‐radiotherapy interaction includes microbiome‐modulated systemic inflammation that affects radiation‐induced tumour cell death, microbiome‐imprinted anti‐tumour immunity that mediates the abscopal effect, and direct effects of gut microbes on radiation toxicity (gastrointestinal, immune). If replicated, these findings would extend the gut microbiome's clinical relevance from systemic immunotherapy^145^, ^146^ to CNS‐directed local treatment; on a single unreplicated single‐centre cohort the extension is a hypothesis, not an established range of application, broadening the therapeutic context in which microbiome stratification may guide intervention. They also raise the possibility that microbiome‐modulating interventions could enhance radiotherapy efficacy in NSCLC brain metastasis patients, a hypothesis for which we are aware of no supporting study in any tumour type (E4); the gut‐microbiome literature that does support intervention is confined to immunotherapy endpoints^147^ (E3), an approach amenable to randomized trial testing in the near term, and it is worth noting that this is the axis‐relevant hypothesis closest to being testable, because the patients are identifiable, the endpoint is short‐term and the comparison is randomisable.

### 6.5 Single‐cell intratumour microbiome in NSCLC primary tumours and brain metastases

Single‐cell resolution intratumour microbiome detection has been applied to NSCLC primary tumours and to brain metastases.^19^ Because the design of this analysis determines what may be concluded from it, we state it first: the study applied the SAHMI pipeline^53^ to single‐cell RNA sequencing libraries that had been generated in earlier, separate studies and deposited publicly. The primary‐tumour and brain‐metastasis libraries come from different patients, different laboratories and different sequencing batches; they are not within‐patient matched specimens. With that established, the analysis reported three findings. First, NSCLC primary tumours show high frequencies of fungi‐associated host cells, predominantly tumour epithelium and macrophages, with specific fungal taxa associating with specific cell types. Second, brain metastases by contrast are dominated by bacteria‐associated cells, with *Streptococcus*, *Veillonella*, and several *Enterobacteriaceae* taxa prominently represented. Third, the presence of specific microbes correlates with the transcriptome of resident host cells, including T cells and macrophages, across pathways related to ribosomal RNA processing, cell cycle regulation, and immune signalling. The compositional shift from fungi‐dominant primary tumours to bacteria‐dominant brain metastases is the most striking single observation in the field and has been interpreted as implying selective dissemination, recruitment, or proliferation favoring bacterial over fungal taxa during cerebral colonization; an equally viable explanation, which the design cannot exclude, is differential reagent and batch background between the two independently generated specimen sets (Sections 4.5 and 5.8). Mechanistic interpretation requires validation in additional cohorts and complementary methodologies; in particular, low‐biomass cerebral sampling continues to require rigorous contamination controls, and the association between microbial reads and host transcriptional state is subject to the ambient‐RNA and barcode‐swapping limitations set out in Section 5.8.4. The methodological framework established by single‐cell intratumour microbiome mining is generalisable and valuable, and the appropriate next application is a prospective study collecting within‐patient paired primary and metastatic specimens with extraction blanks and orthogonal in situ confirmation.

### 6.6 Microbial features in the context of established determinants of NSCLC brain metastasis

A microbial biomarker is only interesting if it adds something to what is already measured. Brain‐metastasis risk in NSCLC is not currently a black box: several determinants are established, several are in routine clinical use, and a candidate microbial signature must be judged against them rather than in isolation. This section sets out those determinants, asks what incremental value a microbial feature could plausibly provide, and specifies how that question should be answered. Table 12 summarizes the comparison.

**TABLE 12:** Established determinants of brain metastasis in NSCLC compared with candidate microbial features, with the evidence level and the analysis required to establish incremental value.

Determinant	What is established	Measured in routine care?	Strength of evidence for BM risk	Relationship to the microbial hypothesis	Analysis required to show microbial features add value
EGFR mutation; ALK rearrangement (ROS1 grouped conventionally)	Highest cumulative BM incidence in NSCLC (40%–70% over the disease course)^4^, ^5^; no incidence series is cited here for ROS1	Yes, universally at diagnosis	Established; large and replicated cohorts	Driver status is confounded with smoking, which is itself a dominant determinant of the airway and gut microbiome; an apparent microbial association may be a proxy	Driver‐stratified analysis with a pre‐specified interaction test; the pooled association is uninterpretable
TP53 co‐alteration and brain‐metastasis genomics	Brain metastases are genomically divergent from the matched primary and carry private drivers^75^, ^148^	TP53 yes; metastasis genomics rarely	Established	Clonal divergence means a single primary biopsy under‐samples the seeding clone, which limits any tumour‐derived biomarker including intratumoral microbial profiling	Paired primary and metastasis sampling; microbial profiling of the metastasis itself
Tumour heterogeneity and clonal architecture	Branched evolution generates subclones with differing organotropism	No	Established	Not addressed by any microbiome study; intratumoral microbial sampling inherits the same sampling problem	Multi‐region sampling with paired microbial and genomic profiling
Circulating tumour cell phenotype	Brain‐metastasis‐competent CTCs carry identifiable programs; CTC‐derived models reveal drivers.^149^, ^150^ **Both cited studies are in breast cancer; no equivalent NSCLC CTC brain‐metastasis work is cited here**.	Research use only	Established in breast cancer; extrapolated to NSCLC	The most direct competitor for a predictive blood‐based assay, and mechanistically closer to the event being predicted	Head‐to‐head comparison in the same cohort; a microbial feature should be tested as an addition to, not a replacement for, CTC or ctDNA
Blood–brain barrier transmigration and angiogenesis	Required for colonization; mediators partly characterized^1^, ^107^	No	Established	This is the step the metabolite node claims to modulate, but barrier function has never been measured in a lung cancer cohort	Contrast‐enhanced permeability imaging measured alongside metabolite quantification
Immune escape in the cerebral compartment	Microglial and T‐cell surveillance is rate‐limiting^108^	No	Established	The immune‐trafficking node predicts that microbiome shapes this, but no human measurement exists	Paired peripheral immune phenotyping with CNS outcome; MAIT and MDSC frequency as pre‐specified covariates
Lung‐molGPA	Validated prognostic index after BM diagnosis^141^	Yes	Validated prognostic index	Provides the base model against which any microbial feature must be benchmarked	Base model versus base model plus microbial feature: ΔAUC with a confidence interval, calibration, and decision‐curve net benefit^55^; continuous NRI and IDI^151^ reported descriptively only, not as decision criteria (Section 6.6.2)
**Gut microbial composition**	Differs cross‐sectionally between patients with and without BM^20^, ^21^	No	**E3, single centre, unreplicated**	The candidate feature under evaluation. Direction of α‐diversity change is opposite to the usual cancer dysbiosis assumption, and taxa do not overlap between cohorts	Prospective sampling before BM occurs; stage‐matched extracranial‐metastatic controls; competing‐risk analysis^152^; incremental value over the base model
**Plasma microbial metabolites**	Altered profiles reported in BM patients^20^, ^21^	No	**E3, single centre, unreplicated**	Closer to function than taxonomy and dynamically measurable, which makes it the more plausible surveillance marker of the two	Serial measurement with targeted quantification, analysed as **landmark or joint (dynamic‐prediction) models** so that each prediction uses only data available at that landmark; a time‐varying Cox model answers an etiologic question and cannot generate a baseline prediction. Reclassification across an imaging‐interval threshold
**Intratumoral microbial profile**	Detected in NSCLC primaries and metastases^19^, ^34^	No	**E3, detection tier D1 only**	Requires tissue, inherits the clonal sampling problem, and has not advanced beyond nucleic‐acid detection	Reach D2 by in situ localization before any predictive claim is evaluated

*Note*: The E1–E4 levels defined in Section 2.5 apply to microbial and microbial‐metabolite claims, and are therefore used only for the three microbial rows; the established determinants are described in plain evidential terms.

Abbreviations: ΔAUC, change in area under the receiver operating characteristic curve; CTC, circulating tumour cell; ctDNA, circulating tumour DNA; IDI, integrated discrimination improvement; Lung‐molGPA, lung cancer graded prognostic assessment using molecular markers; NRI, net reclassification improvement.

#### 6.6.1 What is already established

**Oncogenic drivers**: EGFR mutation and ALK rearrangement carry the highest cumulative brain‐metastasis incidence in NSCLC, in the range of 40%–70% over the disease course,^4^, ^5^ and are determined routinely at diagnosis. ROS1‐rearranged disease is conventionally grouped with them, but we are not aware of a cited incidence series for it and do not attach a figure to it here. **TP53 and the genomics of the metastasis itself**: Whole‐exome analyses of resected brain metastases show that the intracranial lesion is genomically divergent from the matched primary, harbors private driver alterations, and is enriched for specific pathway alterations in lung adenocarcinoma^75^, ^148^; brain metastases are therefore not passive copies of the primary tumour, and any model of brain‐metastasis risk must accommodate clonal evolution. **Tumour heterogeneity and clonal architecture**: Branched evolution generates subclones with differing organotropism, so a single primary‐tumour biopsy under‐samples the very clone that will seed the brain. **Circulating tumour cell phenotype**: CTCs competent for brain metastasis carry identifiable phenotypic and transcriptional programs, and CTC‐derived models reveal brain‐metastasis drivers directly^149^, ^150^; this is the most direct available window onto the disseminating population. **Blood–brain barrier penetration and angiogenesis**: Colonization requires transmigration and subsequent vascular co‐option or angiogenesis, processes with their own well‐characterized mediators.^1^, ^107^ **Immune escape**: The cerebral compartment imposes distinct immune selection, and escape from microglial and T‐cell surveillance is a rate‐limiting step. **Prognostic indices**: Lung‐molGPA^141^ integrates age, performance status, extracranial disease, lesion number and EGFR/ALK status and is in routine use for prognosis after diagnosis of brain metastasis.

#### 6.6.2 What a microbial feature would have to add

Against this background, the relevant question is not whether gut microbial composition differs between patients with and without brain metastasis; it plainly does in the cohorts reported.^20^, ^21^ The question is whether it differs after conditioning on stage, driver genotype, metastatic burden, and treatment and medication exposure, and whether adding it to a model containing those variables improves discrimination, calibration and clinical utility. None of the published studies performed this analysis. Neither Ref. 20 nor Ref. 21 reports a multivariable model adjusted for driver genotype or stage, and neither reports the incremental performance of the microbial feature over a clinical baseline model.

The analysis required is standard and inexpensive once the data exist. A base model should be fitted from established variables (histology, stage, driver genotype, number of extracranial sites, Lung‐molGPA components); the candidate microbial or metabolite feature should then be added; and the increment should be reported as change in the area under the receiver operating characteristic curve **with a confidence interval**, and, most usefully for a surveillance decision, as decision‐curve net benefit^55^ across the range of thresholds at which a clinician would actually change imaging frequency. Continuous net reclassification improvement and integrated discrimination improvement^151^ may be reported descriptively, but should not be used as decision criteria: the category‐free NRI is not a proper scoring rule, it can favor a miscalibrated model, and its null distribution is not centred on zero in finite samples, so a positive value is not evidence of improvement. Discrimination alone is insufficient: a model that is well discriminating but poorly calibrated will mis‐assign absolute risk and therefore mis‐assign surveillance intensity. Because patients with advanced NSCLC die during follow‐up, brain‐metastasis incidence must be analysed as a competing‐risks problem, with a Fine–Gray subdistribution model for the cumulative incidence alongside a cause‐specific hazard model.^152^ Death before brain metastasis is the competing event; extracranial progression is not, because a patient who progresses extracranially remains at risk of brain metastasis. Extracranial progression, and the antibiotic, corticosteroid and proton pump inhibitor exposures that accompany it, are post‐baseline variables that are simultaneously confounders and consequences of the evolving disease state, so ordinary time‐varying covariate adjustment is biased for them; they belong in a marginal structural model with inverse‐probability‐of‐treatment weighting,^153^ fitted on the cause‐specific hazard scale, with the Fine–Gray model restricted to baseline covariates for the cumulative‐incidence estimate. Analyzing the brain‐metastasis endpoint with a naive Kaplan–Meier estimator, as is common, will overstate its incidence. Two failure modes specific to this literature must also be pre‐specified: false‐discovery‐rate control for any feature‐selection step, with selection performed inside the cross‐validation loop rather than before it, since selection on the full dataset is the commonest source of optimistic internal validation; and analysis of taxonomic abundance on a scale appropriate to compositional data, since relative abundances are constrained to sum to one and ordinary correlation and regression on them are not valid. Reporting should follow the TRIPOD statement,^154^ and the study should be designed so that a PROBAST^39^ appraisal would not find high risk of bias in the analysis domain—an adequate events‐per‐candidate‐predictor ratio, pre‐specified handling of missing data, reported calibration, and a pre‐specified external validation cohort. PROBAST rates a completed study rather than a planned one, so it is a design target only in that sense.

#### 6.6.3 Do the associations differ across molecular subtypes?

This is unknown, and it matters for two reasons. First, driver genotype and microbiome are not independent: EGFR‐mutant adenocarcinoma arises disproportionately in never‐smokers, and smoking is among the strongest environmental determinants of both the airway and gut microbiome, so an apparent microbial association with brain metastasis could be a smoking‐mediated proxy for driver genotype. Second, the treatment trajectories differ: EGFR‐ and ALK‐positive patients receive tyrosine kinase inhibitors with variable CNS penetration and rarely receive first‐line ICI, whereas driver‐negative patients typically receive chemoimmunotherapy, with different antibiotic, corticosteroid and proton pump inhibitor exposure. Any subtype‐specific microbial association must therefore be separated from subtype‐specific treatment exposure. Published cohorts^20^, ^21^ are far too small to stratify by driver genotype, and we recommend that future studies pre‐specify driver‐stratified analysis with interaction testing rather than reporting a single pooled association.

#### 6.6.4 Could a microbial biomarker improve CNS surveillance?

Current practice conditions the frequency of surveillance brain MRI on histology, stage, driver status and symptoms, and the interval is a compromise between detecting asymptomatic disease when stereotactic radiosurgery is still feasible and the cost and burden of repeated imaging. A biomarker could improve this only by reclassifying patients across a threshold at which the imaging interval would actually change. That is a demanding requirement, and it is more likely to be met by a dynamic marker, measured repeatedly, than by a single baseline profile; a rising plasma kynurenine‐to‐tryptophan ratio or a falling butyrate‐producer abundance in a patient under surveillance is a more plausible trigger than a static admission profile. It is also worth stating that circulating tumour DNA is a direct, well‐characterized competitor for this role and is considerably closer to clinical use. A realistic near‐term objective for microbial features is therefore not to replace molecular risk models but to be tested as an addition to them, in the same cohort and against the same endpoint, with the incremental analysis above. We are not aware of any study that has done this, and the decision pathway of Section 8.8 embeds this comparison as its first decision node precisely for this reason.

### 6.7 Histology‐specific microbial signatures and brain metastasis risk

Lung cancer histological subtypes differ substantially in microbial associations and brain metastasis biology. NSCLC adenocarcinoma, comprising approximately 50% of NSCLC, is associated with *Tannerella*, *Veillonella*, and oral commensal enrichment in tumour tissue and shows the highest rates of brain metastasis among NSCLC subtypes (especially in EGFR‐mutant and ALK‐positive subgroups). Whether comparable signatures exist in squamous cell carcinoma or in SCLC has not been examined with a brain‐metastasis endpoint. These adenocarcinoma‐associated signatures (E3) suggest that microbiome‐based risk stratification and intervention may need to be calibrated by histology. Driver mutation overlay further refines stratification: EGFR‐mutant and KRAS‐mutant adenocarcinomas are proposed to differ in lung tumour microbial composition in ways that might interact with brain metastasis risk through differential immune signalling; we are not aware of a study that has actually compared lung tumour microbial profiles by driver genotype, and this statement is therefore E4; as noted in Section 6.6.3, this apparent interaction is confounded by smoking status and by subtype‐specific treatment exposure, and no study has separated these. A practical implication is that microbiome‐based interventions may have differential efficacy across NSCLC histologies and driver mutation subgroups, requiring stratified clinical trial designs.

### 6.8 Clinical confounders: Antibiotics, corticosteroids and proton pump inhibitors

Several clinical exposures substantially modify the microbiome in NSCLC patients and must be carefully considered in clinical interpretation and intervention design. These are not minor nuisance variables; in a cross‐sectional comparison between patients who have brain metastases and patients who do not, they are systematically imbalanced by indication and are capable of generating the entire observed association. Broad‐spectrum antibiotics, used frequently in oncology for febrile neutropenia, pulmonary infections, and prophylaxis, profoundly disrupt gut microbiome composition for weeks to months (E2). Antibiotic exposure within 30–60 days of ICI initiation reduces response rates^37^; whether it increases brain metastasis incidence has not been studied. Corticosteroids, used for cerebral edema management,^155^ ICI‐induced adverse events, and supportive care, alter both microbiome composition and host immunity in ways that may abrogate microbiome‐mediated effects. Steroid use for cerebral edema is particularly common and unavoidable in NSCLC brain metastasis patients, which means that in any cross‐sectional comparison the brain‐metastasis group is systematically more likely to be steroid‐exposed than the comparator group. This is a structural confounder of the design used in Refs. 20 and 21, not a hypothetical one. Proton pump inhibitors, frequently prescribed for symptom management, elevate gut *Streptococcus* and reduce ICI efficacy; we note that *Streptococcus* enrichment is among the reported brain‐metastasis‐associated findings,^21^ and that PPI exposure was not reported in that cohort. Chemotherapy regimens variably affect microbiome composition, with platinum agents and pemetrexed having distinct effects from immune checkpoint inhibitor regimens. Stratifying for these confounders in observational studies, and minimizing them where clinically appropriate, is essential for accurate microbiome‐outcome inference; prospectively, they should be captured as time‐varying exposures rather than as baseline covariates, since their timing relative to sampling determines their effect. Antibiotic stewardship programs in oncology, designed to minimize unnecessary antibiotic exposure while preserving appropriate prophylaxis and treatment, represent a potential intervention with axis‐level relevance.

### 6.9 Disease‐specific signatures versus convergent mechanisms

Across lung cancer subtypes (NSCLC adenocarcinoma, squamous cell carcinoma, SCLC) and across geographic and ethnic populations, specific microbial taxa associated with brain metastasis differ substantially between cohorts: Liu 2025 reported *Paenibacillus*, *Fournierella*, and *Adlercreutzia* enrichment with higher α‐diversity in NSCLC brain metastasis patients,^20^ whereas Han 2025 reported *Marvinbryantia* depletion and *Streptococcus* enrichment in SCLC brain metastasis patients.^21^ Convergent functional themes—altered SCFA‐related metabolism, altered tryptophan and benzoate pathways, and altered overall community structure—nevertheless emerge across these single‐centre studies, and are functionally consistent with the axis‐level framework articulated in Section 4: dysbiosis‐driven impaired BBB function, primed neuroinflammation, and altered anti‐tumour immunity. We note the weakness of this style of argument. When two studies identify non‐overlapping taxa, describing the results as convergent at the functional level is an interpretation that is difficult to falsify, particularly when the functional layer was inferred rather than measured. The honest summary is that the two cohorts disagree at the taxonomic level, that whether they agree at the functional level cannot be determined without directly quantified metabolites in both, and that the appearance of convergence may reflect the flexibility of pathway‐level description rather than a shared biology. The disease‐specific taxa likely reflect different upstream drivers (driver mutations, smoking exposure, dietary patterns, ethnic genetic background, and methodological differences across studies) but may or may not converge on overlapping physiological outputs. Future multi‐centre studies integrating across histologies, populations, and analytical pipelines, with harmonized protocols and centrally quantified metabolites, will be required to determine which convergent themes are robust signal versus methodological artifact.

### 6.10 Critical limitations of the current evidence base

The direct human evidence linking the gut and intratumoral microbiome to NSCLC brain metastasis rests on a small number of recently published studies that, while convergent in concept, have important methodological constraints that must be made explicit. First, the two metagenomic‐metabolomic discovery cohorts in lung cancer brain metastasis^20^, ^21^ are single‐centre, single‐group studies (Dalian Medical University; same corresponding author), with cohort sizes of approximately 30 patients each; their findings are best interpreted as hypothesis‐generating rather than as independent replication. Second, and most consequentially for how the entire literature should be read, every human study is cross‐sectional with respect to brain‐metastasis occurrence. Sampling occurs after brain metastasis is established, so no study can determine whether microbial alteration precedes brain metastasis, prospectively predicts it, or mediates a premetastatic process. The comparator groups compound this: patients without distant metastasis differ from patients with brain metastasis in stage, tumour burden, treatment exposure, cachexia, systemic inflammation, antibiotic and corticosteroid exposure and diet, and a stage‐matched extracranial‐metastatic control group, which would be required to identify a brain‐specific association, has not been used in any study. Terms such as predicts brain‐metastasis occurrence, premetastatic signature, early‐detection biomarker and prevention strategy therefore exceed the available evidence; this Review instead uses association with brain‐metastasis status and candidate biomarker requiring prospective validation. Third, the specific taxa identified as differentially abundant between brain‐metastasis and non‐brain‐metastasis patients differ substantially between the NSCLC^20^ and SCLC^21^ cohorts, and α‐diversity direction also differs from the dysbiosis pattern commonly assumed for cancer cohorts; this taxonomic non‐overlap may reflect histology‐specific biology, methodological differences, or sampling variability, and resolution requires multi‐centre replication. Fourth, the lung‐to‐brain fungi‐to‐bacteria compositional shift is currently supported by a single study^19^ that applied the SAHMI computational pipeline to public single‐cell RNA sequencing datasets; low‐biomass contamination is a well‐recognized confounder of intratumoral microbiome detection, and—because the primary‐tumour and brain‐metastasis specimens aggregated in such reanalyses are frequently drawn from different datasets, sequencing batches, and surgical sources—the apparent kingdom‐level shift could in principle reflect differential technical and contamination backgrounds between the two sample sets rather than genuine biological selection during dissemination; confirmatory work with kingdom‐resolved spatial transcriptomics, stringent decontamination controls, matched within‐patient primary‐tumour and brain‐metastasis sampling, and orthogonal detection chemistry is essential before this observation can serve as the foundation for therapeutic intervention. Fifth, the radiotherapy‐response cohort^22^ is also single‐center, addresses a treatment‐response endpoint in established intracranial disease that is not interchangeable with occurrence or prevention endpoints, and cross‐cohort generalizability is unknown. Sixth, key clinical confounders, including antibiotic exposure, corticosteroid use for cerebral edema, proton pump inhibitor use, and chemotherapy regimen, can substantially perturb gut microbiome composition and have not been uniformly stratified for in the existing cohorts. Seventh, no published study reports the full set of contamination controls set out in Section 5.8.2, and no study in NSCLC has advanced beyond detection tier D1. Eighth, the two prediction models reported^20^, ^21^ are internally validated only, in cohorts of approximately 30 patients, without calibration reporting, and are rated high risk of bias by PROBAST. Ninth, no study has assessed whether a microbial feature adds predictive information beyond established clinical and molecular determinants (Section 6.6). Finally, none of the proposed mechanistic links between microbiome‐derived metabolites, BBB integrity, microglial state, and brain metastatic colonization has been tested end‐to‐end in a single NSCLC model system; current evidence is assembled from disease‐adjacent contexts (neurodegeneration, autoimmunity, other cancer types) and from cross‐sectional human associations. Recognizing these limitations does not diminish the conceptual value of the axis framework, but it does define the experimental agenda that must be executed before microbiome‐targeted strategies are clinically deployable for NSCLC brain metastasis prevention or treatment, and Section 7 sets out the first and most decisive part of that agenda.

## 7 TARGET VALIDATION BY GENE KNOCKOUT AND GENETIC PERTURBATION: ESTABLISHING THE SPECIFICITY OF EACH NODE

Every node of the axis described above rests on correlation, on pharmacology with off‐target liabilities, or on analogy to another disease. What converts such a node into a validated target is genetic perturbation with adequate specificity controls, and this section specifies the experiments required. It is organized as the three‐level ladder shown in Figure 6c: global deletion establishes whether a gene matters at all; conditional and cell‐restricted deletion establishes which cell type is responsible; and manipulation on the microbial side, with metabolite or ligand add‐back, establishes that the microbial variable, rather than a host developmental consequence of losing the gene, is the cause. A result that survives all three levels, in an NSCLC brain‐metastasis model, is what we have defined as E1 evidence. No node of the axis has yet been taken through this ladder. Table 8 states the program gene by gene.

**FIGURE 6:**
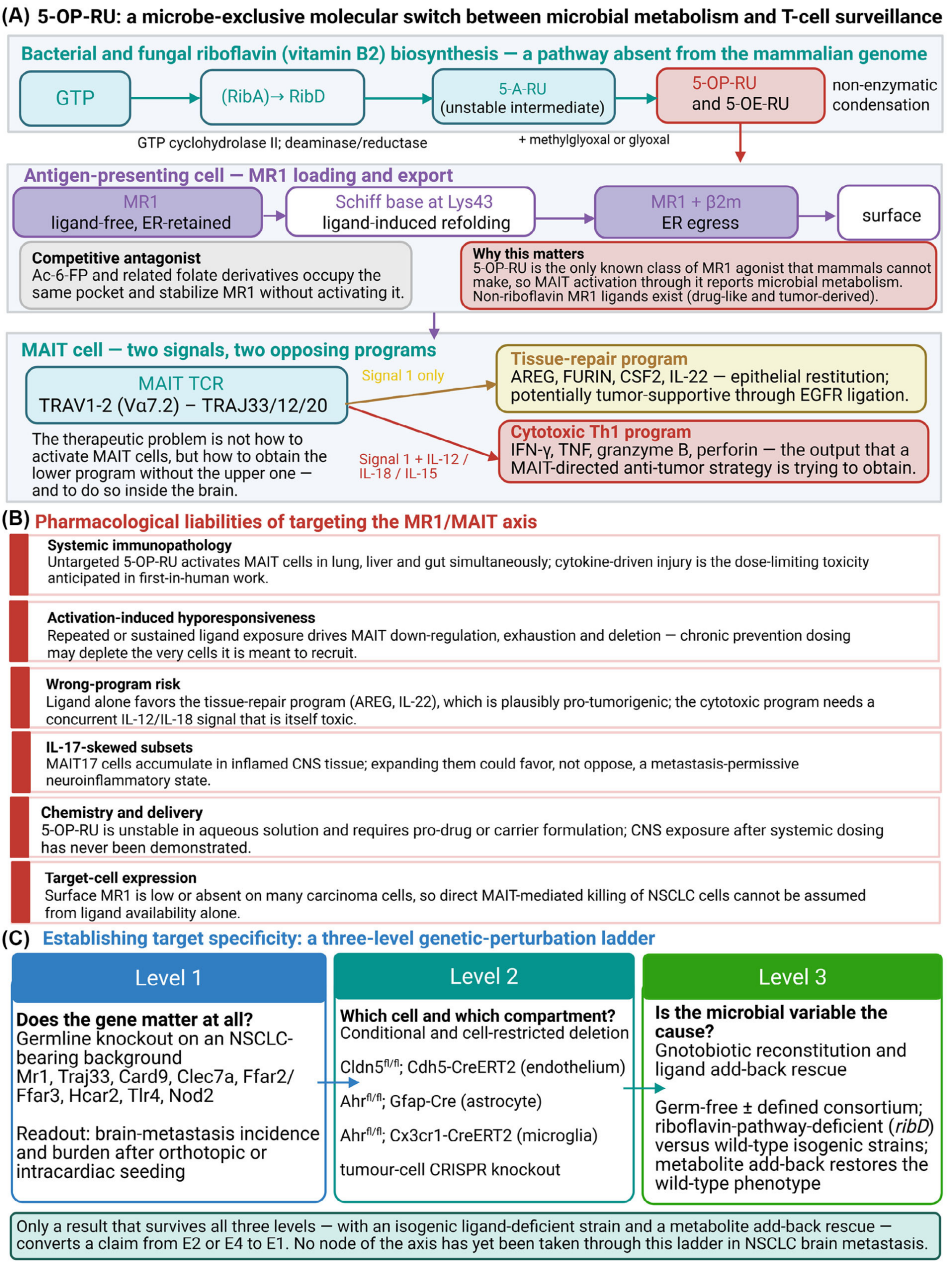
Molecular basis and genetic validation of the MR1/MAIT node, and the perturbation ladder applicable to every node. **(A)** The 5‐OP‐RU molecular switch in detail. Bacterial and fungal riboflavin (vitamin B2) biosynthesis converts GTP through RibA and RibD to the unstable intermediate 5‐A‐RU, which condenses non‐enzymatically with methylglyoxal or glyoxal to yield 5‐OP‐RU and 5‐OE‐RU.^133^ In the antigen‐presenting cell, ligand‐free MR1 is retained in the endoplasmic reticulum; ligand capture forms a Schiff base with MR1 Lys43, driving refolding, β2‐microglobulin association and export to the plasma membrane.^132^, ^134^ Recognition by the semi‐invariant MAIT T‐cell receptor then yields one of two opposing programs: T‐cell‐receptor engagement alone favours a tissue‐repair program (amphiregulin, Furin, CSF2, IL‐22), whereas concurrent IL‐12, IL‐15 or IL‐18 signalling drives the cytotoxic Th1 program (IFN‐γ, TNF, granzyme B, perforin) that an anti‐tumour strategy requires. Acetyl‐6‐formylpterin and related folate derivatives occupy the same pocket and stabilize MR1 without activating it. Because the riboflavin pathway is absent from mammalian genomes, 5‐OP‐RU is the only known class of *microbially derived* MR1 agonist and cannot be host‐derived, which is what makes this pair an unambiguous molecular switch between microbial metabolism and T‐cell surveillance; MR1 also presents non‐microbial ligands, including drug‐like small molecules^135^ and an as‐yet‐unidentified MR1‐presented ligand recognized by MR1‐restricted non‐MAIT T cells on tumour cells.^136^ **(B)** Pharmacological liabilities of targeting this axis, discussed in Section 8.5. **(C)** The three‐level genetic‐perturbation ladder described in Section 7: global deletion, conditional and cell‐restricted deletion, and perturbation of the microbial variable itself with isogenic mutants and ligand add‐back rescue. Only a result surviving all three levels, in an NSCLC brain‐metastasis model, constitutes E1 evidence. Figure prepared by the authors; no previously published material is reproduced.

### 7.1 Model systems and their limitations

Three systems are available, and each is imperfect in a way that shapes interpretation. Autochthonous models such as Kras^LSL‐G12D^;Trp53^fl/fl^ and EGFR^L858R^ recapitulate lung tumour initiation and immune context^30^, ^88^ but almost never develop spontaneous brain metastases, which is the central obstacle in this field: the endpoint of interest does not occur in the model of interest. Experimental seeding by intracardiac or intracarotid injection produces reliable and quantifiable cerebral colonization and is therefore the workhorse, but it bypasses intravasation and the early circulatory phase, so a perturbation acting on dissemination rather than colonization will be missed. Brain‐tropic derivative lines, obtained by serial in vivo selection, and patient‐derived xenografts increase relevance at the cost of requiring immunodeficient hosts, which is disqualifying for any immune node. The practical resolution is to pre‐specify which step of the cascade a given experiment addresses and to pair a seeding model, for colonization, with an autochthonous or orthotopic model, for dissemination, rather than treating either as a general‐purpose readout. Sample size should be justified against the historical variance of cerebral colonization in the specific line used, which is high, and outcome assessment should be blinded^52^.

### 7.2 Level 1: Germline deletion to establish that a gene is on the path

The first question is whether removing a candidate gene changes brain‐metastasis burden at all. For the immune nodes, *Mr1* ^−/−^ mice lack MAIT cells entirely and are the definitive test of Node 4^112^, ^132^; because MR1 is also expressed on antigen‐presenting cells and may have MAIT‐independent functions, *Mr1* ^−/−^ should be paired with *Traj33* ^−/−^ mice, which lack the Jα33 segment of the semi‐invariant T‐cell receptor and are MAIT‐deficient while retaining MR1,^156^ so that a phenotype can be attributed to the MAIT cell rather than to the presenting molecule. The direction of the expected result should not be assumed: in the one published tumour study to use these animals, *Mr1* ^−/−^ mice showed **reduced** tumour initiation, growth and experimental lung metastasis, adoptive transfer of MAIT cells restored metastasis, and the anti‐tumour effect of MR1 deficiency required NK cells, CD8+ T cells and IFN‐γ^47^ (E2). That result is the opposite of the protective role this axis attributes to MAIT cells, and it is the single most important piece of contrary evidence in the Review. The experiment must therefore be read two‐sided: an increase in brain‐metastasis burden in *Mr1* ^−/−^ animals would support Node 4 as framed here, a decrease would support the tumour‐promoting role reported in,^47^ and either outcome is informative. For cross‐kingdom sensing, *Card9* ^−/−^ and *Clec7a* ^−/−^ mice test the Dectin–CARD9 arm^81^, ^157^; both have altered gut fungal and bacterial communities at baseline, which is itself a confounder and which makes the Level 3 controls below mandatory rather than optional. For the metabolite nodes, *Ffar2* ^−/−^ ^58^ and *Ffar3* ^−/−^ ^60^ mice test SCFA receptor signalling and *Hcar2* ^−/−^ mice test the butyrate–niacin receptor,^59^ while *Tlr4* ^−/−^ and *Nod2* ^−/−^ mice test pattern‐recognition of lipopolysaccharide and peptidoglycan^67^.

Two controls determine whether a Level 1 result means anything, and both are routinely omitted in microbiome‐related mouse work. First, littermate controls generated from heterozygous intercrosses are essential: knockout and wild‐type colonies maintained separately acquire divergent microbiota, so a phenotype attributed to the gene may be a phenotype of the colony. Second, co‐housing or faecal transfer between genotypes, performed before the experiment, tests whether the phenotype is transmissible; a phenotype abolished by co‐housing is microbiota‐mediated rather than cell‐intrinsic, which for the purposes of this axis is an informative positive result rather than a failure. Cage should be modelled as a random effect, and the number of independent cages, not the number of animals, is the effective sample size.

### 7.3 Level 2: Conditional and cell‐restricted deletion to establish which cell is responsible

A global knockout cannot say where the gene acts, and for this axis the location is the biological claim. Endothelial‐restricted deletion of *Cldn5* using *Cdh5*‐CreERT2 tests whether barrier integrity is on the causal path between a microbial variable and cerebral colonization, and is the single most informative conditional experiment available for Node 3. Its limitation must be stated with it: claudin‐5 loss opens the barrier size‐selectively, to tracers below roughly 800 Da, without gross vascular disruption,^49^ so the manipulation raises small‐solute permeability but does not by itself create a route wide enough for a tumour cell. A negative result therefore falsifies the claim that small‐solute barrier leak is rate‐limiting, not the broader claim that barrier state matters; testing the latter requires a manipulation that alters junctional integrity at the scale of a transmigrating cell, for example inducible endothelial deletion of a tight‐junction scaffold together with direct measurement of transmigration. Astrocyte‐restricted (*Gfap*‐Cre) and microglia‐restricted (*Cx3cr1*‐CreERT2) deletion of *Ahr* distinguishes astrocytic from myeloid sensing of microbial tryptophan metabolites, the design that established this pathway in CNS inflammation.^27^, ^28^ Three caveats apply to these drivers and should be pre‐specified rather than discovered: *Cx3cr1*‐CreERT2 recombines in all CX3CR1‐expressing mononuclear phagocytes, so a tamoxifen‐to‐analysis interval long enough for peripheral monocytes to turn over is required before a phenotype can be called microglial; *Gfap*‐Cre is active in a subset of neural progenitors as well as astrocytes; and every conditional line needs a Cre‐only control, because Cre expression itself can alter phenotype. Myeloid‐restricted deletion of *Card9* or *Tlr4* using *Lyz2*‐Cre separates the peripheral myeloid contribution from the epithelial and lymphoid ones. Tumour‐cell‐intrinsic requirements are addressed by CRISPR knockout in the tumour line itself, with two independent single‐guide RNAs per gene, a non‐targeting guide control, and rescue by re‐expression of a guide‐resistant complementary DNA; the last of these is the control most often omitted and is what distinguishes a genuine gene effect from an off‐target or clone‐selection effect. Tamoxifen‐inducible alleles should be induced after tumour establishment where the question is colonization rather than tumour initiation, and tamoxifen‐treated Cre‐negative littermates are the correct control.

### 7.4 Level 3: Perturbing the microbial variable itself

Host‐side genetics alone cannot establish that a microbial product is the cause, because deleting a host receptor also removes its responses to endogenous ligands and, in the case of pattern‐recognition receptors, reshapes the microbiota. Three complementary approaches close this gap. **Gnotobiotic reconstitution**: Germ‐free animals colonized with a defined consortium, with faecal communities from patients with and without brain metastasis, or left germ‐free, separate microbiota‐dependent from microbiota‐independent effects; germ‐free animals have abnormal BBB and microglial baselines,^26^, ^94^ so germ‐free is a comparator, not a control. **Isogenic microbial mutants**: This is the most decisive and least‐used design. Colonizing with a riboflavin‐pathway‐deficient strain alongside its isogenic wild‐type parent tests whether 5‐OP‐RU production is required for a MAIT‐dependent phenotype. The mutation must be chosen with care: deletion of *ribD* or *ribA* blocks synthesis upstream of 5‐A‐RU and abolishes ligand formation, whereas deletion of *ribH* (lumazine synthase) acts *downstream* of 5‐A‐RU and causes the ligand precursor to accumulate, so a ΔribH strain is a gain‐of‐ligand control that should enhance rather than abolish MAIT activation, exactly as riboflavin‐pathway competence determines MAIT activation and accumulation in vivo^137^; likewise, a butyrate‐kinase‐ or butyryl‐CoA‐transferase‐deficient mutant of a butyrate producer, alongside its parent, tests whether butyrate itself rather than the organism is responsible. **Metabolite and ligand add‐back rescue**: Restoring the candidate molecule to the deficient condition, and showing that the wild‐type phenotype is restored in a dose‐dependent manner, is the final specificity control. A claim supported by add‐back rescue with an isogenic mutant comparator is qualitatively stronger than one supported by antibiotic treatment, which perturbs the entire community and has direct effects on mammalian mitochondria and on tumour cells.

### 7.5 Readouts, and the difference between a phenotype and a mechanism

Perturbation experiments in this field frequently report a difference in metastatic burden and infer a mechanism. The mechanism must be measured. We propose a minimum readout set: cerebral colonization quantified as lesion number and total burden by bioluminescence and by blinded histological counting in serial sections, since bioluminescence alone underestimates small lesions; barrier function by quantitative tracer extravasation using at least two tracer sizes, with claudin‐5 and occludin quantified by immunofluorescence and immunoblot; glial state by single‐cell or single‐nucleus RNA sequencing of microglia and astrocytes rather than by immunostaining for a single activation marker; immune trafficking by flow cytometry of brain, blood and bone marrow, with parabiosis or photoconvertible reporters where recruitment must be distinguished from local proliferation; and direct quantification of the metabolite in question in portal and systemic plasma and in brain tissue, since the inference from taxonomy to metabolite is precisely the inference this Review has argued should not be made (Section 4.3). Where the claim concerns bacteria in tissue, detection should reach at least D2 by fluorescence in situ hybridization in the perturbation experiment itself, not only in the descriptive study that motivated it.

### 7.6 What would falsify the axis

A framework that cannot be refuted is not useful, so we state the negative results that would count against each node. If germ‐free and conventionally colonized animals show equivalent cerebral colonization across two independent NSCLC lines, Node 1 fails. If *Cldn5* endothelial deletion changes barrier permeability without changing cerebral colonization, barrier integrity is not rate‐limiting and Node 3 fails as a therapeutic target irrespective of any metabolite association. If *Mr1* ^−/−^ and *Traj33* ^−/−^ animals show unchanged brain‐metastasis incidence, Node 4 fails and the MR1/MAIT therapeutic program described in Section 8.5 should not proceed. If within‐patient paired primary and metastatic specimens with extraction blanks and in situ confirmation show no kingdom‐level difference, the central observation motivating Node 2 was technical and Node 2 fails. We regard the last of these as the single most important experiment in the field, because it is inexpensive, decisive, and can be performed with existing surgical material.

## 8 TRANSLATIONAL IMPLICATIONS: MICROBIOME‐MODULATING STRATEGIES AND WHERE THEY STAND

Before describing individual interventions, we restate the framing that governs this section. None of the strategies below is validated for brain‐metastasis prevention in NSCLC, and none should be offered outside a clinical trial for that indication. GRADE labels are not used here, because no GRADE assessment was performed and the comparative evidence required for one does not exist (Section 2.5); each intervention is instead annotated with its evidence level (E1–E4) for the brain‐metastasis endpoint specifically, and with a translational‐readiness category TR0 to TR4 defined in Table 4. Where an intervention has stronger evidence for a different endpoint, such as immunotherapy response, we say so and keep the two endpoints separate.

### 8.1 Probiotic adjuvants to immune checkpoint inhibitor therapy

Probiotic adjuvants to ICI therapy represent the most clinically advanced microbiome‐modulating intervention in oncology. *Akkermansia muciniphila*,^158^, ^159^ *Bifidobacterium longum*, and *Faecalibacterium prausnitzii* have shown efficacy in preclinical models of melanoma, NSCLC, and colorectal cancer, via CD8+ T cell activation, DC maturation, and TAM polarization, and a defined 11‐strain consortium is sufficient to induce interferon‐γ‐producing CD8 T cells and to enhance checkpoint‐blockade efficacy in mice.^12^ Early‐phase NSCLC trials of probiotics combined with PD‐1/PD‐L1 inhibitors have produced mixed but generally encouraging signals. For brain metastasis prevention specifically, probiotic adjuvants have not been tested in randomized trials, but the convergent biology (improved gut barrier function, reduced systemic LPS, enhanced anti‐tumour immunity) provides mechanistic rationale. Standardized formulations and dosing for cancer indications remain under development, and live biotherapeutic products carry a real, if uncommon, risk of bacteraemia in neutropenic or immunocompromised patients that must be reflected in eligibility criteria. **Evidence: E2 in mice and E3 in humans for ICI augmentation; E4 for brain metastasis, for which no data exist. Translational readiness: TR3 for ICI augmentation, TR1 for brain metastasis**. High‐priority next studies include randomized trials of *Akkermansia*‐containing formulations in EGFR‐mutant NSCLC patients at high brain metastasis risk, with the brain‐metastasis endpoint analysed as a competing‐risks cumulative incidence.

### 8.2 Faecal microbiota transplantation in NSCLC

Faecal microbiota transplantation (FMT) transfers complete gut communities from healthy or ICI‐responder donors. Two defined live biotherapeutic products derived from donor stool are now licensed for prevention of recurrent *C. difficile* infection, while conventional FMT is performed under regulatory enforcement discretion rather than as an approved product^160^; FMT is being tested across multiple cancer indications; single‐arm phase I and phase II studies of responder‐donor FMT in anti‐PD‐1‐refractory melanoma^41^, ^42^ reported objective responses in a minority of previously refractory patients, without a randomized comparator. NSCLC FMT trials are ongoing with preliminary feasibility data. For brain metastasis prevention specifically, FMT is mechanistically plausible (restoring diversity, SCFA production, immune homeostasis to reverse dysbiosis signatures associated with brain metastasis^20^, ^21^) but untested, and we note that the direction of the intervention is not obvious from the data: α‐diversity was reported as higher, not lower, in the NSCLC brain‐metastasis group,^20^ so a diversity‐restoring intervention does not follow straightforwardly from the observed association. **Evidence: E3 for ICI‐refractory melanoma; E4 for NSCLC brain metastasis. Translational readiness: TR2 for cancer generally, TR0 for brain metastasis**. Donor screening for transmissible pathogens is essential; capsule‐based formulations are now standard, and fatal transmission of multidrug‐resistant organisms has occurred in immunocompromised recipients, which places FMT firmly in the category of interventions that require a demonstrated benefit before being contemplated for a preventive indication.

### 8.3 Antibiotic stewardship in NSCLC oncology

Antibiotic exposure during the peri‐ICI period reduces ICI response and survival in NSCLC.^37^ Antibiotic stewardship—narrow‐spectrum selection, shorter durations, deferral of non‐urgent prophylaxis around ICI initiation, minimization of fluoroquinolone use, and microbiome‐protective probiotic co‐administration when antibiotics are unavoidable—is a low‐cost, high‐impact axis‐aligned intervention. For brain metastasis prevention specifically, the rationale is that antibiotic‐induced dysbiosis may accelerate a permissive premetastatic state through butyrate‐producer depletion and immune dysregulation; this rationale is E4 and the relevant experiment, antibiotic exposure in a mouse NSCLC seeding model, has not been performed (Table 8). **Evidence: E3 for ICI outcomes in NSCLC, with consistent effects across multiple independent cohorts; E4 for brain metastasis. Translational readiness: TR4 for ICI outcomes, since stewardship is already recommended practice, and TR1 for brain metastasis**. Stewardship is uniquely tractable because it requires no new pharmaceutical development, is implementable in existing oncology infrastructure, and aligns with broader antimicrobial resistance goals. It is also the only intervention in this section that we would regard as reasonable to pursue now, and that is because its justification rests on the immunotherapy evidence rather than on the brain‐metastasis hypothesis.

### 8.4 Dietary modulation and microbial metabolite supplementation

Dietary interventions modulate gut microbiome composition over weeks to months through fermentable‐substrate availability and microbial selection: high‐fiber and Mediterranean diets promote butyrate‐producer expansion, polyphenol‐rich diets modulate anti‐inflammatory metabolite production, and ketogenic diets affect both tumour metabolism and microbiome composition. High‐fiber diets correlate with favorable ICI response in early oncology trials,^43^ but NSCLC brain metastasis prevention has not been directly tested. Direct supplementation of microbial metabolites is a complementary strategy: SCFA precursors (inulin, resistant starch, butyrylated starch) have been tested in human gut‐health trials but not, to our knowledge, in an oncology trial with a barrier or brain‐metastasis endpoint; their mechanistic basis is colonic regulatory T cell induction in mice^44^, ^45^ (E2); sodium butyrate shows BBB‐protective effects preclinically^26^; tryptophan/indole supplementation can engage AhR signalling at the BBB; bile acid supplementation modulates Treg function in preclinical cancer models. The biphasic dose–response of SCFAs at the barrier is a further caution: supraphysiological supplementation could in principle worsen rather than improve barrier integrity.^97^ **Evidence: E3 for diet and ICI response; E2 for metabolite effects on the barrier in rodents; E4 for any brain‐metastasis endpoint. Translational readiness: TR2 for diet, TR1 for metabolite supplementation**. Personalized nutrition integrating microbiome profiling is a candidate future direction.

### 8.5 MR1/MAIT ligand modulation: an unusually clean molecular switch with unusually difficult pharmacology

**Why this node is conceptually attractive**: Among the five nodes, the MR1/MAIT axis is the only one in which the connection between microbial metabolism and host immune surveillance is carried by a single, structurally defined molecular event. MR1 presents intermediates of microbial riboflavin (vitamin B2) biosynthesis,^132^ and the dominant agonist is 5‐OP‐RU, formed non‐enzymatically when the pathway intermediate 5‐A‐RU condenses with methylglyoxal or glyoxal^133^; potency is determined by covalent Schiff‐base formation with MR1 Lys43 and by the fit of the ribityl chain in the pocket.^134^ The decisive point is a genomic one: mammals have no riboflavin biosynthesis pathway. 5‐OP‐RU and its close congeners are therefore the only known class of *microbially derived* MR1 agonist ligand, and they cannot be made by the host. This is not the same as saying they are the only MR1 ligands: drug and drug‐like molecules modulate MAIT activation through the same pocket,^135^ and MR1‐restricted, non‐MAIT T cells recognize an as‐yet‐unidentified MR1‐presented ligand on tumour cells.^136^ What is unique to 5‐OP‐RU is its obligate microbial origin. Engagement of MR1 by an agonist is, in effect, an unambiguous report that riboflavin‐synthesizing microbes are metabolically active in the vicinity, and MAIT cells are the dedicated readout of that report. That is what makes this pair an almost ideal molecular switch connecting microbial metabolism to immune surveillance, and it is why we single it out: there is no comparable one‐to‐one mapping anywhere else in the axis. The functional consequence is established: MAIT activation and accumulation in vivo depend on microbial riboflavin synthesis, pathway‐deficient organisms failing to elicit the response their competent counterparts do,^137^ and MAIT cells confer MR1‐dependent protection against pulmonary bacterial infection.^138^ MR1 is also essentially monomorphic in humans, so unlike conventional MHC‐restricted approaches an MR1‐directed agent would not need to be matched to patient genotype (Figure 6a).

**Why the pharmacology is nevertheless difficult**: The attractiveness of a switch is not an argument that it can be safely thrown. Six factors are material (Figure 6b). *First, immunopathology from systemic activation*. MAIT cells are abundant at mucosal surfaces, in liver and in lung, and a systemically administered agonist activates them everywhere at once. In mouse models, systemic 5‐OP‐RU with a co‐stimulatory signal produces rapid MAIT expansion and cytokine release, and the relevant toxicities to anticipate in humans are acute lung injury, hepatitis and a cytokine‐release‐like syndrome; in a patient with lung cancer, pulmonary MAIT activation is precisely the toxicity one would least wish to provoke. *Second, activation‐induced hyporesponsiveness*. Repeated or sustained ligand exposure drives T‐cell‐receptor down‐regulation, functional exhaustion and deletion of MAIT cells. A prevention indication implies chronic dosing over months, which is the exposure pattern most likely to deplete the effector population the strategy depends on. *Third, and most specific to this axis, the risk of eliciting the wrong program*. Ligand engagement alone favors the tissue‐repair program, with amphiregulin, Furin and IL‐22; the cytotoxic program requires concurrent IL‐12, IL‐15 or IL‐18. Amphiregulin is an EGFR ligand, and a sustained tissue‐repair program is at least as plausibly tumour‐promoting as tumour‐suppressing in EGFR‐driven lung adenocarcinoma. Obtaining the cytotoxic program therefore requires co‐delivery of an inflammatory cytokine signal that is itself the principal source of toxicity, and this coupling, rather than ligand availability, is the central pharmacological problem. *Fourth, subset skewing*. MAIT17 and MAIT1/17 cells accumulate in inflamed CNS tissue^46^; expanding an IL‐17‐polarized population in the cerebral compartment could plausibly favor a metastasis‐permissive neuroinflammatory state rather than oppose it. *Fifth, chemistry and delivery*. 5‐OP‐RU is unstable in aqueous solution, degrading over minutes, which is why it is typically generated in situ or delivered as a precursor; a clinical agent requires a pro‐drug, a carrier or a stabilized analog, and no formulation has demonstrated CNS exposure after systemic administration. *Sixth, target‐cell expression*. Surface MR1 is low or absent on many carcinoma cells and is regulated post‐translationally by ligand availability, so direct MAIT‐mediated killing of NSCLC cells cannot be assumed even if circulating MAIT cells are successfully activated.

**What follows for development**: These liabilities argue against systemic agonism as a first clinical step and in favor of three alternatives: local or tumour‐directed delivery that restricts ligand exposure; MR1‐directed agents that act as antagonists or as stabilizers, of which acetyl‐6‐formylpterin is the prototype, where the therapeutic hypothesis is blockade of a tumour‐supportive repair program rather than induction of cytotoxicity; and cell‐based approaches in which MAIT cells are expanded and polarized *ex vivo*, so that the cytokine signal required for the cytotoxic program is delivered outside the patient. Any first‐in‐human study should include pre‐specified stopping rules for cytokine release and hepatic toxicity, serial MAIT frequency and phenotype monitoring to detect exhaustion, and a pharmacodynamic readout distinguishing the repair from the cytotoxic program. A seventh liability is that the sign of the effect is not settled: MAIT cells have been reported to **promote** tumour initiation, growth and experimental lung metastasis through tumour MR1, with MAIT‐deficient animals protected.^47^ If that is the operative direction in lung cancer, an MR1 agonist would be expected to worsen rather than prevent metastasis, and the appropriate therapeutic hypothesis would be MR1 blockade. The *Mr1* ^−/−^ and *Traj33* ^−/−^ experiments in Section 7.2 should therefore precede any of this: they establish the sign before any agonist is given, and if MAIT deficiency does not change brain‐metastasis burden in an NSCLC model, the program should not proceed.

Additional immune‐axis interventions include anti‐IL‐17 therapy for patients with elevated Th17/IL‐17 signatures, anti‐IL‐6 therapy for BBB‐permeable IL‐6‐high inflammation, and CAR‐T or BiTE approaches targeting intracellular bacterial antigens within tumour cells, the last of which has a defined molecular basis in the demonstration that bacteria‐derived peptides are presented on tumour HLA molecules.^84^ **Evidence: E2 for MAIT biology and CNS accumulation in infection and autoimmunity models; E4 for any role in NSCLC brain metastasis, where no human or animal data exist. Translational readiness: TR1**.

### 8.6 Blood‐brain barrier stabilization and premetastatic niche disruption

Pharmacological BBB stabilization is an underexplored anti‐metastatic strategy. Agents that strengthen tight junction expression (claudin‐5 modulators), suppress endothelial inflammation, or directly enhance BBB integrity could reduce metastatic extravasation; candidate agents acting on the barrier include angiopoietin‐1 analogs (investigational), sphingosine‐1‐phosphate modulators (approved for multiple sclerosis and ulcerative colitis, not for a barrier indication), and corticosteroids (with the caveat that corticosteroids also modulate microbiome and immunity in axis‐relevant ways). Combination strategies integrating microbiome modulation, BBB stabilization, and niche disruption represent the most mechanistically integrated approach but require careful sequencing and safety evaluation, and carry an obvious hazard that deserves explicit statement: an agent that tightens the barrier against tumour cell entry will also tighten it against CNS‐penetrant systemic therapy, so barrier stabilization and CNS drug delivery are directly opposed objectives in the same patient. **Evidence: Claudin‐5 is an established determinant of barrier permeability in mouse genetics** ^48^ **^,^** ^49^ **; that is host biology and therefore sits outside the E1–E4 scheme, which grades microbial claims (**Section 2.5 **). E4 for barrier stabilization as an anti‐metastatic strategy in NSCLC. Translational readiness: TR0–TR1. The decisive preclinical experiment is endothelial‐restricted *Cldn5* deletion or stabilization with cerebral colonization as the endpoint (**Table 8 **)**.

### 8.7 Personalized stratification and the axis‐aware clinical decision scheme

Personalized stratification on axis‐level features is the integrative goal of the framework. Stratification would draw on three profiling dimensions—gut and tumour microbial composition, plasma metabolite signatures, and axis‐relevant host genotype—and would route patients to microbiome‐targeted, metabolite‐targeted or immune‐axis‐targeted intervention classes (Figure 7b). The operational form of this scheme, with entry criteria, confounder exclusions and endpoints, is given in Section 8.8 and Figure 8. The scheme is conceptual; clinical validation requires axis‐stratified RCTs and standardized profiling protocols integrated with existing NSCLC decision support.

**FIGURE 7:**
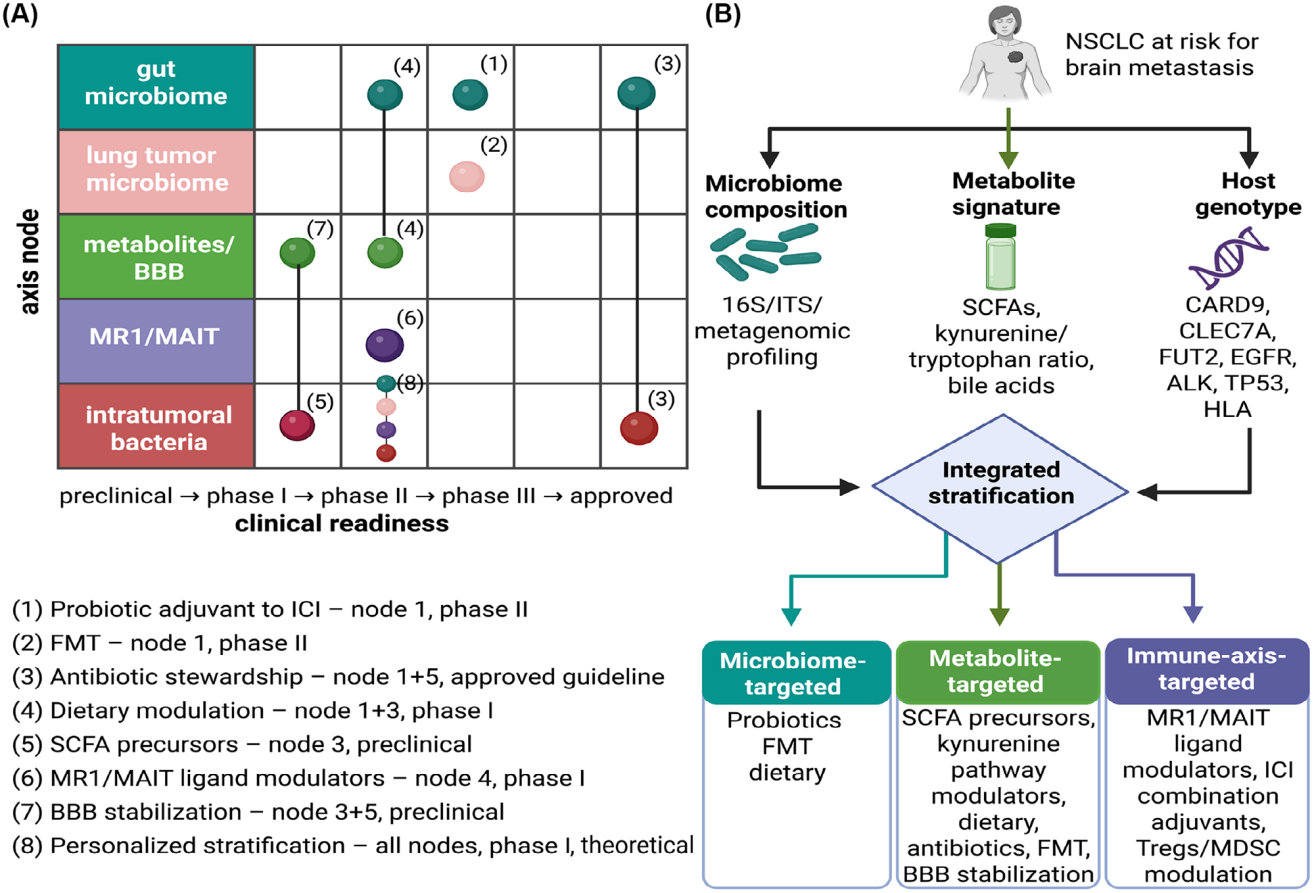
Therapeutic landscape and axis‐aware clinical decision scheme for microbiome‐modulating interventions in NSCLC brain metastasis. **(A)** Eight therapeutic strategies mapped to the five axis nodes (Node 1, gut microbiome; Node 2, lung tumour microbiome; Node 3, microbial metabolites and BBB; Node 4, MR1/MAIT immune surveillance; Node 5, intratumoral bacteria and premetastatic niche), organized by clinical readiness. Interventions span probiotic adjuvants to ICI; faecal microbiota transplantation; antibiotic stewardship; dietary modulation (fiber, Mediterranean diet, polyphenols); microbial metabolite supplementation (SCFA precursors, indoles); MR1/MAIT ligand modulation; BBB stabilization and premetastatic niche disruption; and personalized stratification combining multiple axis nodes. **(B)** An axis‐aware decision scheme for stratified intervention selection in NSCLC patients at risk for brain metastasis. The scheme is conceptual; clinical validation through axis‐stratified randomized controlled trials in NSCLC brain metastasis prevention is required. **Relationship to** Table 4. Panel a plots eight intervention classes; Table 4 lists ten. The two additional rows are intracellular antibiotic targeting of intratumoral bacteria and the ICI‐plus‐microbiome‐adjuvant combination, neither of which is plotted in panel a; Table 4, not this panel, is the complete list. **Node numbering in this figure**. The row labels on the vertical axis of panel a, and the digits in the panel's own key, belong to the original artwork and denote compartments rather than the numbered nodes used everywhere else in this Review. They map onto the Section 1.4 numbering as follows: gut microbiome = Node 1; lung tumour microbiome and intratumoral bacteria = Node 2, drawn as two rows because they are separate intervention targets; metabolites and blood–brain barrier = Node 3; MR1/MAIT = Node 4. Node 5, host genetic modification, is not itself a therapeutic target and has no row. Section 1.4, Figure 1b and Tables 4, 6, 8 use the Section 1.4 numbering throughout; this panel is the single exception. **Evidence status**. This figure depicts a research design, not clinical practice. The operational form of the scheme, with entry criteria, confounder exclusions, the decision node that must be passed before any intervention is considered, and the endpoint definition, is given in Figure 8 and Section 8.8. Panels prepared by the authors and created with BioRender.com; no previously published material is reproduced.

**FIGURE 8:**
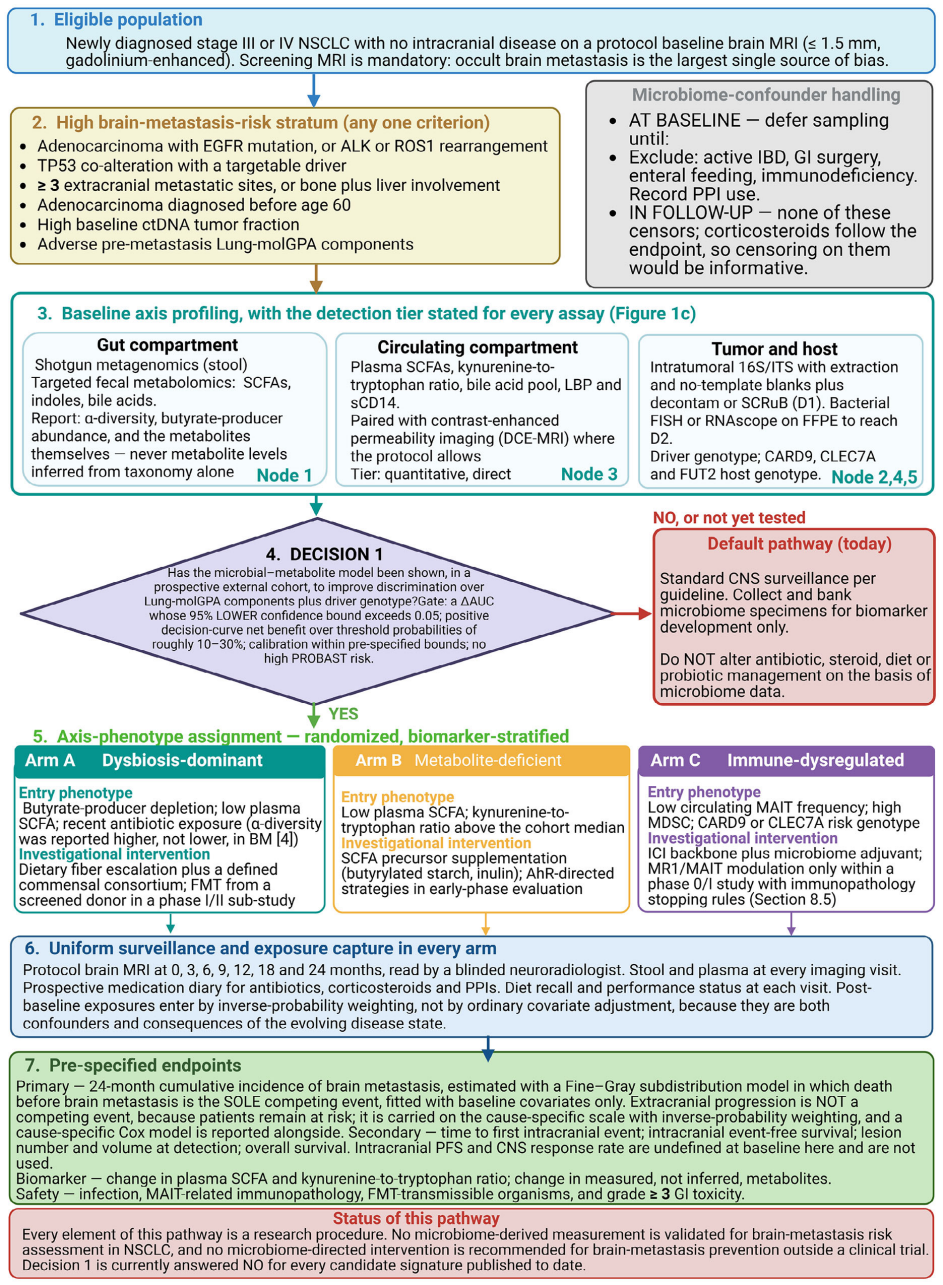
An operational, research‐only decision pathway for microbiome‐guided brain‐metastasis prevention research in high‐risk NSCLC. Step 1: eligible population, requiring a protocol baseline brain MRI to exclude occult intracranial disease. Step 2: the high‐risk stratum and the microbiome‐confounder exclusions that must be applied at enrolment. Step 3: baseline axis profiling across gut, circulating and tumour/host compartments, with the detection tier stated for every assay and with metabolites measured rather than inferred from taxonomy. Decision 1: the gate that currently blocks the pathway: whether the microbial–metabolite model has been shown prospectively to improve discrimination over Lung‐molGPA plus driver genotype. For every published signature this is answered NO, and the pathway therefore terminates in the default arm at right, standard surveillance with specimen banking and no microbiome‐directed change in management. Step 5: axis‐phenotype assignment within a randomized biomarker‐stratified trial, should the gate be passed. Step 6: uniform surveillance and time‐varying exposure capture. Step 7: pre‐specified endpoints, with the primary endpoint analysed as a competing‐risks cumulative incidence. Every element of this pathway is a research procedure; no microbiome‐derived measurement is validated for brain‐metastasis risk assessment in NSCLC and no microbiome‐directed intervention is recommended for brain‐metastasis prevention outside a clinical trial. Figure prepared by the authors; no previously published material is reproduced.

### 8.8 An operational decision pathway for the high‐risk NSCLC brain‐metastasis subgroup

The strategies set out above are, on their own, a general oncology inventory rather than a pathway tied to the specific endpoint of brain‐metastasis prevention in a defined NSCLC population. What follows is the concrete decision pathway. Figure 8 provides one. It is deliberately operational: it names who enters, what is measured and with which detection tier, what must be true before any intervention is contemplated, how patients are assigned, how they are followed, and how the endpoint is analysed. It is equally deliberately framed as a research pathway, because the honest answer to the question of what a clinician should do differently today, on the basis of this literature, is nothing.

#### 8.8.1 Who enters, and who does not

Entry requires stage III or IV NSCLC with no intracranial disease on a protocol baseline gadolinium‐enhanced brain MRI with slice thickness of 1.5 mm or less. Mandatory screening MRI is not a formality: occult brain metastasis at the time of sampling is the single largest source of bias in every cross‐sectional study reviewed here, and excluding it at baseline is what converts a cross‐sectional comparison into a prospective one. The high‐risk stratum is then defined by any one of: adenocarcinoma with an EGFR mutation or an ALK or ROS1 rearrangement; TP53 co‐alteration alongside a targetable driver; three or more extracranial metastatic sites, or combined bone and liver involvement; adenocarcinoma diagnosed before age 60; high baseline circulating tumour DNA tumour fraction; or an adverse profile on those Lung‐molGPA components that are defined before brain metastasis occurs—age, performance status, extracranial disease burden and EGFR/ALK status—noting that the Lung‐molGPA index itself is validated for prognosis after a brain metastasis diagnosis^141^ and is not applied here as a whole. Baseline eligibility and follow‐up handling are deliberately separated, because censoring on an exposure that the endpoint itself causes would be informative censoring. **At baseline**, sampling is deferred until 30 days after systemic antibiotics, 14 days after corticosteroids above 10 mg prednisone‐equivalent daily, and 90 days after probiotic or FMT exposure; active inflammatory bowel disease, recent gastrointestinal surgery, enteral feeding and immunodeficiency are exclusions, and proton pump inhibitor use is recorded rather than excluded. **During follow‐up**, none of these exposures censors the observation: corticosteroids in particular are given for cerebral edema and are therefore a consequence of the endpoint (Section 6.8), so censoring on them would remove precisely the patients who reached it. They are carried as time‐varying covariates instead. These exclusions are not fastidiousness; as Section 6.8 sets out, they are the exposures most capable of generating the association the study is trying to measure.

#### 8.8.2 What is measured

Three compartments are profiled in parallel, and the detection tier of each assay is stated in the protocol. In the gut compartment, shotgun metagenomics with extraction blanks, and targeted faecal metabolomics for short‐chain fatty acids, indoles and bile acids. In the circulating compartment, quantitative plasma short‐chain fatty acids, kynurenine‐to‐tryptophan ratio, bile acid pool, lipopolysaccharide‐binding protein and soluble CD14, paired where the protocol permits with contrast‐enhanced permeability imaging, so that the barrier hypothesis is measured rather than assumed. In the tumour and host compartment, intratumoral 16S and ITS profiling with the full control set of Section 5.8.2 to establish D1, bacterial FISH or RNAscope on archival material to reach D2, driver genotype, and CARD9, CLEC7A and FUT2 host genotype. The requirement to report the metabolite rather than infer it from taxonomy is explicit, and follows from Section 4.3.

#### 8.8.3 The decision node that currently blocks the pathway

Before any patient is assigned to an intervention, one question must be answered: does the microbial–metabolite model improve discrimination over a base model containing driver genotype, stage, histology, number of extracranial sites and those Lung‐molGPA components that are defined before brain metastasis occurs (age, performance status, extracranial disease burden, EGFR/ALK status), in a prospective external cohort, by a margin that would change a surveillance or treatment decision? The pre‐specified criteria we propose are an increase in the area under the curve **whose two‐sided 95% lower confidence bound exceeds 0.05** – a point estimate at the margin is not enough, because a small cohort will produce one by chance –, a positive decision‐curve net benefit^55^ over the threshold probabilities at which a clinician would change imaging frequency, which for a six‐monthly‐versus‐three‐monthly MRI decision we take to be roughly 10 to 30%, calibration‐in‐the‐large and calibration slope within pre‐specified bounds, and a study design that would not attract a high‐risk‐of‐bias rating in the PROBAST^39^ analysis domain. The 0.05 margin is a convention and not a derived quantity; we state it so that it can be argued with, and we require the interval rather than the point estimate so that a small cohort cannot pass the gate on noise. We do not use continuous net reclassification improvement as a criterion, for the reasons given in Section 6.6.2. For every candidate signature published to date, this question is answered NO, because the analysis has not been performed (Section 6.6.2). The consequence, shown explicitly in Figure 8, is that the default pathway today is standard CNS surveillance with specimen banking for biomarker development, and no alteration of antibiotic, corticosteroid, dietary or probiotic management on the basis of microbiome data.

#### 8.8.4 Assignment, follow‐up and endpoint

If and when that decision node is passed, axis phenotype defines the stratum and, within each stratum, patients are randomized 1:1 between the stratum‐specific intervention and standard care; the primary comparison is intervention versus standard care, pooled across strata with stratum as a pre‐specified subgroup. The phenotype itself is a stratification variable, not a randomized assignment. The strata are: Arm A, dysbiosis‐dominant, defined by butyrate‐producer depletion, low plasma short‐chain fatty acids and recent antibiotic exposure rather than by low α‐diversity, since α‐diversity was reported as higher, not lower, in the NSCLC brain‐metastasis group,^20^ receiving dietary fibre escalation and a defined commensal consortium, with FMT confined to a phase I/II sub‐study; Arm B, metabolite‐deficient (low plasma short‐chain fatty acids, elevated kynurenine‐to‐tryptophan ratio), receiving short‐chain fatty acid precursor supplementation; Arm C, immune‐dysregulated (low circulating MAIT frequency, high MDSC, CARD9 or CLEC7A risk genotype), receiving an ICI backbone with a microbiome adjuvant, with MR1/MAIT modulation only within a phase 0/I study carrying the immunopathology stopping rules of Section 8.5. Surveillance is uniform across arms: protocol brain MRI at 0, 3, 6, 9, 12, 18 and 24 months read by a blinded neuroradiologist, stool and plasma at every imaging visit, and a prospective medication diary capturing antibiotics, corticosteroids and proton pump inhibitors as time‐varying exposures. The primary endpoint is the 24‐month cumulative incidence of brain metastasis estimated by a Fine–Gray subdistribution model with **death before brain metastasis as the sole competing event**,^152^ fitted with baseline covariates only, reported alongside a cause‐specific hazard model; analysing this endpoint without a competing‐risks framework will overstate incidence, because patients in this population die at high rates. Extracranial progression is *not* a competing event, because patients who progress extracranially remain at risk of brain metastasis; it is modelled on the cause‐specific scale as a time‐varying exposure, and because it is both a confounder and a consequence of earlier treatment, it is handled with inverse‐probability weighting in a marginal structural model^153^ rather than by ordinary covariate adjustment (Section 6.6.2). The same applies to antibiotic, corticosteroid and proton pump inhibitor exposure. Secondary endpoints, defined for a population that has no intracranial disease at baseline, are time to first intracranial event (new lesion or leptomeningeal disease), intracranial event‐free survival, number and total volume of brain metastases at first detection, overall survival, and—among those who develop brain metastasis and therefore acquire measurable intracranial target lesions—subsequent intracranial response to protocol‐directed therapy. Intracranial progression‐free survival and CNS‐specific objective response rate are not defined at baseline in this population and are not used. Safety capture is pre‐specified for infection, MAIT‐related immunopathology and transmissible organisms. **Size**: A trial of this design is not small, and it is better to say so than to leave it implied. Taking a 24‐month cumulative incidence of brain metastasis of approximately 30% in the high‐risk stratum—the figure implied by the 40%–70% lifetime incidence in driver‐positive adenocarcinoma^4^, ^5^ over a 24‐month horizon, and the single assumption most in need of confirmation from registry data before any protocol is written—a reduction to 18% (a 40% relative reduction) requires approximately 195 patients per arm for 80% power at a two‐sided α of 0.05, or approximately 230 per arm after inflation for loss to follow‐up and incomplete 24‐month follow‐up. No separate inflation is applied for death, because a 24‐month cumulative incidence is a subdistribution quantity already defined in the presence of that competing risk. A more modest reduction, to 20%, requires approximately 290 per arm before inflation and approximately 340 after the same allowance. A two‐arm study is therefore a 500‐patient undertaking and a three‐arm biomarker‐stratified study is larger still, which is the honest reason that such a trial has not been done and the reason the decision gate of Section 8.8.3 must be passed before it is attempted.

## 9 CONCLUSION AND TRANSLATIONAL ROADMAP

Brain metastasis remains the most lethal complication of NSCLC, and existing therapies have only modestly improved outcomes. The microbiome–brain metastasis axis is an integrative framework connecting systemic microbial biology to cerebral colonization through five candidate mechanisms (circulating metabolites and BBB modulation; microbiome‐imprinted immune cell trafficking; intratumoral bacteria and brain‐tropic clone selection; cross‐kingdom MR1/MAIT and Dectin–CARD9 signalling; host genetic determinants). Recent metagenomic and single‐cell intratumour microbiome studies in NSCLC and SCLC provide initial evidence of association, but the human evidence base is restricted to small single‐centre cross‐sectional discovery cohorts and a single SAHMI‐based reanalysis of separately generated public scRNA‐seq data; this evidence is hypothesis‐generating and requires multi‐centre independent replication and mechanistic validation (Section 6.10).

Applying the grading schemes of Section 2 produces three conclusions, stated here without hedging. First, no node of the axis attains E1: no microbial or microbial‐metabolite variable has been shown to change brain‐metastasis incidence or burden in any NSCLC model. Second, every microbial claim in NSCLC brain metastasis rests on detection tier D1; no study has demonstrated microbial structures in situ, viable organisms, or a community reproducible across cohorts and chemistries. Third, all human evidence is cross‐sectional with respect to brain‐metastasis occurrence, so the literature supports statements about association with brain‐metastasis status and supports no statement about prediction, precedence or causation. The term prevention is used in this Review exclusively to name an objective for future trials. Figure 9 summarizes the axis together with these three qualifications.

**FIGURE 9:**
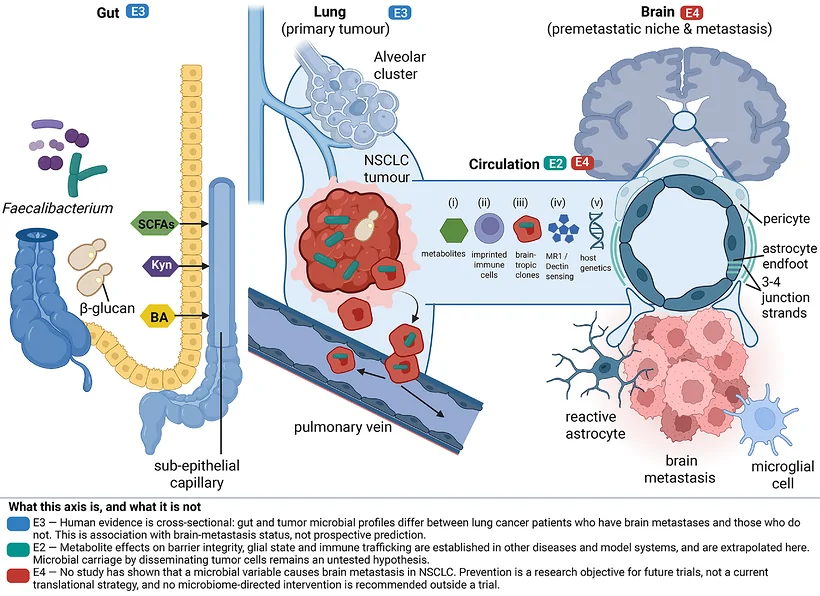
The proposed microbiome–brain metastasis axis in NSCLC, shown across the gut, lung and cerebral compartments, with the circulating intermediates (microbial metabolites, microbiome‐imprinted immune cells, brain‐tropic clones, MR1/Dectin sensing and host genetics) that are hypothesized to connect them. Evidence badges state the level attained by each compartment, as defined in Section 2.5: E3 for the gut and primary tumour compartments, where human association evidence exists; E2 and E4 for the circulating intermediates; and E4 for the cerebral compartment, where the microbial contribution is hypothesized and has not been demonstrated. The panel at the foot of the figure states what the axis is and what it is not: human evidence is cross‐sectional, no microbial variable has been shown to cause brain metastasis in NSCLC, and prevention is a research objective for future trials rather than a current translational strategy. **Data provenance**: The compartments, routes, cell types and metabolite classes drawn here are schematic. The single named genus in the gut compartment, *Faecalibacterium*, is an immune‐checkpoint‐inhibitor‐response‐associated taxon^7^, ^8^ and is not among the taxa reported by the lung cancer brain‐metastasis cohorts; those taxa are given in Figure 5a and Table 11. The repository, platform, specimen numbers and reported quality control are listed in Table 5.^19^, ^20^, ^21^ Figure prepared by the authors and created with BioRender.com; no previously published material is reproduced.

The framework yields ten falsifiable predictions with decisive readouts (Table 6). Methodological priorities for the field include kingdom‐resolved meta‐transcriptomic profiling across within‐patient paired primary‐tumour and brain‐metastasis samples with stringent contamination controls; prospective multi‐centre cohorts with serial microbiome sampling beginning before brain metastasis occurs, with stage‐matched extracranial‐metastatic controls, standardized CNS imaging, competing‐risk analysis and detailed clinical exposure ascertainment; gnotobiotic NSCLC brain metastasis models and the gene‐knockout program of Table 8; single‐cell intratumour microbiome detection integrated with anti‐tumour immunity profiling and confirmed by orthogonal in situ methods; and clinical trials of axis‐stratified microbiome‐modulating interventions combined with ICI or CNS‐directed radiotherapy. The translational roadmap involves three sequential steps: (i) validation of microbiome‐based brain metastasis risk prediction in independent multi‐centre cohorts, including the demonstration that a microbial feature adds information beyond established molecular risk models (Section 6.6); (ii) mechanistic dissection using gnotobiotic and humanized microbiome models with the three‐level specificity ladder of Section 7; (iii) intervention trials combined with standard of care, following the decision pathway of Figure 8. Whether microbiome biology is a tractable component of NSCLC brain‐metastasis prevention and treatment is, on present evidence, an open question. This Review states precisely what would be required to answer it, without implying that it has already been answered.

## AUTHOR CONTRIBUTIONS

Long Liang, Fangjuan Hu and Xiaofeng Wang contributed equally to this work and share first authorship. Churong Li, Chungang Zhao and Yin Zhang conceived and supervised the Review. Long Liang, Fangjuan Hu and Xiaofeng Wang performed the literature search, evidence synthesis, and initial manuscript drafting, with Long Liang and Fangjuan Hu screening records independently and in duplicate and Xiaofeng Wang adjudicating disagreements. Chunhan Tang, Chun Wang and Gefei Jiang contributed to literature interpretation, figure preparation, table organization and critical revision of the manuscript. Chungang Zhao, Yin Zhang and Churong Li provided clinical and translational guidance, revised the manuscript for important intellectual content, and supervised the final version. Churong Li adjudicated evidence‐level and detection‐tier assignment. All authors read and approved the final manuscript.

## CONFLICT OF INTEREST STATEMENT

The authors declare no conflicts of interest.

## CONSENT FOR PUBLICATION

The authors have nothing to report.

## ETHICAL APPROVAL AND CONSENT TO PARTICIPATE

Not applicable. This Review does not involve human participants, animal experiments, clinical samples, or unpublished patient‐level data. Consequently, no institutional ethical approval or informed consent was required. All evidence discussed is derived from previously published studies, each of which reported its own ethical approvals.

## Data Availability

No new datasets were generated or analysed during the preparation of this Review. All evidence discussed in this article is derived from previously published studies. The public data resources underlying the substantive claims of this Review are listed in Table 5, together with the platform, the number of specimens and patients, whether specimens were paired within patients, the quality control reported by the original authors and the limitation each dataset imposes. Repository identifiers are those reported by the original authors and are reproduced in Table 5 rather than re‐deposited by us; where a dataset has not been deposited, or is access‐restricted, Table 5 says so explicitly. The full search strings, screening records and data‐extraction forms described in Section 2 are available from the corresponding author on reasonable request.
